# Prenatal exposure to medication and risk of childhood cancer – a systematic review and meta-analysis

**DOI:** 10.1186/s12885-025-15316-0

**Published:** 2025-11-21

**Authors:** Alicia Lübtow, Manuela Marron, Rajini Nagrani, Loviisa Mulanje, Wolfgang Ahrens, Lara Kim Brackmann

**Affiliations:** 1 Department Epidemiological Methods and Etiological Research, Leibniz Institute for Prevention Research and Epidemiology - BIPS, Bremen, Germany; 2 Faculty of Human and Health Sciences, University of Bremen, Bremen, Germany

**Keywords:** Pharmaceutical preparations, Pregnancy, Leukemia, Neoplasms, Malignancy, Antibiotics

## Abstract

**Background:**

The use of medication during pregnancy carries a potential health risk, including childhood cancer, for the unborn child. However, most drugs examined in previous observational studies have shown inconsistent results with the risk of childhood cancer, and these findings have not been consolidated in drug-specific meta-analyses.

**Methods:**

We conducted a systematic search in the databases *PubMed* and *Web of Science* for studies on medication use during pregnancy and childhood cancer risk. Studies with exposure to diethylstilbestrol as a known teratogen were excluded. Meta-analyses were conducted if ≥ 3 studies on the same research question were available to calculate pooled estimates for random effects models with 95% confidence intervals (CI). The *I*^2^ statistic was calculated to quantify between-study heterogeneity. Statistical significance of *I*^2^ was analyzed using Q statistic (P value for heterogeneity (P)). Study quality was assessed with a scoring tool adapted from the Newcastle–Ottawa Scale and ROBINS-E/I.

**Results:**

Of 2,366 identified studies, 80 were included in the systematic review. Of these, 68 studies with a total of 14,396,922 participants were included in meta-analyses, covering 13 medication types and 11 childhood cancer sites across 70 analyses. From 54 site-specific analyses, we observed four risk reductions and eleven increases in risk. We found an increased risk for prenatal exposure to any kind of antibiotics and acute lymphoblastic leukemia (ALL; estimate (ES) = 1.14 (95%CI 1.03; 1.25), *I*^2^ = 19.1%, *P =* 0.26). For specific antibiotics, nitrosatable antibiotics were associated with an increased risk of childhood cancer overall (ES = 1.32 (1.13; 1.55), *I*^2^ = 0.0%, *P =* 0.98). Maternal intake of vitamin and mineral supplements was associated with a reduced risk of acute leukemia (AL) (ES = 0.72 (0.54; 0.96), *I*^2=^46.3%, *P =* 0.16), ALL (ES = 0.81 (0.67; 0.99), *I*^2^ = 66.6%, *P =* 0.001) and tumors of the central nervous system (CNS; ES = 0.77 (0.62; 0.96), *I*^2^ = 67.2%, *P =* 0.002).

**Discussion:**

Our results suggest that the use of antibiotics during pregnancy was associated with an increased childhood cancer risk, although this association is likely influenced by the underlying maternal infections that required treatment. This highlights the importance of considering maternal health factors when interpreting medication–related associations. Supplementation of vitamins and minerals during pregnancy might decrease the risk of AL, ALL and CNS tumors in children.

**Supplementary Information:**

The online version contains supplementary material available at 10.1186/s12885-025-15316-0.

## Background

In 2022, approximately 275,000 cases of cancer in children up to the age of 19 were diagnosed worldwide. Although childhood cancer is relatively rare, it remains the leading cause of disease-related deaths in children over the age of one year [1–3]. In contrast to adults, where solid tumors predominate, the most common cancer types in children are leukemia, lymphomas and tumors of the central nervous system (CNS) [1]. However, the underlying causes of childhood malignancies are still largely unknown [4].

Current evidence suggests that the development of childhood cancer, particularly acute leukemia (AL), is likely multifactorial, involving a complex interplay of pre- and postnatal factors [5–9]. Genetic predispositions and hereditary genetic diseases like neurofibromatosis type 1, Li-Fraumeni syndrome, Fanconi anemia, tuberous sclerosis and predisposing genes [8, 10, 11] account for only 5% to 10% of childhood cancers [12]. In addition to genetic factors, several environmental and lifestyle factors have been implicated in increasing the risk of childhood cancer. These include exposure to chemical substances like benzene and pesticides, ionizing radiation, and parental use of nicotine and alcohol during pregnancy [8, 9, 13]. Furthermore, infections, particularly those occurring during pregnancy, are suspected to contribute to the development of childhood cancer [14–16], as the fetus is especially vulnerable due to significant physiological changes and rapid fetal cell division [17]. In addition, pregnant women are more susceptible to specific infections, such as urinary tract infections, due to various physiological changes [18, 19]. These infections often necessitate medication use, raising concerns about the potential impact of prenatal exposure to medications on childhood cancer risk [20]. Commonly prescribed medications in pregnancy include antibiotics, analgesics, antiemetics, hormones like progesterone as well as vitamin supplements, especially folic acid preparations [21–23].

Up to 90% of pregnant women are prescribed medication [24–26], yet its potential impact on childhood cancer risk remains unclear. Existing observational studies report inconsistent findings, and no comprehensive meta-analysis has synthesized this evidence. To fill this gap, we conducted a systematic review and meta-analysis to evaluate the associations between maternal medication use during pregnancy and the risk of various childhood cancers.

## Material and methods

This systematic review and meta-analysis was conducted in accordance with the Preferred Reporting Items for Systematic reviews and Meta-Analyses (PRISMA) [27] and Meta-analysis Of Observational Studies in Epidemiology (MOOSE) guidelines [28]. The objective was to consolidate all available risk estimates regarding the association between prenatal medication exposure and the risk of childhood cancer. This review is registered in the PROSPERO database (CRD42024520521).

### Search strategy

**Fig. 1:**
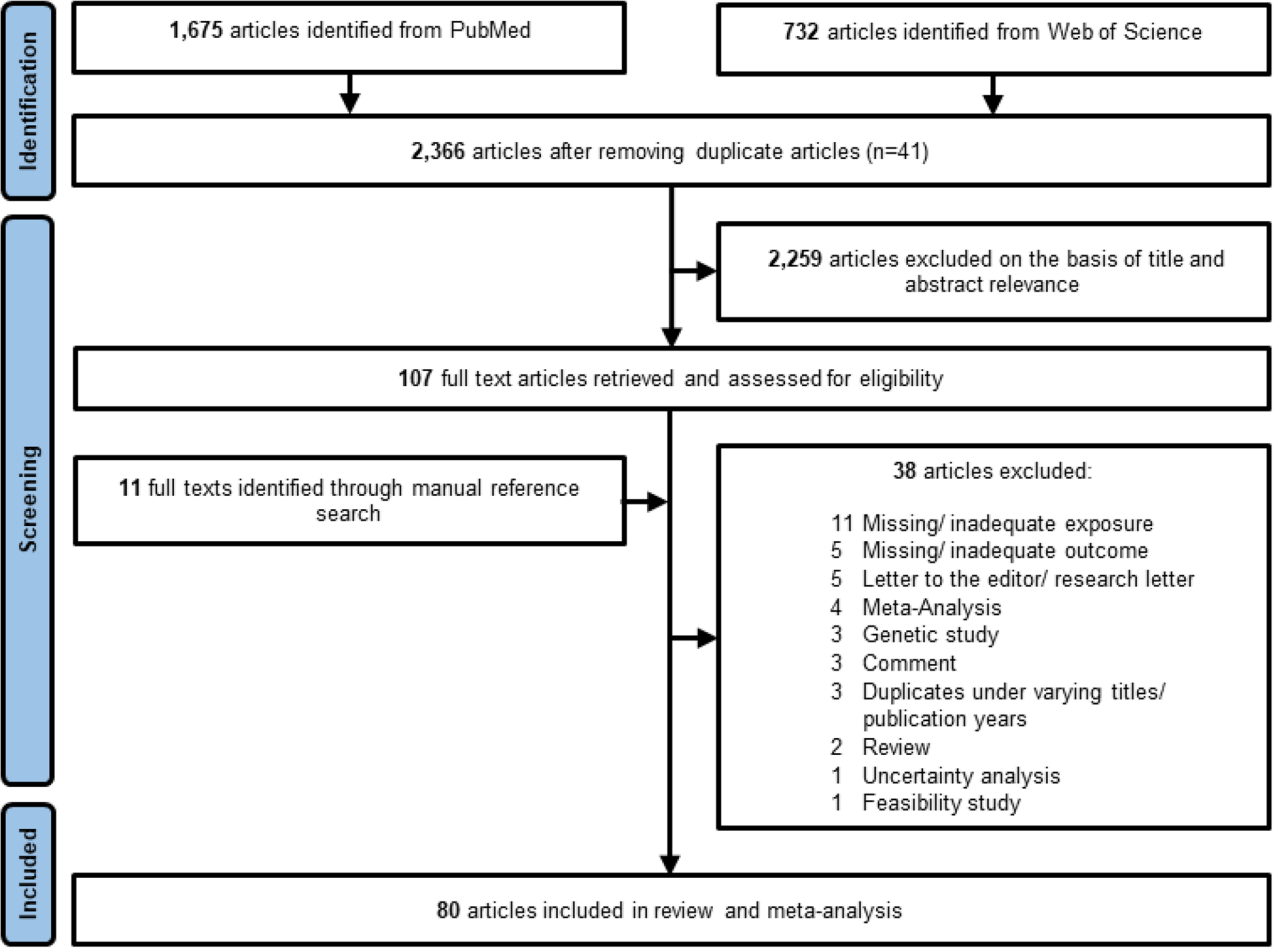
Flow diagram for the selection of studies included in the systematic review and meta-analysis

We conducted a comprehensive literature search using PubMed and Web of Science to identify all relevant studies published up to 15th July 2025 that investigated the association between prenatal exposure to medications or specific pharmaceutical agents and various childhood cancer sites. The search was conducted using English-language keywords and subject headings related to the exposure, outcome, and population of interest. The search strategy was designed to maximize sensitivity and specificity, with combinations of keywords and subject headings tailored to capture relevant studies. Additionally, reference lists of included studies and relevant abstracts were manually searched to identify studies that might have been missed in the automated search. A detailed description of the search strategy is provided in Supplementary Table 1, and the inclusion and exclusion criteria are defined and summarized in Fig. 1.

### Study selection

Duplicate references were removed using EndNote reference management software. Titles and abstracts were screened by two independent researchers (AL and LKB), followed by a full-text review of selected studies. Epidemiological studies that investigated intake of medication or vitamin- and mineral supplements in pharmaceutical form during pregnancy and its relation to childhood cancer were included. Cases needed to be diagnosed with any type of cancer before the age of 20 years, while the reference group was deemed appropriate if the controls were either cancer-free or had a cancer with unrelated etiology to the one being investigated in the study. Regarding the timepoint of exposure, studies that combined analyses of pre-pregnancy and pregnancy medication use were included, whereas studies exclusively focusing on the pre-pregnancy period were excluded. Studies investigating diethylstilbestrol as an exposure were excluded due to its established association with an increased risk of vaginal clear cell adenocarcinoma in female offspring [29]. Only studies that provided quantitative risk estimates, such as odds ratios (OR), relative risks (RR), hazard ratios (HR), standardized incidence ratios (SIR), or incidence rate ratios (IRR) with accompanying confidence intervals (CI), variance, standard error (SE), or standard deviation (SD), were included in the meta-analyses. As different quantitative risk estimates for rare outcomes, including childhood cancer, lead to comparable results, these were pooled for the analyses [30].

### Data extraction

Data were extracted from all identified studies; those included in meta-analyses are shown in Table 1. Extracted characteristics include name of first author, publication year, study region, age range, study design, cancer site, number of cases and controls, investigated type of medication, assessment of exposure and outcome, brief summary of results, statistical analysis, risk estimates, reference group (e.g. population-based or hospital-based), and general conclusion. In cases of potential overlap in study populations, the study with the larger sample size was included. If the studies addressed different research questions (different exposure and/or outcome), both were retained. In addition, when pooled studies were available, we prioritized inclusion of all original studies over the pooled analyses.

**Table 1:** Study characteristics of studies included in meta-analysis

First author, Year	Location	Study years	Study design	Cases/controls	Age range	Cancer sites	Medication	Exposure	Outcome				Conclusion	Quality
Gold, 1978 [31]	US	1965-1975	CC	127/pop.: 73 hos.: 78	< 20	Intracranial and spinal cord tumors	Barbiturates	Self-reported	Death certificates, registries, and medical records, pathological confirmation				Maternal use of barbiturates was more frequent in case mothers than in mothers of controls	18,8
van Steensel-Moll, 1985 [32]	Netherlands	1973-1980	CC	519/507	< 15	ALL	Hormones	Self-reported	Registry data, pathological confirmation				Increased risk of ALL after prenatal exposure to drugs to maintain pregnancy and sedatives/sleeping pills	24,0
McKinney, 1985 [33]	England	1980-1983	CC	555/1110	< 15	Leukemias/lymphomas, other solid tumors	Antiemetics	Medical records and self-reported	Medical records and registry data				Significantly decreased risk for reported Debendox intake in pregnancy for all cancer sites except leukemia /lymphomas, but did not remain within medically recorded data. Debendox use in pregnancy reported in medical records for 1–2 months showed a significant increase of risk for all investigated cancer sites combined	20,8
Kramer, 1987 [34]	US	1970-1979	CC	104/101		Neuroblastoma	Antiemetics, diuretics, neurally active drugs, sex hormones	Self-reported	Registry data, pathological confirmation				Increased risk of neuroblastoma after prenatal exposure to neurally active drugs, sex hormone exposure 3 months prior to pregnancy or during pregnancy and diuretics	22,4
Buckley, 1989 [35]	US	1980-1983	CC	75/75	< 15	Hepatoblastoma	Oral contraceptives, antiemetics, sedatives/tranquilizer, diet pills, vitamins or iron, diuretics, antibiotics, cold or cough remedy, antihistamines, hormones, other medication	Self-reported	Medical records, pathologically confirmed				No association between hepatoblastoma and prenatal exposure to investigated types of medication	19,2
Robison, 1989 [36]	US, Canada	1980-1984	CC	204/204	< 18	acute non-lymphoblastic leukemia	Hormones, antiemetics, sedatives, vitamin supplements, diuretics, antihypertensives, antibiotics, cold or cough remedies, antihistamines	Self-reported	Medical records, pathological confirmation				No association, borderline-significant result for use of antiemetics in pregnancy and ANLL in offspring	22,6
Goldhaber, 1990 [37]	US	1960-1983	CC	86/172	0–19	Intracranial and spinal cord tumors	Barbiturates	Medical records	Medical records and registry data, pathological confirmation				No association between prenatal exposure to barbiturates and intracranial and spinal cord tumors	23,6
Kuijten, 1990 [38]	US	1980-1986	CC	163/163	< 15	Astrocytoma	Neurally acitve drugs, antiemetics, diuretics, antihistamines	Self-reported	Registry data				Maternal intake of antinausea medications increased the risk of childhood astrocytoma, no association for other medication	20,0
Schwartzbaum, 1992 [39]	US	1979-1986	CC	101/690	< 9	Neuroblastoma	Diuretics, antihypertensives, tranquilizer, analgesics, antiemetics, Sex hormones	Self-reported	Medical records, pathologically confirmed				Analyses revealed increased risk estimates for prenatal exposure to tranquilizer, analgesics, diuretics and risk of neuroblastoma	22,4
Cordier, 1994 [40]	France	1985-1987	CC	75/113	0–15	Intracranial tumors	Antihistamines and vitamin supplements	Self-reported	Medical records				Decreased risk of intracranial tumors after prenatal exposure to vitamin supplements	19,6
McCredie, 1994 [41]	Australia	1988-1990	CC	82/164	0–14	CNS tumors	Drugs to prevent miscarriage, diuretics, tranquilizer, analgesics, antihistamines, cold or cough remedies	Self-reported	Registry data, pathological confirmation				No associations between use of investigated medication types in pregnancy and CNS tumors in children	23,8
Shu, 1995 [42]	US, Canada	1982-1989	CC	105/639	< 15	Malignant germ cell tumors	Oral contraceptives, antiemetics, vitamin supplements, analgesics, antibiotics and antihypertensives	Self-reported	Medical records, pathological confirmation				No association between malignant germ cell tumors in children and use of investigated medication types in pregnancy	23,4
Michalek, 1996 [43]	US	1976-1987	CC	183/372	0–14	Neuroblastoma	Vitamin supplements and hormones	Self-reported	Registry data, medical records, pathological confirmation				Vitamin supplement use in pregnancy was associated with a decreased risk of neuroblastoma, sex hormone use with an increased risk	23,4
Preston-Martin, 1998 [44]	US, France, Italy, Spain, Israel, Canada, Australia	1976–1994	CC	1051/1919	≤ 19	Primary tumor of the brain, cranial nerves or cranial meninge	Vitamin supplements	Self-reported	Medical records, pathologically confirmed				Maternal vitamin supplementation for 2 trimesters was associated with a decreased risk of childhood brain tumors, greatest risk reduction: children diagnosed under 5 years of age with maternal supplement use over all 3 trimesters	24,8
Thapa, 1998 [45]	US	1975-1992	CO	175/328,846	< 5	Acute leukemia, CNS tumors, neuroblastoma, other cancers	Antibiotics (Metronidazole)	Medical records	Medical records, pathologically/radiologically confirmed				No association between maternal antibiotic use in pregnancy and risk of investigated childhood cancer sites	29,8
McKinney, 1999 [46]	Scotland	1991-1994	CC	390/716	0–14	Leukemia, lymphoma, CNS-tumors, other solid tumors	Antacids, hypnotics and anxiolytics, analgesics, antibiotics, topical anti-fungals, iron supplements, antiemetics	Medical records and self-reported	Registry data, pathologically confirmed				Increased risk after prenatal exposure to antibiotics and risk of solid tumors, inverse association between maternal intake of topical antifungals and leukemias in offspring	26,2
Schüz, 2001 [47]	Germany	1988-1994	CC	183/1785	< 8	Neuroblastoma	Hormones	Self-reported	Registry data				Maternal use of oral contraceptives or other sex hormones during pregnancy was associated with an increased risk of neuroblastoma in offspring (particularly with male offspring and low-stage neuroblastoma)	23,2
Thompson, 2001 [48]	Australia	1984-1992	CC	83/166	0–14	ALL	Vitamin supplements	Self-reported		Medical records	Decreased risk of ALL after prenatal exposure to iron/folate supplementation, especially for folic acid			19,2
Olshan, 2002 [49]	US, Canada	1992-1994	CC	538/504	< 19	Neuroblastoma	Vitamin supplements	Self-reported		Medical records, pathologically confirmed	Daily vitamin and mineral use in pregnancy was associated with a decreased risk of neuroblastoma in offspring (30–40%)			24,4
Shu, 2002 [50]	US	1989-1993	CC	1842/1986	< 15	ALL	Hormones	Self-reported		Medical records, pathological confirmation	Maternal use of oral contraceptives in pregnancy was associated with an increased risk of ALL in offspring, especially for children under the age of 2 years			25,6
Wen, 2002 [51]	US, Canada	1989-1993	CC	1842/1986	< 15	ALL	Hormones, antiemetics, sedatives/tranquilizer, vitamin and iron supplements, diuretics, antihypertensives, antibiotics, cold or cough remedies, antihistamines, laxatives or antidiarrhea agents, immunosuppressants, antiinflammatory drugs, analgesics	Self-reported		Medical records, pathologically confirmed	Maternal use of vitamins and iron supplements during pregnancy was associated with a decreased risk of ALL in offspring, however anemia as underlying disease was not associated with ALL risk			27,4
Cook, 2004 [52]	US, Canada	1992-1994	CC	504/504	≤ 19	Neuroblastoma	Antihistamines, antibiotics, sympathomimetic agents, analgesics, antitussives and expectorants, hormonal agents, antihypertensives, opiod agonists	Self-reported		Medical records, pathologically confirmed	Maternal intake of Codeine was associated with an increased risk of neuroblastoma			25,6
Shaw, 2004 [53]	Canada	1980-2000	CC	789/789	< 15	ALL	Hormones, antiemetics, CNS depressants, antiepileptics, hormones, immunosuppressants, antibiotics, antiinflammatories, analgesics, antihistamines, anti-asthmatics, antiacids, antidiabetics, vitamin supplements	Self-reported		Medical records	ALL risk in children was associated with maternal use of any of investigated medication and with CNS-depressants in pregnancy			22,6
Bunin, 2006 [54]	US	1991-1997	CC	315/315	< 6	Brain tumors	Vitamin supplements	Self-reported		Medical records, pathologically confirmed	Calcium supplementation during third trimester: tendency to a decreased risk of MB/PNET			26,4
Pombo-de-Oliveira, 2006 [55]	Brazil	1999-2005	CC	202/440	0–21 month	Infant leukemia	Analgesics, antibiotics, folic acid and vitamin supplements, antiemetics, antifungicids, abortive drugs, hormones, herbal medication	Self-reported		Medical records, Registry data, pathological confirmation	Maternal use of hormones in pregnancy was associated with an increased risk of infant leukemia			22,8
Shankar, 2006 [56]	US	1996-2002	CC	278/423	< 15	Malignant germ cell tumors	Hormones	Medical records and self-reported		Medical records, pathologically confirmed	No association between prenatal exposure to hormones and risk of malignant germ cell tumors			26,6
Dockerty, 2007 [57]	New Zealand	1990–1993	CC	97/303	0–14	Acute lymphoblastic leukemia	Vitamin and mineral supplements	Self-reported		Registry data	No association between vitamin and mineral supplementation in pregnancy and ALL in children			19,8
Kwan, 2007 [16]	US	1995-2002	CC	365/365	< 15	Acute leukemia	Oral contraceptives, antiemetics, iron supplements, antibiotics, herbal medication	Self-reported		Medical records, Registry data	No association for iron supplement use in peripregnancy, but decrease in risk of AL for consideration of overall use in preconception, during pregnancy and while breastfeeding			21,6
Schüz, 2007 [58]	Germany	1992-1997	CC	1867/2057	≤ 14	Acute leukemia, NHL, CNS tumor, neuroblastoma, Wilms' tumor, bone tumor and soft tissue sarcoma	vitamin, folate or iron supplements, antiemetics, diuretics or other antihypertensives, tranquillizers or sleeping pills, analgesics, cold medications, psychotropics, antiepileptics, and diet pills	Self-reported		Registry data	Maternal use of vitamin, folate or iron supplementation was associated with a reduced risk of NHL and Wilms tumors in children. An increased risk of neuroblastoma was associated with diuretics/antihypertensives, but also with vitamin, folate or iron supplementation in pregnancy			22,2
Benhammou, 2008 [59]	France	1984-2007	CO	10/12,074		ALL, retinoblastoma, pineoblastoma, glioma	Antiretroviral HIV-medication	Medical records, selfreported		Self-reported, medical records, registry data	Antiretroviral HIV-medication was not associated with childhood cancer, but didanosine-lamivudine carries higher risk than zidovudine monotherapy			26,8
Johnson, 2009 [60]	US, Canada	1993-2001	CC	278/423	< 15	Germ cell cancer	Vitamin supplements	Self-reported		Medical records, pathologically confirmed	No association between prenatal exposure to vitamin supplements and malignant germ cell tumors			23,8
Kaatsch, 2010 [61]	Germany	1992-1994	CC	1867/2057	0–14	Acute leukemia, NHL, Burkitt lymphoma, CNS-tumors, neuroblastoma, Wilm's tumor, bone tumor, soft issue sarcoma	Antibiotics	Self-reported		Registry data	Increased risk of ALL, AML and Burkitt lymphoma after prenatal exposure to antibiotics			22,2
Linabery, 2010 [62]	US, Canada	1996-2006	CC	443/324	< 12 month	Infant leukemia	Vitamin supplements	Self-reported		Medical records, pathologically confirmed	No association between vitamin supplement use in pregnancy and infant leukemia			23,4
Milne, 2010 [63]	Australia	2003-2007	CC	416/1361	< 15	ALL	Vitamin supplements	Self-reported		Medical records, Registry data	Maternal folic acid supplementation was not associated with ALL in offspring, weak evidence for inverse association between iron supplements > 10 µg in first trimester and ALL			22,6
Ognjanovic, 2011 [64]	US, Canada	1996-2006	CC	441/323	0–12 month	Infant leukemia	Analgesics	Self-reported		Medical records, pathologically confirmed	No association between maternal analgesics use in pregnancy and infant leukemia			23,4
Ortega-García, 2010 [65]	Spain	2004-2006	CC	67/155	< 15	(Central) nervous system tumors	Folic acid and vitamin supplements	Self-reported		Registry data	Supplementation before the 21 st and 36th days of gestation was associated with a decreased risk of nervous system tumors			19,8
Stålberg, 2010 [66]	Sweden	1975-1984	CC	512/525	≤ 15	Brain tumors	Alimentary tract medication, vitamin supplements, folic acid supplements, antihypertensives, diuretics, anti-infectives/antibiotics, analgesics, antiasthmatics, antiemetics, antihistamines	Medical records and registry data		Registry data, pathological confirmation	Prenatal exposure to antihypertensives was associated with an increased risk of childhood brain tumors			28,0
Amigou, 2012 [67]	France	2003-2004	CC	764/1681	< 15	Acute leukemia	Folic acid and vitamin supplements	Self-reported		Registry data	Decreased risk of AL after prenatal exposure to folic acid supplements			26,6
Milne, 2012 [68]	Australia	2005-2010	CC	327/867	0–14	Brain tumors	Vitamin supplements	Self-reported		Medical records	No association, but assumption of protective effect of folic acid supplementation in pregnancy and risk of childhood brain tumors			24,8
Ajrouche, 2014 [69]	France	2010-2011	CC	747/1421	< 15	Acute leukemia	Folic acid supplements and hormones	Self-reported		Registry data	Increased risk of AL after prenatal exposure to oral contraceptives of 3rd generation			26,6
Bonaventure, 2015 [70]	UK	1991-1996	CC	1598/2524	< 15	Leukemia, lymphoma, CNS tumors, sarcomas, other cancers	Iron supplements, antiinfectives/antibiotics, Alimentary tract medication, dermatologicals, hormones, nervous system medication, analgesics, respiratory medication	Medical records, selfreported		Medical records	Maternal use of iron supplements was positively associated with ALL, medulloblastoma, and Wilms tumor; systemic antibacterials with rhabdomyosarcoma; penicillins with AML; nervous system drugs with HL and neuroblastoma; alimentary/metabolic drugs with Ewing sarcoma; dermatologicals with rhabdomyosarcoma. Maternal respiratory medication use was associated with a reduced risk of astrocytoma			22,6
Couto, 2015 [71]	Brazil	1999-2007	CC	231/411	0–23 month	Early age leukemia	Analgesics	Self-reported		Medical records, pathologically confirmed	Maternal Acetaminophen use in pregnancy showed a decreased risk for ALL in offspring, dipyrone use in pregnancy was associated with an increased risk of leukemia			24,4
Gradel., 2015 [72]	Denmark	1995-2008	CC	360/3509	< 15	Acute Leukemia	Antibiotics	Registry data		Registry data	No association between prenatal exposure to antibiotics and AL			25,4
Ivy, 2015 [73]	US	1995-2008	CO	4/2326	< 19	Hodgkin nodular sclerosis, AML, hepatocellular carcinoma, pleuropulmonary blastoma	Antiretroviral HIV-medication	Registry data		Registry data	No association between prenatal exposure to antiretroviral HIV-medication and childhood cancer			23,4
Momen, 2015 [74]	Sweden Denmark	1995–2007 (D), 2005–2009 (S)	CO	1479/1,447,238		Acute leukemia, cancers of the central and sympathetic nervous system, renal tumors	Antibiotics	Registry data		Registry data	General antibacterial use during pregnancy was not associated with increased childhood cancer risk, but significant increased risk results for specific cancer sites			30,6
Hleyhel, 2016 [75]	France	1990-2014	CO	21/15,163		ALL, lymphoma, CNS tumors, pineoblastoma, retinoblastoma rhabdomyo-sarcoma	Antiretroviral HIV-medication (Didanosine)	Medical records		Registry data and self-reported	Increased risk of childhood cancer after prenatal exposure to didanosine in first trimester			29,8
Mortensen, 2016 [76]	Norway	1999-2010	CO	799/687,406		Acute leukemia, lymphoma, CNS tumors, neuroblastoma, Wilms’ tumour, soft tissue tumors	Folic acid and vitamin supplements	Registry data		Registry data	No association between folic acid and/or vitamin supplement use in pregnancy and investigated childhood cancer sites			29,4
Singer, 2016 [77]	US	1995-2008	CC	784/1076	0–14	ALL, AML	Vitamin supplements	Self-reported		Medical records, pathological confirmation	The analyses stratified on Hispanic mothers revealed a significant risk reduction of ALL in offspring prenatally exposed to vitamin supplements			24,6
Vienneau, 2016 [78]	Switzer-land, Sweden, Norway, Denmark	2004–2008	CC	352/646	7–19 years	CNS tumors	Vitamin supplements	Registry data and selfreported		Registry data, Medical records, pathologically confirmed or with diagnostic imaging	Borderline-significant result for decreased risk of CNS tumors after prenatal exposure to vitamin supplements			26,4
Hargreave, 2018 [79]	Denmark	1996-2014	CO	606/1,185,157		Acute leukemia, ALL, non-lymphoid leukemia	Hormonal contraceptives	Registry data		Registry data		Maternal use of hormonal contraceptives was associated with increased risk for non-lymphoid leukemia in children		31,4
Momen, 2018 [80]	Denmark	1998-2012	CO	1298/915,128		Childhood cancer, acute leukemia, central and sympathetic nervous system tumors	Antidepressants	Registry data		Registry data		No association between antidepressant use in pregnancy and investigated childhood cancer sites		34,2
Ye, 2019 [81]	Canada	1996-2013	CO	361/262,116	< 20	Childhood cancer, acute leukemia	Antibiotics	Insurance data, pharmacy claims		Registry data, pathologically confirmed		Antibiotics use during first trimester was associated with an increased risk of childhood cancer		31,4
Bauer, 2020 [82]	France	2010-2011	CC	117/1100	< 11	Wilms tumor/nephroblastoma	Folic acid supplements	Self-reported		Registry data		No association between folic acid supplements and Wilms tumors		24,4
Seppälä, 2020 [83]	Finland	1996-2014	CC	2029/10,103	< 20	Childhood cancer	Antidiabetics	Registry data		Registry data		No association between prenatal exposure to antidiabetics and risk of childhood cancer		26,2
Hjorth, 2022 [84]	Finland, Norway, Sweden, Denmark	1997–2013	CO	161/291,397		Acute leukemia	Antibiotics (Nitrofurantoin)	Registry data		Registry data		Prenatal exposure to nitrofurantoin was not substantially associated with childhood leukemia, slightly elevated IRR for analysis on prenatal exposure to Nitrofurantoin in third trimester and any leukemia in offspring		34,6
Vegrim, 2022 [85]	Norway, Sweden, Denmark	1997–2017	CO	18 with epilepsy; 69 without/3,379,171	< 20	Childhood cancer	Folic acid supplements	Registry data		Registry data, pathological confirmation		High-dose folic acid in pregnancy/mothers with epilepsy: increased risk of childhood cancer, high-dose folic acid in pregnancy/mothers without epilepsy: no association with childhood cancer		36,4
Yan, 2022 [86]	China	2009-2016	CC	46/45	< 18	Nasopharyngeal carcinoma	Vitamin supplements	Self-reported		Medical records, pathologically confirmed		Maternal use of folic acid and/or multivitamins was associated with a reduced risk of nasopharyngeal carcinoma in offspring		24,4
Avagyan, 2023 [87]	Armenia	2017-2020	CC	117/117	≤ 14	Childhood cancer	Folic acid supplements	Self-reported		Registry data		Decreased risk of childhood cancer after prenatal exposure to folic acid supplements		18,2
Cheng, 2023 [88]	Canada	1997-2003	CC	280/pop.:490, hos.: 429	0–15	Brain tumors	Antihistamines, Analgesics, Antidepressants/anxiety medication, immunosuppressants	Self-reported		Medical records, pathologically confirmed		Prenatal exposure to immunosuppressants was associated with an increased risk of glial tumors		27,4
Jung, 2023 [89]	US, Canada	1998-2012	CC	168/145		Sporadic unilateral retinoblastoma	Vitamin supplements	Self-reported		Medical records, pathological confirmation		Higher intake of vitamins A and D during pregnancy might be a protective factor for sporadic unilateral RB in children		26,4
Orimoloye, 2023 [90]	Denmark	1995-2014	CC	2521/160,500	< 20	Childhood cancer, ALL, AML, lymphomas, CNS tumors, intracranial tumors, neuroblastoma	Antiemetics	Registry data		Registry data		No association between antiemetic use in pregnancy and investigated childhood cancer sites		29,0
Qureshi, 2023 [91]	Denmark	1995-2014	CC	6420/160,485	≤ 19	AML, ALL, lymphoma, CNS tumors, Gliomas, Neuroblastoma, Nephroblastoma, Malignant bone tumors, osteosarcoma, germ cell tumor	Vitamin supplements for maternal anemia	Registry data		Registry data		Mothers prescribed supplements for B12 and folate deficiency anemia had an increased risk for cancer in offspring		29,0
Sirirungreung, 2023 [92]	Denmark	1996-2016	CC	1749/43,841	0–19	Acute leukemia, CNS tumors, NHL; germ cell tumors, neuroblastoma, Wilms-tumor, retinoblastoma	Nitrosatable antibiotics and nitrosatable drugs	Registry data		Registry data		Positive association between prenatal exposure to nitrosatable drugs and CNS-tumors/neuroblastoma and ALL		26,4
Askins, 2024 [93]	Denmark	1995-2014	CC	2521/63,025		Childhood cancer, CNS tumors, ALL	Antihypertensives and diuretics	Registry data		Registry data		Increased risk of ALL in children prenatally exposed to diuretics		28,0
Orimoloye, 2024 [94]	Taiwan	2004-2015	CO	2780/2,291,512	< 13	Childhood cancer	Diuretics and any medications other than diuretics	Accounting data		Accounting data		Weak evidence of an increased risk of childhood cancer after prenatal exposure to diuretics and antihypertensives others than diuretics		34,0
Platamone, 2024 [95]	Denmark	1995-2016	CC	2521/63,014	0–19	ALL, CNS tumors and childhood cancer	Anticonvulsants	Registry data		Registry data, pathological confirmation		Maternal anticonvulsant use before or during the index pregnancy was related to CNS tumors in offspring		29,0
Sirirungreung, 2024 [96]	Taiwan	2004-2015	CO	1241/2,267,186		ALL, CNS tumors, hepatoblastoma, medulloblastoma	Antibiotics	Accounting data		Accounting data		Increased risk of ALL, hepatoblastoma and medulloblastoma after prenatal exposure to antibiotics		31,4
Gudnadottir, 2025 [97]	Sweden	2006-2016	CO	1091/722372		Childhood cancer	Antibiotics and proton pump inhibitors	Registry data		Registry data		No association between prenatal exposure to antibiotics or proton pump inhibitors and childhood cancer		28,4

Study quality was assessed using the self-developed scoring tool with a maximum of 45 points

*Abbreviations*: *CC* Case–control, *CO* Cohort, *pop.* Population, *hos.* Hospital, *ALL* Acute lymphoblastic leukemia, *AML* Acute myeloid leukemia, *CNS* Central nervous system, *NHL* Non-Hodgkin lymphoma

### Quality assessment

Quality of studies was assessed using a scoring system adapted from the Newcastle–Ottawa Scale (NOS) and the Risk Of Bias in Non-randomized Studies Tools of Exposure (ROBINS-E) and of Intervention (ROBINS-I). The scoring tool with a maximum score of 45 points is an extension of the NOS that incorporates risk of bias assessment criteria from ROBINS. The tool is described elsewhere [98]. Briefly, it contains eight major categories, including study design (up to six points), sample size (up to six points), assessment of outcome (up to six points) and exposure (up to six points), controlling for potential confounder (up to six points), statistical methods applied (up to six points), general methods applied in the study (up to six points) and reported basic characteristics (up to three points). Two independent reviewers (AL and LM) performed the quality assessment of the studies and, if necessary, discussed and clarified discordances with a third reviewer (LKB). A detailed overview of the quality assessment is available in Supplementary Table 2.

### Data analysis

Meta-analyses were performed to generate pooled estimates (ES) with 95% CI using random effects models for hypotheses with at least three available studies on same exposure and outcome [99]. The *I*^*2*^ statistic was calculated to quantify between-study heterogeneity. *I*^*2*^ values of 50% or less were classified as a low indication of heterogeneity, between 50 and 75% as an indication of moderate heterogeneity and above 75% as significant heterogeneity [100]. Statistical significance of *I*^*2*^ was analyzed with the Q statistic [P value for heterogeneity (P)]. Sensitivity analyses were conducted in order to investigate whether exclusion of single studies might decrease heterogeneity between studies (Supplementary Table 3) [101]. Subgroup analysis on different study characteristics were performed to explore potential sources of heterogeneity, on factors such as publication date (< 2010: early study, ≥ 2010 late study, Supplementary Table 4), region (America (North and South), Europe, Australia/New Zealand, Asia, Supplementary Table 5), study design (case–control study, cohort study, Supplementary Table 6); exposure assessment (low: self-reported, high: medical records, registry, trial data, Supplementary Table 7), level of adjustment (low: basic factors e.g. sex, region, SES, (maternal) age, birth order, educational level or no adjustment; high: maternal exposure to medication, pesticides, ionizing radiation, alcohol or nicotine, and diseases of mother or child, Supplementary Table 8), quality-score below and above the fourth quintile (low: quality score < 28.5, high: quality score ≥ 28.5, Supplementary Table 9) and outcome assessment based on pathological confirmation or registry (yes or no, Supplementary Table 10). Analyses were initially conducted on prenatal exposure to various medication types and overall childhood cancer risk (Supplementary Figs. 1–13). When these analyses indicated significant results or trends toward a significant result of an increase or decrease in childhood cancer risk, we performed specific analyses based on specific cancer sites. Publication bias was assessed using funnel plots and Egger’s asymmetry test [102] if a sufficient number of studies (n > 9) were available for one meta-analysis (Supplementary Figs. 14–16). All calculations were performed using Stata version 18 (StataCorp LP, College Station, TX, USA) [103].

## Results

### Literature search

Our literature search retrieved 2,366 articles in *PubMed* and *Web of Science*, of which 2,259 were excluded based on title and abstract relevance. The remaining 107 potential full texts were complemented by 11 additional articles from manual reference search (Fig. 1). After full-text screening, 80 studies were included in the systematic review, containing 1,215 reported risk estimates for different types of medication and childhood cancer sites. Based on the available risk estimates, we included 68 studies, encompassing 14,396,922 study participants, in meta-analyses on prenatal exposure to 13 different medication types and risk of different childhood cancers. Twelve studies were either excluded based on an overlap in study populations [104–113], or the lack of medication-specific risk estimates [114] or due to absence of any risk estimates [115].

### Study characteristics and quality

A summary of the study characteristics of studies included in the meta-analyses is shown in Table 1. The studies, published from 1978 to 2025, involved between 91 and 3,379,171 participants. In total, 45% of studies were conducted in Europe, 41% in America (North and South), 8% in Australia and New Zealand and 6% in Asia. Overall, 21% of the studies were cohort studies [45, 59, 73–76, 79–81, 84, 85, 94, 96, 97] and 79% case–control studies. Among the case–control studies, 79% were population-based [16, 32, 34–36, 38, 40–44, 46–54, 56–58, 60–64, 66–70, 77, 78, 82, 83, 89–93, 95], 13% hospital-based [37, 39, 55, 65, 71, 86, 87] and 6% combined hospital- and population-based [31, 33, 88]; one study employed a nested case–control design [72]. The outcome assessment varied between registry data (46%), medical records (37%) and the use of both (12%). One cohort study [59] used self-reports, medical records and registry data, another [75] combined registry data and self-reports, and two studies [94, 96] relied on accounting data. Prenatal exposure to medication was mainly (57%) identified via parental self-reports [16, 31, 32, 34–36, 38–44, 47, 48, 50–55, 57, 58, 60–65, 67–69, 71, 77, 82, 86–89]. Registry data were used in 22% of studies [72–74, 76, 79, 80, 83–85, 90–93, 95, 97], 7% utilized medical records and self-reports for exposure assessment [33, 46, 56, 59, 70], and fewer than 5% used medical records [37, 45, 75] or combinations of medical records and registry data [66], self-reports and registry [78] or insurance data/pharmacy claims [81, 94, 96]. In terms of adjustment, 62% of studies accounted only for baseline confounder (e.g., age, gender, region and socioeconomic factors), 29% additionally adjusted for other factors (e.g., maternal medication usage before pregnancy, diseases of mother or child, maternal exposure to nicotine or alcohol) and 9% did not adjust for any potential confounders. Quality assessment of included studies yielded an average mean score of 25.2 (range 11.0 to 36.4) out of 45 points.

### Results of the meta-analyses

We conducted 13 analyses on prenatal exposure to different types of medication and risk of overall childhood cancer and 54 specific analyses covering ten various types of medication and 11 childhood cancer sites.

**Fig. 2:**
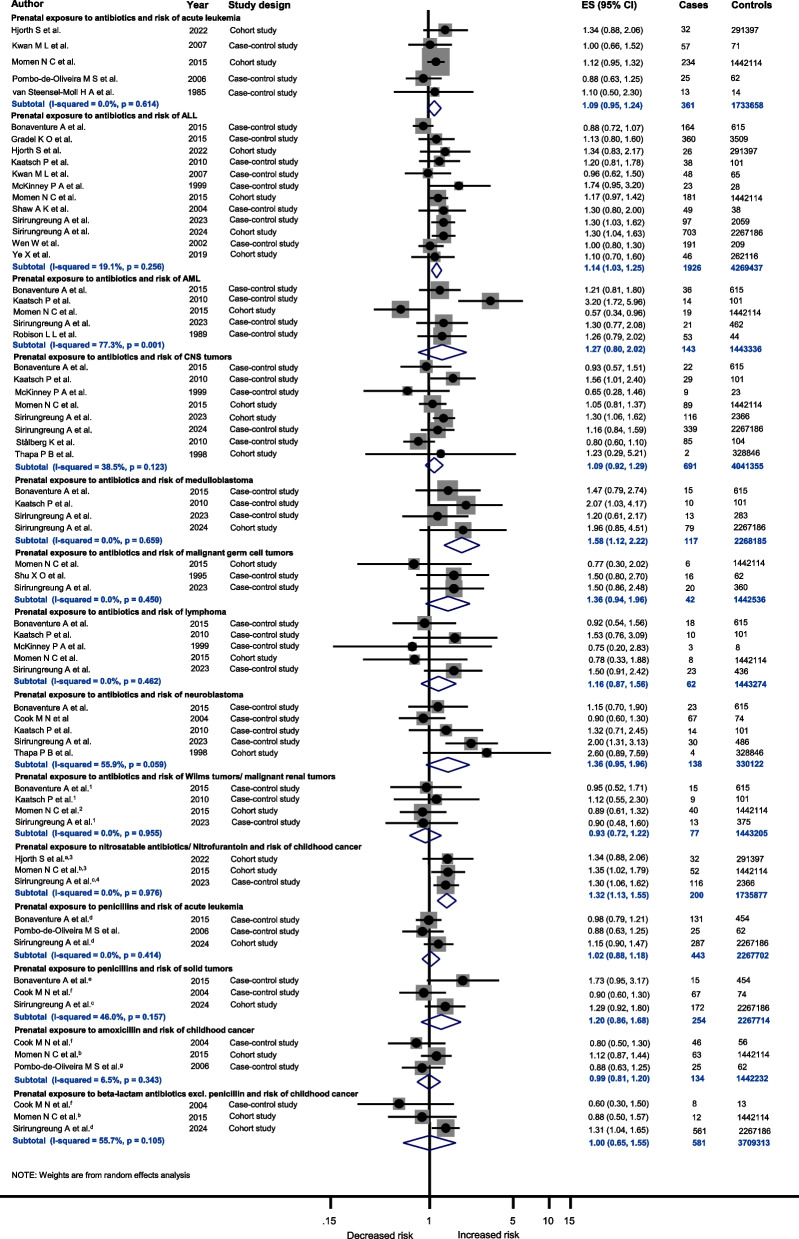
Prenatal exposure to antibiotics and the risk of different childhood cancer sites. Abbreviations: ES, estimate; ALL, acute lymphoblastic leukemia; AML, acute myeloid leukemia; CNS tumors, central nervous system tumors; investigated childhood cancer site: ^a^ any leukemia, ^b^ leukemias, cancers of the central and sympathetic nervous system, renal tumors, ^c^ CNS tumors, ^d^ ALL, ^e^ non-Hodgkin lymphoma, ^f^ neuroblastoma, ^g^ infant acute leukemia; ^1^ Wilms tumor, ^2^ malignant renal tumor ^3^ nitrofurantoin, ^4^ nitrosatable antibiotics

Associations between maternal intake of antibiotics and risk of different childhood cancer sites are presented in Fig. 2. The pooled analyses on prenatal exposure to antibiotics of any kind revealed an increased risk of medulloblastoma (ES = 1.58 (95% CI 1.12; 2.22), *I*^2^ = 0.0%, *P =* 0.659, number of studies (N) = 4) in children. Similar results were found for acute lymphoblastic leukemia (ALL) (ES = 1.14 (1.03; 1.25), *I*^2^ = 19.1%, *P =* 0.256, *N =* 12), with stratification by trimester showing no association (Supplementary Fig. 17). The positive association observed in the ALL analysis remained consistent even after restricting the analysis to cohort studies as the selected study design (OR = 1.22 (1.07; 1.39),* I*^2^ = 0.0%, *P =* 0.830, *N =* 4, Supplementary Table 6), high quality studies (OR = 1.22 (1.07; 1.39), *I*^2^ = 0.0%, *P =* 0.830, *N =* 4, Supplementary Table 9), and studies with exposure assessment based on medical records and registries (OR = 1.17 (1.02; 1.33),* I*^2^ = 39.3%, *P =* 0.117, *N =* 8, Supplementary Table 10), supporting the observed increased risk.

In addition, maternal intake of nitrosatable antibiotics, including nitrofurantoin, which is commonly prescribed for urinary tract infections, was associated with an increased risk of childhood cancer in the offspring (ES = 1.32 (1.13; 1.55), *I*^2^ = 0.0%, *P =* 0.976, *N =* 3, Fig. 2).

Maternal intake of analgesics of all kinds revealed an increased risk of childhood neuroblastoma (ES = 1.40 (1.001; 1.95),* I*^2^ = 43.8%, *P =* 0.149, *N =* 4, Supplementary Fig. 18). Prenatal exposure to antiemetics of all types was associated with an increased risk of childhood acute leukemia (AL) (ES = 1.48 (1.08; 2.04),* I*^2^ = 0.0%, *P =* 0.784, *N =* 5, Supplementary Fig. 19). The analyses focusing on children exposed to antihypertensives in prenatal period resulted in risk increases of solid tumors (ES = 1.76 (1.13; 2.74), *I*^2^ = 0.0%, *P =* 0.497, *N =* 5) and ALL (ES = 1.67 (1.11; 2.53),* I*^2^ = 0.0%, *P =* 0.740, *N =* 3, Supplementary Fig. 20).

Associations between maternal hormone use during pregnancy and risk of different childhood cancer types are shown in Supplementary Fig. 21. Maternal intake of hormones has been associated with an increased risk of neuroblastoma (ES = 1.58 (1.11; 2.26),* I*^2^ = 0.0%, *P =* 0.424, *N =* 5) and AL (ES = 1.50 (1.08; 2.08), *I*^2^ = 62.2%, *P =* 0.014, *N =* 7) in children. Results on AL showed significant inter-study heterogeneity, mainly driven by the study from Pombo de Oliveira et al. [55]. By exclusion of this study, heterogeneity turns to zero (ES = 1.27 (1.04; 1.54) I^2^ = 0%), but the association still persists (Supplementary Table 3). Moreover, prenatal exposure to oral contraceptives as specific subtype of hormones was found to increase the risk of ALL in the offspring (ES = 1.28 (1.02; 1.61), *I*^2^ = 0.0%, *P =* 0.698, *N =* 5, Supplementary Fig. 21).

**Fig. 3:**
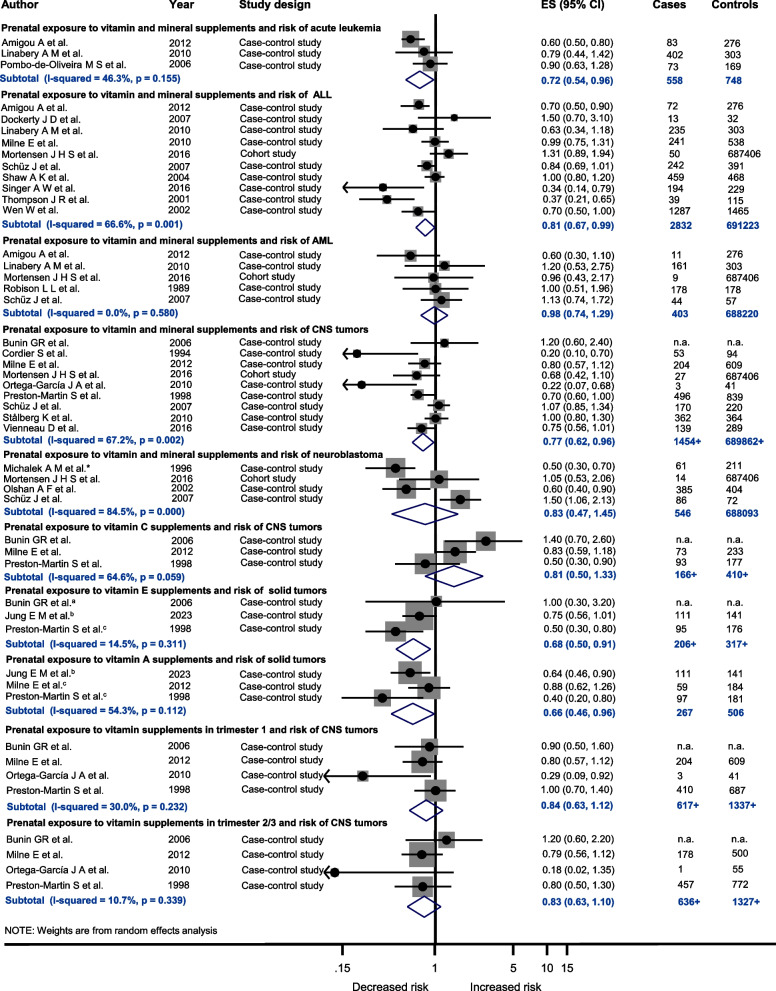
Prenatal exposure to vitamin and mineral supplements and the risk of different childhood cancer sites. Abbreviations: ES, estimate; ALL, acute lymphoblastic leukemia; AML, acute myeloid leukemia; CNS tumors, central nervous system tumors; n.a., not available; investigated childhood cancer site: ^a^ medulloblastoma/primitive neuroectodermal tumors of brain, ^b^ sporadic unilateral retinoblastoma, ^c^ childhood brain tumors; ^*^ calculation of crude estimates

Maternal intake of vitamin and mineral supplements in pregnancy was observed to be associated with a decreased risk of AL (ES = 0.72 (0.54; 0.96), *I*^2^ = 46.3%, *P =* 0.155, *N =* 3), its subtype ALL (ES = 0.81 (0.67; 0.99), *I*^2^ = 66.6%, *P =* 0.001, *N =* 10) and central nervous system (CNS) tumors (ES = 0.77 (0.62; 0.96),* I*^2^ = 67.2%, *P =* 0.002, *N =* 9) in children (Fig. 3). Stratified analyses regarding time point of exposure showed no association (Fig. 3). In addition, specified analyses on maternal intake of vitamins E (ES = 0.68 (0.50; 0.91),* I*^2^ = 14.5%, *P =* 0.311, *N =* 3) and vitamin A (ES = 0.66 (0.46; 0.96),* I*^2^ = 54.3%, *P =* 0.112, *N =* 3) in pregnancy presented a reduced risk of solid tumors in children (Fig. 3).

## Discussion

Our meta-analyses demonstrate an increased risk of ALL in children prenatally exposed to any kind of antibiotics and a reduced risk of acute leukemia, ALL and CNS tumors with maternal supplementation of vitamins and/or minerals during pregnancy.

### Antibiotics

Analyses on maternal intake of antibiotics in pregnancy revealed three pooled associations with increased childhood cancer risk. The results of most of the included studies consistently showed no association. However, one study included in the analysis on medulloblastoma [61] and two studies [92, 96] included in the ALL analysis were associated with an increased risk. Combining all available estimates in the pooled analysis revealed a stable increase in childhood cancer risk, even after exclusion of single studies.

Biologically, an increase in risk can potentially be explained by the fact that most antibiotics are able to pass through the placenta and thus reach the fetus [116]. In addition, antibiotics attack the maternal microbiota of the intestines and the vagina. Current evidence suggests that the maternal microbiome influences the child's immune system even before birth. If the maternal microbiome is now altered by antibiotics or reduced in bacterial numbers, it may influence fetal immunity and microbial colonization, which, potentially, could have an impact on the development of childhood cancer [116–120]. In general, taking antibiotics always goes hand in hand with the presence of an infection, which in turn could also influence the risk of cancer. However, Sirirungreung et al. [96] reported an increased risk of 53% for ALL in children whose mothers took antibiotics during pregnancy without a medically reported infection. A potential explanation for the observed association between antibiotic use without documented infection and hematopoietic malignancies such as ALL could relate to the clinical contexts in which antibiotics are administered prophylactically, such as cases of premature rupture of membranes or preterm birth with uncertain group B streptococcus status [121]. These situations often occur during critical stages of fetal hematopoietic development in the second trimester, when disruptions might plausibly contribute to altered immune or blood cell maturation, thereby influencing later leukemia risk. Moreover, an infection in pregnancy without antibiotic prescription was not associated with childhood ALL, whereas an infection in combination with a prescribed antibiotic in pregnancy revealed a risk increase of 66% [96]. These findings suggest the possibility that both, intake of antibiotics on its own, as well as the combination of an infection and intake of antibiotics in pregnancy might influence the risk of ALL in children. Prenatal exposure to infection and risk of childhood cancer was examined in another paper by our group [122].

### Analgesics

Prenatal exposure to analgesics of all types was associated with increased risk of childhood neuroblastoma, supported by four case–control studies [39, 52, 58, 70], three of them with a small number of cases ranging from 13 to 65 [39, 58, 70]. After omitting single studies in sensitivity analysis, an association was observed only from the exclusion of Cook et al. [52] (Supplementary Table 3). However, it should be emphasized that this case–control study has the highest quality score of the four included studies (25.6 points, range: 22.2–25.6) and had the largest number of cases (*N =* 170). Furthermore, the studies were limited by suboptimal assessment of exposure (i.e., self-reported medication use) [39, 52, 58] and low adjustment for confounders (all included studies adjusted only for baseline confounders).

A recent cohort study from 2024 [123] including 401,462 neonates found an increased risk for infections in both perinatal and childhood periods after prenatal exposure to opioids. This potential immunological effect is supported by the study of Miller et al. [124], who observed reduced levels of neutrophil granulocytes, proinflammatory cytokines and a decreased IL-2 production during in vitro CD4 + T cell proliferation in neonates prenatally exposed to opioids. In addition, maternal paracetamol (acetaminophen) intake during the third trimester has been linked to a trend toward a reduced relative number of hematopoietic stem cells (precursors of immune cells) in cord blood samples, which may suggest an impact on the fetal immune system [125]. This potential effect on the children’s immune system might contribute to the development of other diseases like childhood cancer. Despite the potential immunological effect, the result should be interpreted with caution, considering the aforementioned limitations in the studies.

### Antiemetics

Among the analyses of prenatal exposure to antiemetics, pooled estimates for AL indicated an elevated risk. The main indications for the use of antiemetics during pregnancy are pregnancy-related nausea and vomiting, including its severe form, hyperemesis gravidarum [126]. Hyperemesis gravidarum predominantly occurs during the first trimester, a critical period of rapid fetal cell division. Exposure to medications or other stressors at this stage may increase the likelihood of initiating genetic lesions, which could potentially influence the development of various childhood cancer sites. Besides prenatal use of antiemetics, the case–control study by Orimoloye et al. [90] also investigated the presence of maternal hyperemesis gravidarum in association with cancer in children. Whereas no association was observed between prenatal exposure to antiemetics and the investigated childhood cancer sites, the analyses on the presence of hyperemesis gravidarum during pregnancy revealed an increased risk of ALL, neuroblastoma and non-Hodgkin lymphoma in the offspring. Hyperemesis gravidarum, defined as the most severe form of nausea and vomiting during pregnancy, is accompanied by weight loss, malnutrition, ketonuria and electrolyte imbalances [127, 128]. Ketonuria promotes oxidative stress, manifested by an increased presence of hydrogen peroxide products. Oxidative stress can lead to damage such as chromosomal aberrations to emerging cells, particularly during the early stages of embryogenesis [129, 130]. This might be an explanatory approach for an increased risk of childhood cancer. Further evidence is needed to determine whether the increased risk of childhood cancer is associated with the underlying disease, hyperemesis gravidarum, or the antiemetics itself.

### Hormones

The group of hormones included five differentiated analyses on AL, ALL and neuroblastoma. Three of these analyses included hormones of any type as exposure and two were restricted to the subtype of oral contraceptives. Meta-analyses demonstrate an increased risk of AL and neuroblastoma after prenatal exposure to hormones in general and for ALL in children prenatally exposed to oral contraceptives. The analysis on AL and hormones consists of several limitations, including moderate heterogeneity (I^2^ = 62.2%, *P =* 0.01) and low case numbers (range: 11–70) in all included studies. Results were mainly driven by the study from Pombo de Oliveira et al. [55], but the association still persists after excluding the study. This study with only 18 cases and four controls and therefore wide confidence intervals (OR = 8.76 (2.85; 26.93)) was conducted in a hospital-based case–control design. In contrast, all other included studies had a population-based case–control design.

One possible explanation for the identified risk increase could involve the mitogenic properties of steroid hormones. Mitogens like steroid hormones (e.g. estrogens) lead to increased cell division and proliferation, which is associated with an increased risk of breast and cervical cancer in adults [131–133]. Moreover, steroid hormones may induce epigenetic changes, thereby affecting the regulation of gene expression. A study published in 2022 [134] has investigated the role of prenatal estriol exposure in epigenetic reprogramming of the fetal mouse brain and reproductive tract. The study demonstrated that prenatal exposure to estriol induces alterations in uterine global gene expression profiles. At the adult stage, the in utero exposed mice showed a consistently altered expression profile including an enrichment of cancer-associated genes. In humans, exogenous hormone exposure during early pregnancy most commonly occurs in the context of assisted reproductive technologies. Although we did not specifically investigate hormone use in assisted reproduction, a recently published cohort study from France [135] reported an increased risk of leukemia in children born after fresh embryo transfer (HR 1.42, 95% CI 1.04–1.54) and an increased risk of acute lymphoblastic leukemia after frozen embryo transfer (HR 1.61, 95% CI 1.04–2.50), compared with naturally conceived children. However, as hormone use associated with assisted reproduction was not specifically examined in our study, no inferences regarding this exposure can be made. In addition, in our study, missing information regarding the intake period and duration of the hormones, the pooling of hormonally diverse compounds with varying biological functions, and an overall limited number of included cases, limit the interpretability of our findings.

### Antihypertensives

Maternal use of antihypertensives in pregnancy was found to be associated with an elevated risk of ALL and solid tumors in the offspring. Limitations of both analyses included low case and overall study numbers, suboptimal exposure assessment in the form of self-reports [39, 42, 51, 58] and limited consideration of potential confounders [58, 66]. The analysis on the heterogenous group of solid tumors comprises of five case–control studies with CNS tumors [66, 93], neuroblastoma [39, 58] and malignant germ cell tumors [42] as investigated childhood cancer sites. The recent high-quality study conducted by Askins et al. [93] used registry data to determine exposure, adjusted for several confounders including various diseases (e.g. hypertension and rheumatoid arthritis before index pregnancy) and found no association between maternal intake of antihypertensives in pregnancy and CNS tumors in children. However, the study revealed an increased risk of ALL for mothers who suffered from pre-eclampsia, particularly of the severe form [93]. Moreover, a population-based cohort study from 2024 [94] demonstrated an increased risk of ALL and additionally of non-Hodgkin lymphoma for children born to mothers with pre-eclampsia in pregnancy. Preeclampsia can impair fetal development through mechanisms such as restricted intrauterine growth, low birth weight, and preterm birth [136, 137]. These outcomes are often interrelated and partly result from induced early delivery, which is often indicated in cases of preeclampsia. Notably, these adverse perinatal outcomes have themselves been associated with an elevated risk of childhood cancer [138], suggesting that the observed link between preeclampsia and childhood cancer risk may be mediated, at least in part, by these intermediate factors rather than the medication exposure alone.

### Vitamin and mineral supplements

Prenatal exposure to vitamin and mineral supplements was observed to be associated with a reduced risk of CNS tumors, AL, ALL and solid tumors in the offspring. However, only a limited number of studies reported whether vitamin or mineral supplementation was prescribed due to an underlying deficiency. Most of the included studies used parental self-reports for exposure assessment, with the risk of recall bias and were low-quality studies. In addition, all results indicate slight to moderate heterogeneity between studies. The observed moderate heterogeneity in the analyses on prenatal exposure to vitamin and mineral supplements and the risk of ALL and CNS tumors may be attributable to differences in supplement composition, as studies evaluating maternal use of vitamins alone, in combination with folic acid, and/or iron were pooled together. Vitamins and minerals have an influence on various biochemical processes and physiological cell activities, in particular cell growth, DNA-synthesis, embryonic and fetal development, differentiation and apoptosis [139–142]. Especially Vitamin A and its active metabolite retinoic acid regulate genes for those cell functions and consequently can have a direct influence on the proliferation of cancer cells [140]. Furthermore, retinoic acid is considered to prevent cancer due to its capacity in influencing cellular growth [140, 141]. In contrast, high concentrations of vitamin A are assumed to be teratogenic [143]. Pregnancy is associated with an increased requirement for B vitamins, which function as coenzymes such as coenzyme A and as methyl group donors involved in DNA repair, purine and protein synthesis and DNA methylation [139, 144, 145]. In particular, vitamins like B_9_ and B_12_ protect the genome from chromosome and strand breaks, as their repair or incorrect repair may contribute to carcinogenesis [146, 147]. In addition to that, deficiency of folate can result in increased misincorporation of uracil into the DNA strand with the result of strand breaks. Similarly, deficiencies in vitamins B_2_, C and E, which have antioxidant properties, are associated with increased oxidative stress characterized by elevated levels of reactive oxygen and nitrogen species, and is known to be a carcinogenic [144, 148–150]. Iron deficiency is also associated with oxidative stress in fetal erythrocytes [143]. Collectively, vitamins in form of folate or vitamin deficiency anemia during pregnancy may contribute to an increased risk of childhood cancer, as reported in a recent study [91].

### Strengths and limitations

The present systematic review and meta-analysis comprises of specific strengths, including a large number of studies covering a wide publication period (1978–2025). A comprehensive search strategy, developed with the help of an experienced librarian, was employed to ensure that all relevant publications on this topic were identified, including extensive searches in two databases and screening of reference lists. In total, we were able to include 68 studies with more than 14 million study participants in our meta-analyses. The included studies often contained multiple separate analyses, such as subgroup analyses on histological and site-specific childhood cancers as well as on time of exposure. By extracting all of these estimates we were able to conduct precise analyses on different specific exposures and outcomes. In addition, we conducted comprehensive subgroup analyses on publication date, region, assessment of exposure and outcome, level of adjustment, and study quality to explore potential sources of heterogeneity. To determine the study quality, a self-developed quality assessment tool, which goes beyond existing tools by additionally considering important aspects such as latency periods, quality of statistical methods, training of interviewers, exposure assessment, and quality of control selection was used. Although analyses were frequently precluded by the limited number of available studies, we found no evidence of publication bias in the conducted analyses.

The overall limited number of studies represents the main limitation of this work, as fewer study participants were available for specific research questions. Moreover, the number of cases in general, but also of those with medication exposure, was frequently low, and some of the studies had methodological constraints, including less accurate methods of exposure assessment with potential risk of recall bias or limited/missing consideration of confounders. Furthermore, our study may be affected by survivor bias, as pregnancies ending in miscarriage or early fetal or neonatal death were not captured and therefore could not be included in the analyses. A further limitation is the variability in medication dosage during pregnancy. The estimates were combined irrespective of the amount and number of times the medicines were consumed. Due to a limited number of studies with investigation of same exposures and outcomes, for some groups of medication, analyses could only be conducted on the overall childhood cancer. This approach aggregated biologically distinct malignancies and therefore reduced etiological interpretability due to different causes of development and potentially different underlying mechanisms in the carcinogenesis of different cancers. Whenever possible, we performed analyses that were as specific as the data allowed. Further, the limited number of studies reporting exposure at specific time points prevented comprehensive trimester-level analyses for most medication types. This may weaken biological plausibility and could obscure potential time-dependent effects. However, the few analyses that could be performed stratified by trimester showed no evidence of such time-dependent effects.

## Conclusion

In conclusion, we observed multiple findings indicating increased childhood cancer risks following prenatal exposure to various types of medication, and reduced risks associated with prenatal exposure to vitamins and mineral supplements. Most relevant findings include the increased risk of ALL in children prenatally exposed to antibiotics of all kinds as well as the reduced risk of ALL and CNS tumors in children whose mothers had taken vitamin and mineral supplements during pregnancy. Despite vitamin and mineral supplements, the use of other types of medication during pregnancy is accompanied by greater uncertainty regarding their safety and administration should contain an explicit indication as well as a risk–benefit analysis. The observed protective effect of maternal supplementation of vitamin and mineral supplements on the risk of ALL and CNS tumors in children supports current clinical guidelines recommending vitamin supplementation, particularly folic acid, during pregnancy. No definitive clinical recommendation can be made regarding the use of antibiotics during pregnancy, as their administration is clearly indicated in the context of severe bacterial infections, where the expected benefits outweigh potential risks. Moreover, the extent to which the underlying infection itself contributes to the risk of childhood cancer needs to be considered in future research. This consideration extends to all medication groups assessed in this review: maternal illness, or the interplay between the illness and its treatment, may contribute to the observed associations, rather than the medication alone.

Given the heterogeneity of childhood cancers and the likelihood of multifactorial pathways, future research should aim to disentangle medication effects from those of underlying maternal health conditions, apply valid exposure assessment with careful attention to timing, and adequately control for relevant confounders, including co-morbidities and concurrent medications. Such studies are essential to more precisely determine whether, and under what circumstances, prenatal medication exposure contributes to childhood cancer risk.

## Supplementary Information

Supplementary Material 1: Supplementary Figure 1. Prenatal exposure to analgesics and the risk of childhood cancer. Abbreviations: ES, estimate; n.a., not available. Supplementary Figure 2. Prenatal exposure to antibiotics and the risk of childhood cancer. Abbreviations: ES, estimate; ^1^estimates were calculated with four-square table; ^*^ calculation of crude estimates. Supplementary Figure 3. Prenatal exposure to antiemetics and the risk of childhood cancer. Abbreviations: ES, estimate; n.a., not available; ^1^estimates were calculated with four-square table; ^*^ calculation of crude estimates. Supplementary Figure 4. Prenatal exposure to antihistamines and the risk of childhood cancer. Abbreviations: ES, estimate; n.a., not available; ^1^estimates were calculated with four-square table; ^*^ calculation of crude estimates. Supplementary Figure 5. Prenatal exposure to antihypertensives and the risk of childhood cancer. Abbreviations: ES, estimate; n.a., not available. Supplementary Figure 6. Prenatal exposure to antiretroviral HIV-drugs and the risk of childhood cancer. Abbreviations: ES, estimate; n.a., not available; HIV, human immunodeficiency virus; ^*^ calculation of crude estimates. Supplementary Figure 7. Prenatal exposure to cold or cough remedies and the risk of childhood cancer. Abbreviations: ES, estimate; n.a., not available; ^1^estimates were calculated with four-square table; ^*^ calculation of crude estimates. Supplementary Figure 8. Prenatal exposure to diuretics and the risk of childhood cancer. Abbreviations: ES, estimate; n.a., not available; ^1^estimates were calculated with four-square table; ^*^calculation of crude estimates. Supplementary Figure 9. Prenatal exposure to folic acid supplements and the risk of childhood cancer. Abbreviations: ES, estimate; n.a., not available. Supplementary Figure 10. Prenatal exposure to hormones and the risk of childhood cancer. Abbreviations: ES, estimate; n.a., not available; ^1^estimates were calculated with four-square table; ^*^calculation of crude estimates. Supplementary Figure 11. Prenatal exposure to iron supplements and the risk of childhood cancer. Abbreviations: ES, estimate; n.a., not available. Supplementary Figure 12. Prenatal exposure to nervous system medication and the risk of childhood cancer. Abbreviations: ES, estimate; n.a., not available; ^a^estimates were calculated with four-square table; ^*^calculation of crude estimates;^1^ nervous system medication, ^2^ sedatives/tranquilizer, ^3^ antidepressants/anxiety medication, ^4^ neurally active drugs, ^5^ barbiturates, ^6^ sleeping pills/tranquillizers, ^7^ hypnotics and anxiolytics, ^8^ antidepressants, ^9^ sedatives, ^10^ tranquilizer, ^11^ CNS depressants, ^12^ sedatives, tranquilizers, or sleeping pills. Supplementary Figure 13. Prenatal exposure to vitamin and mineral supplements and the risk of childhood cancer. Abbreviations: ES, estimate; n.a., not available; ^1^estimates were calculated with four-square table; ^*^ calculation of crude estimates. Supplementary Figure 14. Publication bias analysis on antibiotics and the risk of ALL. Abbreviations: Conf, confidence, eff, effect, Std. err., standard error, logor, log odds ratio, MSE, mean square error, P, P-value, se, standard error, SND, standard normal deviate. Supplementary Figure 15. Publication bias analysis on vitamin and mineral supplements and the risk of ALL. Abbreviations: Conf, confidence, eff, effect, Std. err., standard error, logor, log odds ratio, MSE, mean square error, P, P-value, se, standard error, SND, standard normal deviate. Supplementary Figure 16. Publication bias analysis on vitamin and mineral supplements and the risk of CNS tumors. Abbreviations: Conf, confidence, eff, effect, Std. err., standard error, logor, log odds ratio, MSE, mean square error, P, P-value, se, standard error, SND, standard normal deviate. Supplementary Figure 17. Prenatal exposure to antibiotics in different trimesters and the risk of ALL. Abbreviations: ES, estimate; ALL, acute lymphoblastic leukemia; n.a., not available. Supplementary Figure 18. Prenatal exposure to analgesics and the risk of different childhood cancer sites. Abbreviations: ES, estimate; ALL, acute lymphoblastic leukemia; AML, acute myeloid leukemia; CNS tumors, central nervous system tumors; n.a., not available; investigated childhood cancer site:^a^ neuroblastoma, ^b^ early age leukemia, ^c^ infant leukemia, ^d^ childhood brain tumors, ^e^ neuroblastoma. Supplementary Figure 19. Prenatal exposure to antiemetics and the risk of different childhood cancer sites. Abbreviations: ES, estimate; ALL, acute lymphoblastic leukemia; AML, acute myeloid leukemia; ANLL, acute non-lymphoblastic leukemia; CNS tumors, central nervous system tumors; n.a., not available; investigated childhood cancer site: ^a^ AML, ^b^ ANLL. Supplementary Figure 20. Prenatal exposure to antihypertensives and the risk of ALL and solid tumors in children. Abbreviations: ES, estimate; ALL, acute lymphoblastic leukemia; CNS tumors, central nervous system tumors; n.a., not available; investigated childhood cancer site: ^a^CNS tumors, ^b^ neuroblastoma, ^c^ malignant germ cell tumors. Supplementary Figure 21. Prenatal exposure to hormones and the risk of different childhood cancer sites. Abbreviations: ES, estimate; ALL, acute lymphoblastic leukemia; AML, acute myeloid leukemia; ANLL, acute non-lymphoblastic leukemia; n.a., not available; investigated childhood cancer site: ^a^ AML, ^b^ ANLL; ^1^ estimates were calculated with four-square table; ^*^ calculation of crude estimates. Supplementary Figure 22. Prenatal exposure to antihistamines and the risk of acute leukemia and CNS tumors in children. Abbreviations: ES, estimate; ALL, acute lymphoblastic leukemia; ANLL, acute non-lymphoblastic leukemia; CNS tumors, central nervous system tumors; n.a., not available; investigated childhood cancer site: ^a^ ANLL, ^b^ ALL. Supplementary Figure 23. Prenatal exposure to diuretics and the risk of CNS tumors in children. Abbreviations: ES, estimate; CNS tumors, central nervous system tumors; n.a., not available. Supplementary Figure 24. Prenatal exposure to folic acid supplements and the risk of different childhood cancer sites. Abbreviations: ES, estimate; ALL, acute lymphoblastic leukemia; AML, acute myeloid leukemia; ANLL, acute non-lymphoblastic leukemia; CNS tumors, central nervous system tumors; n.a., not available; investigated childhood cancer site:^a^ ALL, ^b^ ANLL. Supplementary Figure 25. Prenatal exposure to nervous system medication and the risk of different childhood cancer sites. Abbreviations: ES, estimate; ALL, acute lymphoblastic leukemia; AML, acute myeloid leukemia; ANLL, acute non-lymphoblastic leukemia; n.a., not available; ^*^ calculation of crude estimates; investigated childhood cancer site: ^a^ AML; ^b^ ANLL; investigated type of nervous system medication: ^1^nervous system medication, ^2^ sedatives, ^3^ hypnotics and anxiolytics, ^4^CNS depressants,^5^ sedatives, tranquilizers, or sleeping pills, ^6^ antidepressants/anxiety medication, ^7^neurally active drugs,^8^ barbiturates, ^9^ sleeping pills/tranquillizers. Supplementary Table 1. Search Strategy. Supplementary Table 2. Quality of included studies. Supplementary Table 3. Exclusion of single studies. Supplementary Table 4. Stratification by study period. Supplementary Table 5. Stratification by study region. Supplementary Table 6. Stratification by study design. Supplementary Table 7. Stratification by exposure method. Supplementary Table 8. Stratification by controlling for confounders. Supplementary Table 9. Stratification by study quality. Supplementary Table 10. Stratification by outcome assessment.

## Data Availability

The datasets used and/or analysed during the current study are available from the corresponding author on reasonable request.

## Abbreviations

AL: Acute leukemia
ALL: Acute lymphoblastic leukemia
CNS: Central nervous system
CI: Confidence interval
ES: Estimate
HR: Hazard ratio
IRR: Incidence rate ratios
NOS: Newcastle-Ottawa Scale
OR: Odds ratio
RR: Relative risk
ROBINS-E: Risk Of Bias in Non-randomized Studies Tools of Exposure
ROBINS-I: Risk Of Bias in Non-randomized Studies Tools of Intervention
SD: Standard deviation
SE: Standard error
SIR: Standardized incidence ratio
