# Supplementary material for: Prenatal exposure to medication and risk of childhood cancer – a systematic review and meta-analysis
Source: BMC Cancer. 2025 Nov 21;25:1841. doi: 10.1186/s12885-025-15316-0 (PMC12667062; doi:10.1186/s12885-025-15316-0)
Supplement: Supplementary file 1 — Supplementary Material 1: Supplementary Figure 1. Prenatal exposure to analgesics and the risk of childhood cancer. Abbreviations: ES, estimate; n.a., not available. Supplementary Figure 2. Prenatal exposure to antibiotics and the risk of childhood cancer. Abbreviations: ES, estimate; 1estimates were calculated with four-square table; * calculation of crude estimates. Supplementary Figure 3. Prenatal exposure to antiemetics and the risk of childhood cancer. Abbreviations: ES, estimate; n.a., not available; 1estimates were calculated with four-square table; * calculation of crude estimates. Supplementary Figure 4. Prenatal exposure to antihistamines and the risk of childhood cancer. Abbreviations: ES, estimate; n.a., not available; 1estimates were calculated with four-square table; * calculation of crude estimates. Supplementary Figure 5. Prenatal exposure to antihypertensives and the risk of childhood cancer. Abbreviations: ES, estimate; n.a., not available. Supplementary Figure 6. Prenatal exposure to antiretroviral HIV-drugs and the risk of childhood cancer. Abbreviations: ES, estimate; n.a., not available; HIV, human immunodeficiency virus; * calculation of crude estimates. Supplementary Figure 7. Prenatal exposure to cold or cough remedies and the risk of childhood cancer. Abbreviations: ES, estimate; n.a., not available; 1estimates were calculated with four-square table; * calculation of crude estimates. Supplementary Figure 8. Prenatal exposure to diuretics and the risk of childhood cancer. Abbreviations: ES, estimate; n.a., not available; 1estimates were calculated with four-square table; *calculation of crude estimates. Supplementary Figure 9. Prenatal exposure to folic acid supplements and the risk of childhood cancer. Abbreviations: ES, estimate; n.a., not available. Supplementary Figure 10. Prenatal exposure to hormones and the risk of childhood cancer. Abbreviations: ES, estimate; n.a., not available; 1estimates were calculated with four-square table; *c [file 12885_2025_15316_MOESM1_ESM.zip › Supplementary Table 1 Search Strategy_revised.docx]

| **PubMed** | **Web of Science** |
| --- | --- |
| “Neoplasms”[Mesh] AND (“Pregnancy”[Mesh] OR “Maternal Exposure”[Mesh] OR “Prenatal Exposure Delayed Effects”[Mesh]) AND (“Child”[Mesh] OR “Adolescent”[Mesh] OR “Infant”[Mesh]) AND ("Pharmaceutical preparations"[Mesh] OR "Cannabinoids"[Mesh] OR "Analgesics"[Mesh] OR "Antacids"[Mesh] OR "Anti-Anxiety agents"[Mesh] OR "Anti-Arrhythmia Agents "[Mesh] OR "Anti-Bacterial Agents"[Mesh] OR "Anticoagulants"[Mesh] OR "Fibrinolytic Agents"[Mesh] OR "Anticonvulsants"[Mesh] OR "Antidepressive Agents"[Mesh] OR "Antidiarrheals"[Mesh] OR "Antiemetics"[Mesh] OR "Antifungal Agents"[Mesh] OR "Histamine Antagonists"[Mesh] OR "Antihypertensive Agents"[Mesh] OR "Anti-Inflammatory Agents"[Mesh] OR "Antineoplastic Agents"[Mesh] OR "Antipsychotic Agents"[Mesh] OR "Antipyretics"[Mesh] OR "Antiviral Agents"[Mesh] OR "Anti-Retroviral Agents"[Mesh] OR "Barbiturates"[Mesh] OR "Adrenergic beta-Antagonists"[Mesh] OR "Bronchodilator Agents"[Mesh] OR "Adrenal Cortex Hormones"[Mesh] OR "Cytostatic Agents"[Mesh] OR "Nasal Decongestants"[Mesh] OR "Diuretics"[Mesh] OR "Expectorants"[Mesh] OR "Hormones"[Mesh] OR "Hypoglycemic Agents"[Mesh] OR "Immunosuppressive Agents"[Mesh] OR "Laxatives"[Mesh] OR "Muscle Relaxants, central"[Mesh] OR "Hypnotics and Sedatives"[Mesh] OR "Gonadal Steroid Hormones"[Mesh] OR "Sleep Aids, Pharmaceutical"[Mesh] OR "Reverse Transcriptase Inhibitors"[Mesh] OR "Tranquilizing Agents"[Mesh] OR "Folic Acid"[Mesh] OR "Vitamins"[Mesh] OR "Blood transfusion"[Mesh] OR “Platelet Transfusion”[Mesh] OR “Erythrocyte Transfusion”[Mesh] OR “Leukocyte Transfusion”[Mesh] OR ”Lymphocyte Transfusion”[Mesh] OR “Blood Component Transfusion”[Mesh] OR “Blood Transfusion, Intrauterine”[Mesh] OR “Exchange Transfusion, Whole Blood”[Mesh]) AND ("humans"[Mesh] AND English[lang]) NOT "case reports" [Publication Type] NOT "COVID-19" NOT "SARS-CoV-2" | (TS=(children) OR TS=(adolescent) OR TS=(infant)) AND (TS=(pregnancy) OR TS=(maternal exposure) OR TS=(Prenatal Exposure Delayed Effects) OR TS=(prenatal) AND TS=(exposure)) AND (TS=(neoplasms) OR TS=(cancer)) AND (TS=(Pharmaceutical preparations) OR TS=(Cannabinoids) OR TS=(Analgesics) OR TS=(Antacids) OR TS=(Anti-Anxiety agents) OR TS=(Anti-Arrhythmia Agents) OR TS=(Anti-Bacterial Agents) OR TS=(Anticoagulants) OR TS=(Fibrinolytic Agents) OR TS=(Anticonvulsants) OR TS=(Antidepressive Agents) OR TS=(Antidiarrheals) OR TS=(Antiemetics) OR TS=(Antifungal Agents) OR TS=(Histamine Antagonists) OR TS=(Antihypertensive Agents) OR TS=(Anti-Inflammatory Agents) OR TS=(Antineoplastic Agents) OR TS=(Antipsychotic Agents) OR TS=(Antipyretics) OR TS=(Antiviral Agents) OR TS=(Anti-Retroviral Agents) OR TS=(Barbiturates) OR TS=(Adrenergic beta-Antagonists) OR TS=(Bronchodilator Agents) OR TS=(Adrenal Cortex Hormones) OR TS=(Cytostatic Agents) OR TS=(Nasal Decongestants) OR TS=(Diuretics) OR TS=(Expectorants) OR TS=(Hormones) OR TS=(Hypoglycemic Agents) OR TS=(Immunosuppressive Agents) OR TS=(Laxatives) OR TS=(Muscle Relaxants, central) OR TS=(Hypnotics and Sedatives) OR TS=(Gonadal Steroid Hormones) OR TS=(Sleep Aids, Pharmaceutical) OR TS=(Reverse Transcriptase Inhibitors) OR TS=(Tranquilizing Agents) OR TS=(Folic Acid) OR TS=(Vitamins) OR TS=(Blood transfusion) OR TS=(Platelet Transfusion) OR TS=(Erythrocyte Transfusion) OR TS=(Leukocyte Transfusion) OR TS=(Lymphocyte Transfusion) OR TS=(Blood Component Transfusion) OR TS=(Blood Transfusion, Intrauterine) OR TS=(Exchange Transfusion, Whole Blood)) |

**Supplementary Table 1 Search Strategy**

Date of search: 15.07.2025
