# Supplementary material for: Prenatal exposure to medication and risk of childhood cancer – a systematic review and meta-analysis
Source: BMC Cancer. 2025 Nov 21;25:1841. doi: 10.1186/s12885-025-15316-0 (PMC12667062; doi:10.1186/s12885-025-15316-0)
Supplement: Supplementary file 1 — Supplementary Material 1: Supplementary Figure 1. Prenatal exposure to analgesics and the risk of childhood cancer. Abbreviations: ES, estimate; n.a., not available. Supplementary Figure 2. Prenatal exposure to antibiotics and the risk of childhood cancer. Abbreviations: ES, estimate; 1estimates were calculated with four-square table; * calculation of crude estimates. Supplementary Figure 3. Prenatal exposure to antiemetics and the risk of childhood cancer. Abbreviations: ES, estimate; n.a., not available; 1estimates were calculated with four-square table; * calculation of crude estimates. Supplementary Figure 4. Prenatal exposure to antihistamines and the risk of childhood cancer. Abbreviations: ES, estimate; n.a., not available; 1estimates were calculated with four-square table; * calculation of crude estimates. Supplementary Figure 5. Prenatal exposure to antihypertensives and the risk of childhood cancer. Abbreviations: ES, estimate; n.a., not available. Supplementary Figure 6. Prenatal exposure to antiretroviral HIV-drugs and the risk of childhood cancer. Abbreviations: ES, estimate; n.a., not available; HIV, human immunodeficiency virus; * calculation of crude estimates. Supplementary Figure 7. Prenatal exposure to cold or cough remedies and the risk of childhood cancer. Abbreviations: ES, estimate; n.a., not available; 1estimates were calculated with four-square table; * calculation of crude estimates. Supplementary Figure 8. Prenatal exposure to diuretics and the risk of childhood cancer. Abbreviations: ES, estimate; n.a., not available; 1estimates were calculated with four-square table; *calculation of crude estimates. Supplementary Figure 9. Prenatal exposure to folic acid supplements and the risk of childhood cancer. Abbreviations: ES, estimate; n.a., not available. Supplementary Figure 10. Prenatal exposure to hormones and the risk of childhood cancer. Abbreviations: ES, estimate; n.a., not available; 1estimates were calculated with four-square table; *c [file 12885_2025_15316_MOESM1_ESM.zip › Supplementary Table 10 Stratification by outcome assessment.docx]

**Supplementary Table 10 Stratification by outcome assessment**

| **Model** | **Yes (ES (95%CI))** | **n** | **I^2^** | **P value** | **No (ES (95%CI))** | **n** | **I^2^** | **P value** |
| --- | --- | --- | --- | --- | --- | --- | --- | --- |
| Analgesics and risk of ALL | 1.09 (0.88, 1.36) | 5 | 0.0 % | 0.595 | 1.32 (1.01, 1.72) | 2 | 0.0 % | 0.526 |
| Analgesics and risk of AML | 0.90 (0.63, 1.28) | 3 | 0.0 % | 0.672 | 0.75 (0.36, 1.56) | 1 |  |  |
| Analgesics and risk of CNS tumors | 0.98 (0.73, 1.31) | 5 | 0.0 % | 0.667 | 1.54 (0.81, 2.94) | 1 |  |  |
| Analgesics and risk of neuroblastoma | 1.28 (0.90, 1.82) | 3 | 41.1 % | 0.183 | 1.99 (1.07, 3.70) | 1 |  |  |
| Analgesics and risk of lymphoma | 0.92 (0.47, 1.81) | 2 | 0.0 % | 0.462 | 5.05 (2.16, 11.81) | 1 |  |  |
| Antibiotics and risk of ALL | 1.14 (1.05, 1.25) | 10 | 11.0 % | 0.326 | 1.01 (0.70, 1.46) | 2 | 57.4 % | 0.125 |
| Antibiotics and risk of AML | 1.29 (0.69, 2.44) | 4 | 82.9 % | 0.001 | 1.21 (0.81, 1.80) | 1 |  |  |
| Antibiotics and risk of CNS tumors | 1.10 (0.92, 1.33) | 7 | 44.7 % | 0.093 | 0.93 (0.57, 1.51) | 1 |  |  |
| Antibiotics and risk of medulloblastoma | 1.63 (1.08, 2.45) | 3 | 0.0 % | 0.465 | 1.47 (0.79, 2.74) | 1 |  |  |
| Antibiotics and risk of lymphoma | 1.29 (0.91, 1.83) | 4 | 0.0 % | 0.471 | 0.92 (0.54, 1.56) | 1 |  |  |
| Antibiotics and risk of neuroblastoma | 1.45 (0.90, 2.34) | 4 | 65.9 % | 0.032 | 1.15 (0.70, 1.89) | 1 |  |  |
| Antibiotics and risk of renal tumors | 0.93 (0.69, 1.25) | 3 | 0.0 % | 0.852 | 0.95 (0.52, 1.72) | 1 |  |  |
| Penicillin and risk of leukemia | 1.03 (0.80, 1.34) | 2 | 35.5 % | 0.213 | 0.98 (0.79, 1.21) | 1 |  |  |
| Penicillin and risk of solid tumors | 1.09 (0.77, 1.55) | 2 | 47.4 % | 0.168 | 1.73 (0.95, 3.16) | 1 |  |  |
| Antiemetics and risk of ALL | 1.19 (0.94, 1.51) | 4 | 0.0 % | 0.582 | 1.20 (0.80, 1.80) | 1 |  |  |
| Antihistamines and risk of CNS tumors | 0.93 (0.68, 1.27) | 4 | 0.0 % | 0.962 | 3.90 (0.62, 24.46) | 1 |  |  |
| Antihistamines and risk of leukemia | 1.46 (0.96, 2.23) | 2 | 0.0 % | 0.754 | 1.70 (0.49, 5.84) | 1 |  |  |
| Folic acid supplements and risk of ALL | 0.93 (0.60, 1.45) | 5 | 86.8 % | 0.000 | 0.40 (0.21, 0.75) | 1 |  |  |
| Folic acid supplements and risk of CNS tumors | 0.83 (0.61, 1.13) | 5 | 55.6 % | 0.061 | 0.83 (0.60, 1.14) | 1 |  |  |
| Hormones and risk of leukemia | 1.56 (1.05, 2.32) | 6 | 68.5 % | 0.007 | 1.34 (0.81, 2.21) | 1 |  |  |
| Hormones and risk of ALL | 1.41 (1.05, 1.90) | 2 | 0.0 % | 0.643 | 0.80 (0.62, 1.03) | 2 | 0.0 % | 0.492 |
| Oral contraceptives and risk of ALL | 1.26 (1.00, 1.59) | 4 | 0.0 % | 0.610 | 1.70 (0.68, 4.26) | 1 |  |  |
| Nervous system medication and risk of leukemia | 1.16 (0.17, 7.66) | 2 | 84.0 % | 0.012 | 0.99 (0.56, 1.75) | 1 |  |  |
| Nervous system medication and risk of ALL | 1.41 (0.81, 2.44) | 3 | 0.0 % | 0.512 | 2.18 (0.53, 8.93) | 2 | 72.2 % | 0.058 |
| Vitamin and mineral supplements and risk of ALL | 0.84 (0.69, 1.03) | 8 | 56.8 % | 0.023 | 0.67 (0.28, 1.60) | 2 | 86.3 % | 0.007 |
| Vitamin and mineral supplements and risk of CNS tumors | 0.83 (0.67, 1.03) | 7 | 61.7 % | 0.016 | 0.43 (0.11, 1.67) | 2 | 85.6 % | 0.008 |
| Vitamin C supplements and risk of CNS tumors | 0.82 (0.30, 2.26) | 2 | 82.0 % | 0.018 | 0.83 (0.59, 1.17) | 1 |  |  |
| Vitamin A supplements and risk of solid tumors | 0.56 (0.37, 0.85) | 2 | 30.1 % | 0.232 | 0.88 (0.62, 1.25) | 1 |  |  |
| Vitamin C supplements in trimester 1 and risk of CNS tumors | 0.81 (0.49, 1.33) | 3 | 50.0 % | 0.135 | 0.80 (0.57, 1.12) | 1 |  |  |
| Vitamin C supplements in trimester 2/3 and risk of CNS tumors | 0.85 (0.49, 1.49) | 3 | 37.7 % | 0.201 | 0.79 (0.56, 1.12) | 1 |  |  |

Yes: outcome assessment based on registry data or pathological confirmation, no: outcome assessment based on other sources

Abbreviations: CI, confidence interval; OR, odds ratio; ALL, acute lymphocytic leukemia; AML, acute myeloid leukemia; CNS, central nervous system
