# Supplementary material for: Prenatal exposure to medication and risk of childhood cancer – a systematic review and meta-analysis
Source: BMC Cancer. 2025 Nov 21;25:1841. doi: 10.1186/s12885-025-15316-0 (PMC12667062; doi:10.1186/s12885-025-15316-0)
Supplement: Supplementary file 1 — Supplementary Material 1: Supplementary Figure 1. Prenatal exposure to analgesics and the risk of childhood cancer. Abbreviations: ES, estimate; n.a., not available. Supplementary Figure 2. Prenatal exposure to antibiotics and the risk of childhood cancer. Abbreviations: ES, estimate; 1estimates were calculated with four-square table; * calculation of crude estimates. Supplementary Figure 3. Prenatal exposure to antiemetics and the risk of childhood cancer. Abbreviations: ES, estimate; n.a., not available; 1estimates were calculated with four-square table; * calculation of crude estimates. Supplementary Figure 4. Prenatal exposure to antihistamines and the risk of childhood cancer. Abbreviations: ES, estimate; n.a., not available; 1estimates were calculated with four-square table; * calculation of crude estimates. Supplementary Figure 5. Prenatal exposure to antihypertensives and the risk of childhood cancer. Abbreviations: ES, estimate; n.a., not available. Supplementary Figure 6. Prenatal exposure to antiretroviral HIV-drugs and the risk of childhood cancer. Abbreviations: ES, estimate; n.a., not available; HIV, human immunodeficiency virus; * calculation of crude estimates. Supplementary Figure 7. Prenatal exposure to cold or cough remedies and the risk of childhood cancer. Abbreviations: ES, estimate; n.a., not available; 1estimates were calculated with four-square table; * calculation of crude estimates. Supplementary Figure 8. Prenatal exposure to diuretics and the risk of childhood cancer. Abbreviations: ES, estimate; n.a., not available; 1estimates were calculated with four-square table; *calculation of crude estimates. Supplementary Figure 9. Prenatal exposure to folic acid supplements and the risk of childhood cancer. Abbreviations: ES, estimate; n.a., not available. Supplementary Figure 10. Prenatal exposure to hormones and the risk of childhood cancer. Abbreviations: ES, estimate; n.a., not available; 1estimates were calculated with four-square table; *c [file 12885_2025_15316_MOESM1_ESM.zip › Supplementary Table 2 Quality of included studies_revised.pdf]

Supplementary Table 2 Quality of included studies

			Study Design 0-6 P														
			Intervention Studies					Observational Studies									
Reference	Included in meta-analysis	Sum of Quality Score Maximal 45 Points (P)	Randomized, Double-Blinded 6 P	Randomized, Single-Blinded 5 P	Randomized, Unblinded 4 P	Not Randomized 2 P	Uncontrolled Experiment 1 P	Prospective Cohort 4 P	Retrospective Cohort 4 P	Case-Cohort 4 P	Register Study 4 P	Nested Case-Control 4 P	Population-Based Case-Control 3 P	Case-Control 2 P	Hospital-Based Case-Control 2 P	Cross-Sectional Study 1 P	Ecological Study 0 P
**Number of Studies (N=80)**																	
Ajrouche, 2014	1	**26.6**											3				
Amigou, 2012	1	**26.6**											3				
Askins, 2024	1	**28.0**											3				
Avagyan, 2023	1	**18.2**													2		
Bailey, 2017	0	**25.2**											3				
Bauer, 2020	1	**24.4**											3				
Benhammou, 2008	1	**26.8**						4									
Bonaventure, 2015	1	**22.6**											3				
Buckley, 1989	1	**19.2**											3				
Bunin, 2006	1	**26.4**											3				
Cheng, 2023	1	**27.4**											3				
Cocco, 1996	0	**21.8**											3				
Cook, 2004	1	**25.6**											3				
Cordier, 1994	1	**19.6**											3				
Couto, 2015	1	**24.4**													2		
Dockerty, 2007	1	**19.8**											3				
Giuffrè, 1990	0	**11.0**													2		
Gold, 1978	1	**18.8**													2		
Goldhaber, 1990	1	**23.6**													2		
Gudnadottir, 2025	1	**28.4**							4								
Gradel, 2015	1	**25.4**										4					
Greenop, 2014	0	**24.4**											3				
Hargreave, 2018	1	**31.4**							4								
Hargreave, 2019	0	**30.2**							4								
Hargreave, 2022	0	**30.2**							4								
Hjorth, 2022	1	**34.6**							4								
Hleyhel, 2016	1	**29.8**						4									
Ivy, 2015	1	**23.4**							4								
Johnson, 2009	1	**23.8**											3				
Jung, 2023	1	**26.4**											3				
Kaatsch, 2010	1	**22.2**											3				
Kramer, 1987	1	**22.4**											3				
Kuijten, 1990	1	**20.0**											3				
Kwan, 2007	1	**21.6**											3				
Linabery, 2010	1	**23.4**											3				
McCredie, 1994	1	**23.8**											3				
McKinney, 1985	1	**20.8**											3				
McKinney, 1999	1	**26.2**											3				
Metayer, 2014	0	**25.6**											3				
Michalek, 1996	1	**23.4**											3				
Milne, 2012	1	**24.8**											3				
Milne, 2010	1	**22.6**											3				
Momen, 2018	1	**34.2**							4								
Momen, 2015	1	**30.6**							4								
Mortensen, 2016	1	**29.4**							4								
Msallem, 2025	0	**25.4**											3				
Ognjanovic, 2010	1	**23.4**											3				
Olshan, 1999	0	**24.6**											3				
Olshan, 2002	1	**24.4**											3				
Orimoloye, 2023	1	**29.0**											3				
Orimoloye, 2024	1	**34.0**							4								
Ortega-García, 2010	1	**19.8**													2		
Platamone, 2024	1	**29.0**											3				
Pombo-de-Oliveira, 2006	1	**22.8**													2		
Preston-Martin, 1996	0	**25.4**											3				
Preston-Martin, 1998	1	**24.8**											3				
Qureshi, 2023	1	**29.0**											3				
Rios, 2016	0	**23.6**											3				
Robison, 1989	1	**22.6**											3				
Ross, 1996	0	**23.4**											3				
Schüz, 2001	1	**23.2**											3				
Schüz, 2007	1	**22.2**											3				
Schwartzbaum, 1992	1	**22.4**													2		
Seppälä, 2020	1	**26.2**											3				
Shankar, 2006	1	**26.6**											3				
Shaw, 2004	1	**22.6**											3				
Shu, 1995	1	**23.4**											3				
Shu, 2002	1	**25.6**											3				
Singer, 2016	1	**24.6**											3				
Sirirungreung, 2023	1	**26.4**											3				
Sirirungreung, 2024	1	**31.4**							4								
Stålberg, 2010	1	**28.0**											3				
Thapa, 1998	1	**29.8**							4								
Thompson, 2001	1	**19.2**											3				
van Steensel-Moll, 1985	1	**24.0**											3				
Vegrim, 2022	1	**36.4**							4								
Vienneau, 2016	1	**26.4**											3				
Wen, 2002	1	**27.4**											3				
Yan, 2022	1	**24.4**													2		
Ye, 2019	1	**31.4**							4								

	Sample Size 0-6 P							Outcome 0-6 P							Exposure 0-6 P							Control 0-6 P			
Reference	>1,000,000 6 P	>100,000 - ≤ 1,000,000 5 P	>10,000 - ≤ 100,000 4 P	>5,000 - ≤ 10,000 3 P	>1,000 - ≤ 5,000 2 P	>500 - ≤ 1,000 1 P	1 - 500 0 P / no Information	Trial Data / Pathologically confirmed 6 P	Accounting Data (e.g. Insurance) 5 P	Registry Data 4 P	Medical Documentation 3 P	Record (Death Certificate) 2 P	Self-Report 1 P	Aggregated Data / No Information 0 P	Laboratory test 6 P	Trial Data 5 P	Accounting Data 4 P	Registry Data 3 P	Medical Documentation 2 P	Self-Report 1 P	Aggregated Data / No Information 0 P	Controling for Similar Exposure 3 P	Control for Other Diseases in the Pathway 2 P	Control for other factors 1 P	No Controling 0 P
Ajrouche, 2014					2					4										1		3		1	
Amigou, 2012					2					4										1		3		1	
Askins, 2024			4							4								3					2	1	
Avagyan, 2023							0			4										1				1	
Bailey, 2017					2			6												1				1	
Bauer, 2020					2					4										1				1	
Benhammou, 2008			4							4									2					1	
Bonaventure, 2015					2						3								2					1	
Buckley, 1989							0	6												1					0
Bunin, 2006						1		6												1			2	1	
Cheng, 2023					2			6												1			2	1	
Cocco, 1996							0	6												1			2	1	
Cook, 2004					2			6												1				1	
Cordier, 1994							0				3									1				1	
Couto, 2015						1		6												1		3		1	
Dockerty, 2007							0			4										1				1	
Giuffrè, 1990							0	6											2						0
Gold, 1978							0	6												1					0
Goldhaber, 1990							0	6											2				2		
Gudnadottir, 2025		5								4								3						1	
Gradel, 2015					2					4								3						1	
Greenop, 2014					2						3									1		3		1	
Hargreave, 2018	6									4								3					2	1	
Hargreave, 2019	6									4								3						1	
Hargreave, 2022	6									4								3						1	
Hjorth, 2022		5								4								3				3	2	1	
Hleyhel, 2016			4							4									2			3		1	
Ivy, 2015					2					4								3							0
Johnson, 2009						1		6												1				1	
Jung, 2023							0	6												1		3		1	
Kaatsch, 2010					2					4										1				1	
Kramer, 1987							0	6												1					0
Kuijten, 1990							0			4										1				1	
Kwan, 2007						1				4										1				1	
Linabery, 2010						1		6												1				1	
McCredie, 1994							0	6												1				1	
McKinney, 1985					2					4									2						0
McKinney, 1999					2			6											2					1	
Metayer, 2014			4					6												1				1	
Michalek, 1996						1		6												1					0
Milne, 2012					2						3									1		3		1	
Milne, 2010					2					4										1				1	
Momen, 2018		5								4								3				3	2	1	
Momen, 2015	6									4								3						1	
Mortensen, 2016		5								4								3						1	
Msallem, 2025					2			6												1				1	
Ognjanovic, 2010						1		6												1				1	
Olshan, 1999					2			6												1				1	
Olshan, 2002					2			6												1				1	
Orimoloye, 2023		5								4								3					2	1	
Orimoloye, 2024	6								5								4						2	1	
Ortega-García, 2010							0			4										1				1	
Platamone, 2024			4					6										3					2	1	
Pombo-de-Oliveira, 2006						1		6												1				1	
Preston-Martin, 1996					2					4										1		3		1	
Preston-Martin, 1998					2			6												1				1	
Qureshi, 2023		5								4								3					2	1	
Rios, 2016					2					4										1				1	
Robison, 1989							0	6												1				1	
Ross, 1996						1		6												1				1	
Schüz, 2001					2					4										1				1	
Schüz, 2007					2					4										1				1	
Schwartzbaum, 1992						1		6												1				1	
Seppälä, 2020			4							4								3						1	
Shankar, 2006						1		6											2					1	
Shaw, 2004					2						3									1				1	
Shu, 1995						1		6												1				1	
Shu, 2002					2			6												1				1	
Singer, 2016					2			6												1				1	
Sirirungreung, 2023			4							4								3						1	
Sirirungreung, 2024	6								5								4							1	
Stålberg, 2010					2			6										3						1	
Thapa, 1998		5						6											2					1	
Thompson, 2001							0				3									1				1	
van Steensel-Moll, 1985					2			6												1				1	
Vegrim, 2022	6							6										3				3	2	1	
Vienneau, 2016						1		6										3						1	
Wen, 2002					2			6												1		3		1	
Yan, 2022							0	6												1		3		1	
Ye, 2019		5						6									4							1	

Maximal score of 45 points, quality score above the 4th quintile (≥28.5 = high quality) and below (<28.5 = low quality); mean value = 25.2; median = 24.7

Supplementary Table 2 Continued

Statistics 0-6 P					Methods 0-6 P						Reported Basic Characteristics 0-3 P																
Statistic appropriate (CI and/or P-value) 2 P	Statistical Power Indicated 1 P	Statistical Methods Applied 1 P	Statistical Methods Indicated 1 P	Primary Objective Defined 1 P	Good Selection (e.g. no Cancer) 1 P	Generalization of Results (Extern Validity) 1 P	Response 50+% 1 P	Follow-up of 5+ years and/or loss to follow up <	Blinded Evaluator / Trained Interviewer 1 P	Reference Group Randomized 1 P	Information on Study Population 0.2 P	Sex 0.2 P	Age 0.2 P	Age at Exposure (Medication) 0.2 P	Age at Diagnosis (Cancer) 0.2 P	Exposure Description (Medication) 0.2 P	Outcome Description (Cancer Site) 0.2 P	In- and Exclusion Criteria Indicated 0.2 P	Recruitment Described 0.2 P	Numbers Described 0.2 P	Reference Group Available 0.2 P	Information on Study Center 0.2 P	Multi-Center Study 0.2 P	Several Times of Investigation 0.2 P	Discussion of Bias and Limitations 0.2 P	Limitation, Confounder, Bias not Noted up to -6 P	Comments / Incidence or Prevalence
2		1	1	1	1	1	1		1	1	0.2	0.2	0.2	0.2		0.2	0.2	0.2	0.2	0.2	0.2	0.2	0.2		0.2		National Prescription Register Sample: 63025 controls, overall: 160484
2		1	1	1	1	1	1		1	1	0.2	0.2	0.2	0.2		0.2	0.2	0.2	0.2	0.2	0.2	0.2	0.2		0.2		
2		1	1	1	1	1	1			1	0.2		0.2			0.2	0.2	0.2	0.2	0.2	0.2	0.2			0.2		
2		1	1	1	1		1			1	0.2	0.2	0.2			0.2	0.2	0.2	0.2	0.2	0.2	0.2			0.2		
2		1	1	1	1	1	1		1	1	0.2	0.2	0.2			0.2	0.2	0.2	0.2	0.2	0.2	0.2			0.2		
2	1	1	1	1	1	1	1		1	1	0.2	0.2	0.2			0.2	0.2	0.2	0.2	0.2	0.2	0.2	0.2		0.2		
2	1	1	1	1	1	1	1				0.2	0.2	0.2	0.2	0.2	0.2	0.2	0.2	0.2	0.2	0.2	0.2	0.2		0.2		
2		1	1	1	1	1	1			1	0.2	0.2	0.2		0.2	0.2	0.2	0.2	0.2	0.2	0.2	0.2	0.2		0.2		
		1	1	1	1	1	1			1	0.2		0.2			0.2	0.2	0.2	0.2	0.2	0.2	0.2	0.2		0.2		
2		1	1	1	1	1	1		1	1	0.2		0.2	0.2		0.2	0.2	0.2	0.2	0.2	0.2	0.2	0.2		0.2		
2		1	1	1	1	1	1		1	1	0.2	0.2	0.2			0.2	0.2	0.2	0.2	0.2	0.2	0.2	0.2		0.2		
2		1	1	1	1					1	0.2						0.2	0.2	0.2	0.2	0.2	0.2	0.2		0.2		
2		1	1	1	1	1	1		1	1	0.2	0.2	0.2	0.2		0.2	0.2	0.2	0.2	0.2	0.2	0.2	0.2		0.2		
2		1	1	1	1	1	1			1	0.2	0.2	0.2		0.2	0.2	0.2	0.2	0.2	0.2	0.2	0.2	0.2		0.2		
2		1	1	1	1	1				1	0.2	0.2	0.2			0.2	0.2	0.2	0.2	0.2	0.2	0.2	0.2		0.2		
2		1	1	1	1	1	1			1	0.2					0.2	0.2	0.2	0.2	0.2	0.2	0.2			0.2		
				1						1	0.2					0.2	0.2			0.2	0.2					-2	No discussion of bias/limitations, methods not clearly described
2		1	1	1		1	1			1	0.2					0.2	0.2	0.2	0.2	0.2	0.2	0.2			0.2		
2		1	1	1	1	1	1			1	0.2	0.2	0.2		0.2	0.2	0.2	0.2	0.2	0.2	0.2	0.2	0.2		0.2		
2		1	1	1	1	1		1		1	0.2	0.2	0.2		0.2	0.2	0.2	0.2	0.2	0.2	0.2	0.2			0.2		
2		1	1	1	1	1	1			1	0.2	0.2	0.2	0.2		0.2	0.2	0.2	0.2	0.2	0.2	0.2			0.2		
2		1	1	1	1	1	1			1	0.2	0.2	0.2			0.2	0.2	0.2	0.2	0.2	0.2	0.2	0.2		0.2		
2		1	1	1	1	1		1		1	0.2	0.2	0.2	0.2		0.2	0.2	0.2	0.2	0.2	0.2	0.2			0.2		
2		1	1	1	1	1	1	1		1	0.2	0.2	0.2			0.2	0.2	0.2	0.2	0.2	0.2	0.2			0.2		
2		1	1	1	1	1	1	1		1	0.2	0.2	0.2			0.2	0.2	0.2	0.2	0.2	0.2	0.2			0.2		
2		1	1	1	1	1	1	1		1	0.2	0.2	0.2	0.2		0.2	0.2	0.2	0.2	0.2	0.2	0.2	0.2		0.2		
2		1	1	1	1	1	1			1	0.2	0.2	0.2	0.2	0.2	0.2	0.2	0.2	0.2	0.2	0.2	0.2	0.2		0.2		
2		1	1	1	1		1			1	0.2	0.2	0.2		0.2	0.2	0.2	0.2	0.2	0.2	0.2	0.2			0.2		4 cases of cancer
2		1	1	1	1	1	1			1	0.2	0.2	0.2	0.2	0.2	0.2	0.2	0.2	0.2	0.2	0.2	0.2	0.2		0.2		
2		1	1	1	1	1	1		1	1	0.2	0.2	0.2			0.2	0.2	0.2	0.2	0.2	0.2	0.2	0.2		0.2		
2		1	1	1	1	1	1			1	0.2	0.2	0.2			0.2	0.2	0.2	0.2	0.2	0.2	0.2			0.2		
2		1	1	1	1	1	1		1	1	0.2	0.2	0.2		0.2	0.2	0.2	0.2	0.2	0.2	0.2	0.2			0.2		
2		1	1	1	1	1	1			1	0.2					0.2	0.2	0.2	0.2	0.2	0.2	0.2	0.2		0.2		
2		1	1	1	1	1	1			1	0.2	0.2	0.2		0.2	0.2	0.2	0.2	0.2	0.2	0.2	0.2	0.2		0.2		
2		1	1	1	1	1	1			1	0.2	0.2	0.2			0.2	0.2	0.2	0.2	0.2	0.2	0.2	0.2		0.2		
2	1	1	1	1	1	1	1		1	1	0.2					0.2	0.2	0.2	0.2	0.2	0.2	0.2			0.2		
2		1	1	1	1	[1]	1			1	0.2					0.2	0.2	0.2	0.2	0.2	0.2	0.2	0.2				
2		1	1	1	1	1	1		1	1	0.2		0.2			0.2	0.2	0.2	0.2	0.2	0.2	0.2	0.2		0.2		
2		1	1	1	1	1				1	0.2	0.2	0.2	0.2		0.2	0.2	0.2	0.2	0.2	0.2	0.2	0.2		0.2		
2		1	1	1	1	1	1		1	1	0.2	0.2	0.2		0.2	0.2	0.2	0.2	0.2	0.2	0.2	0.2			0.2		
2		1	1	1	1	1	1			1	0.2	0.2	0.2	0.2	0.2	0.2	0.2	0.2	0.2	0.2	0.2	0.2	0.2		0.2		
2		1	1	1	1	1	1			1	0.2	0.2	0.2	0.2		0.2	0.2	0.2	0.2	0.2	0.2	0.2	0.2		0.2		
2		1	1	1	1	1	1	1		1	0.2	0.2	0.2			0.2	0.2	0.2	0.2	0.2	0.2	0.2			0.2		
2		1	1	1	1	1	1	1		1	0.2	0.2	0.2	0.2		0.2	0.2	0.2	0.2	0.2	0.2	0.2	0.2		0.2		
2		1	1	1	1	1	1	1		1	0.2	0.2	0.2		0.2	0.2	0.2	0.2	0.2	0.2	0.2	0.2			0.2		
2		1	1	1	1	1	1		1	1	0.2	0.2	0.2			0.2	0.2	0.2	0.2	0.2	0.2	0.2	0.2		0.2		
2		1	1	1	1	1	1			1	0.2	0.2	0.2			0.2	0.2	0.2	0.2	0.2	0.2	0.2	0.2		0.2		
2		1	1	1	1	1	1			1	0.2	0.2	0.2	0.2		0.2	0.2	0.2	0.2	0.2	0.2	0.2	0.2		0.2		
2		1	1	1	1	1	1			1	0.2		0.2	0.2		0.2	0.2	0.2	0.2	0.2	0.2	0.2	0.2		0.2		
2		1	1	1	1	1	1			1	0.2		0.2			0.2	0.2	0.2	0.2	0.2	0.2	0.2			0.2		Pharmaceutical registry sample for birth year (n=63025), overall number: 160500
2		1	1	1	1	1	1	1		1	0.2		0.2			0.2	0.2	0.2	0.2	0.2	0.2	0.2			0.2		
2		1	1	1		1	1		1	1	0.2	0.2	0.2	0.2	0.2	0.2	0.2	0.2	0.2	0.2	0.2	0.2	0.2		0.2		
2		1	1	1	1	1				1	0.2		0.2			0.2	0.2	0.2	0.2	0.2	0.2	0.2			0.2		
2		1	1	1	1	1	1			1	0.2	0.2	0.2	0.2	0.2	0.2	0.2	0.2	0.2	0.2	0.2	0.2	0.2		0.2		
2		1	1	1	1	1	1			1	0.2	0.2	0.2			0.2	0.2	0.2	0.2	0.2	0.2	0.2	0.2		0.2		
2		1	1	1	1	1	1			1	0.2	0.2	0.2	0.2	0.2	0.2	0.2	0.2	0.2	0.2	0.2	0.2	0.2		0.2		
2		1	1	1	1	1	1			1	0.2		0.2			0.2	0.2	0.2	0.2	0.2	0.2	0.2			0.2		
2		1	1	1	1	1	1		1	1	0.2	0.2	0.2	0.2		0.2	0.2	0.2	0.2	0.2	0.2	0.2	0.2		0.2		
2		1	1	1	1	1	1			1	0.2	0.2	0.2		0.2	0.2	0.2	0.2	0.2	0.2	0.2	0.2	0.2		0.2		
2		1	1	1	1	1	1			1	0.2		0.2		0.2	0.2	0.2	0.2	0.2	0.2	0.2	0.2	0.2		0.2		
2		1	1	1	1	1	1		1	1	0.2	0.2	0.2			0.2	0.2	0.2	0.2	0.2	0.2	0.2			0.2		
2		1	1	1	1	1	1			1	0.2	0.2	0.2			0.2	0.2	0.2	0.2	0.2	0.2	0.2			0.2		
2	1	1	1	1			1		1	1	0.2	0.2	0.2		0.2	0.2	0.2	0.2	0.2	0.2	0.2	0.2			0.2		
2		1	1	1	1	1	1			1	0.2	0.2	0.2			0.2	0.2	0.2	0.2	0.2	0.2	0.2			0.2		
2	1	1	1	1	1	1	1		1	1	0.2	0.2	0.2		0.2	0.2	0.2	0.2	0.2	0.2	0.2	0.2	0.2		0.2		
2		1	1	1	1	1	1		1	1	0.2	0.2	0.2		0.2	0.2	0.2	0.2	0.2	0.2	0.2	0.2	0.2		0.2		
2		1	1	1	1	1	1			1	0.2	0.2	0.2			0.2	0.2	0.2	0.2	0.2	0.2	0.2	0.2		0.2		
2		1	1	1	1	1	1		1	1	0.2	0.2	0.2		0.2	0.2	0.2	0.2	0.2	0.2	0.2	0.2	0.2		0.2		
2		1	1	1	1	1	1			1	0.2	0.2	0.2		0.2	0.2	0.2	0.2	0.2	0.2	0.2	0.2	0.2		0.2		
2		1	1	1	1	1	1			1	0.2	0.2	0.2		0.2	0.2	0.2	0.2	0.2	0.2	0.2	0.2			0.2		
2		1	1	1	1	1		1		1	0.2	0.2	0.2		0.2	0.2	0.2	0.2	0.2	0.2	0.2	0.2			0.2		
2	1	1	1	1	1	1	1		1	1	0.2		0.2			0.2	0.2	0.2	0.2	0.2	0.2	0.2			0.2		
2		1	1	1	1	1		1		1	0.2	0.2	0.2	0.2	0.2	0.2	0.2	0.2	0.2	0.2	0.2	0.2	0.2		0.2		
2		1	1	1	1	1	1			1	0.2		0.2	0.2		0.2	0.2	0.2	0.2	0.2	0.2	0.2			0.2		
2		1	1	1	1	1	1			1	0.2		0.2			0.2	0.2	0.2	0.2	0.2	0.2	0.2			0.2		
2		1	1	1	1	1		1		1	0.2	0.2	0.2			0.2	0.2	0.2	0.2	0.2	0.2	0.2	0.2		0.2		
2		1	1	1	1	1	1		1	1	0.2	0.2	0.2			0.2	0.2	0.2	0.2	0.2	0.2	0.2	0.2		0.2		
2		1	1	1	1	1	1			1	0.2	0.2	0.2			0.2	0.2	0.2	0.2	0.2	0.2	0.2	0.2		0.2		
2		1	1	1	1	1			1	1	0.2	0.2	0.2		0.2	0.2	0.2	0.2	0.2	0.2	0.2	0.2			0.2		only range of age at diagosis is stated
2		1	1	1	1	1		1		1	0.2	0.2	0.2	0.2		0.2	0.2	0.2	0.2	0.2	0.2	0.2			0.2		
