# Supplementary material for: Prenatal exposure to medication and risk of childhood cancer – a systematic review and meta-analysis
Source: BMC Cancer. 2025 Nov 21;25:1841. doi: 10.1186/s12885-025-15316-0 (PMC12667062; doi:10.1186/s12885-025-15316-0)
Supplement: Supplementary file 1 — Supplementary Material 1: Supplementary Figure 1. Prenatal exposure to analgesics and the risk of childhood cancer. Abbreviations: ES, estimate; n.a., not available. Supplementary Figure 2. Prenatal exposure to antibiotics and the risk of childhood cancer. Abbreviations: ES, estimate; 1estimates were calculated with four-square table; * calculation of crude estimates. Supplementary Figure 3. Prenatal exposure to antiemetics and the risk of childhood cancer. Abbreviations: ES, estimate; n.a., not available; 1estimates were calculated with four-square table; * calculation of crude estimates. Supplementary Figure 4. Prenatal exposure to antihistamines and the risk of childhood cancer. Abbreviations: ES, estimate; n.a., not available; 1estimates were calculated with four-square table; * calculation of crude estimates. Supplementary Figure 5. Prenatal exposure to antihypertensives and the risk of childhood cancer. Abbreviations: ES, estimate; n.a., not available. Supplementary Figure 6. Prenatal exposure to antiretroviral HIV-drugs and the risk of childhood cancer. Abbreviations: ES, estimate; n.a., not available; HIV, human immunodeficiency virus; * calculation of crude estimates. Supplementary Figure 7. Prenatal exposure to cold or cough remedies and the risk of childhood cancer. Abbreviations: ES, estimate; n.a., not available; 1estimates were calculated with four-square table; * calculation of crude estimates. Supplementary Figure 8. Prenatal exposure to diuretics and the risk of childhood cancer. Abbreviations: ES, estimate; n.a., not available; 1estimates were calculated with four-square table; *calculation of crude estimates. Supplementary Figure 9. Prenatal exposure to folic acid supplements and the risk of childhood cancer. Abbreviations: ES, estimate; n.a., not available. Supplementary Figure 10. Prenatal exposure to hormones and the risk of childhood cancer. Abbreviations: ES, estimate; n.a., not available; 1estimates were calculated with four-square table; *c [file 12885_2025_15316_MOESM1_ESM.zip › Supplementary Table 3 Exclusion of single studies_20251103_revised.docx]

**Supplementary Table 3 Exclusion of single studies**

| **A) Omitted Study Figure 2 OR (95% CI)** | | | ***I*-squared** | ***P* value** | **B) Omitted study Figure 3 OR (95% CI)** | | | ***I*-squared** | ***P* value** |
| --- | --- | --- | --- | --- | --- | --- | --- | --- | --- |
| **Antibiotics and risk of leukemia** | | | | | **Vitamin and mineral supplements and risk of leukemia** | | | | |
|  | Hjorth et al. | 1.06 (0.93, 1.22) | 0.0 % | 0.649 |  | Amigou et al. | 0.87 (0.64, 1.18) | 0.0 % | 0.709 |
|  | Kwan et al. | 1.10 (0.96, 1.26) | 0.0 % | 0.475 |  | Linabery et al. | 0.72 (0.48, 1.07) | 71.4 % | 0.062 |
|  | Momen et al. | 1.03 (0.83, 1.28) | 0.0 % | 0.507 |  | Pombo-de-Oliveira et al. | 0.62 (0.50, 0.78) | 0.0 % | 0.393 |
|  | Pombo de Oliveira et al. | 1.13 (0.98, 1.30) | 0.0 % | 0.811 | **Vitamin and mineral supplements and risk of ALL** | | | | |
|  | van Steensel-Moll et al. | 1.09 (0.95, 1.24) | 0.0 % | 0.445 |  | Amigou et al. | 0.84 (0.69, 1.03) | 63.3 % | 0.005 |
| **Antibiotics and risk of ALL** | | | | |  | Dockerty et al. | 0.80 (0.67, 0.97) | 63.4 % | 0.005 |
|  | Bonaventure et al. | 1.19 (1.09, 1.30) | 0.0 % | 0.798 |  | Linabery et al. | 0.84 (0.69, 1.02) | 65.2 % | 0.003 |
|  | Gradel et al. | 1.14 (1.03, 1.27) | 26.5 % | 0.192 |  | Milne et al. (2010) | 0.80 (0.65, 0.99) | 65.2 % | 0.003 |
|  | Hjorth et al. | 1.13 (1.02, 1.25) | 23.7 % | 0.218 |  | Mortensen et al. | 0.79 (0.65, 0.95) | 58.6 % | 0.013 |
|  | Kaatsch et al. | 1.14 (1.02, 1.26) | 26.0 % | 0.197 |  | Schüz et al. (2007) | 0.81 (0.65, 1.02) | 66.5 % | 0.002 |
|  | Kwan et al. | 1.15 (1.04, 1.27) | 23.4 % | 0.220 |  | Shaw et al. | 0.79 (0.64, 0.98) | 62.6 % | 0.006 |
|  | McKinney et al. (1999) | 1.12 (1.02, 1.23) | 14.0 % | 0.311 |  | Singer et al. | 0.86 (0.72, 1.02) | 58.9 % | 0.012 |
|  | Momen et al. | 1.13 (1.01, 1.27) | 25.6 % | 0.200 |  | Thompson et al. | 0.87 (0.73, 1.03) | 56.0 % | 0.020 |
|  | Shaw et al. | 1.13 (1.02, 1.25) | 24.4 % | 0.211 |  | Wen et al. | 0.84 (0.69, 1.03) | 64.4 % | 0.004 |
|  | Sirirungreung et al. (2023) | 1.11 (1.01, 1.23) | 16.0 % | 0.291 | **Vitamin and mineral supplements and risk of AML** | | | | |
|  | Sirirungreung et al. (2024) | 1.11 (1.01, 1.23) | 15.8 % | 0.294 |  | Amigou et al. | 1.09 (0.80, 1.47) | 0.0 % | 0.971 |
|  | Wen et al. | 1.16 (1.04, 1.28) | 20.0 % | 0.253 |  | Linabery et al. | 0.95 (0.71, 1.27) | 0.0 % | 0.458 |
|  | Ye et al. | 1.14 (1.03, 1.27) | 26.4 % | 0.193 |  | Mortensen et al. | 0.98 (0.73, 1.31) | 0.0 % | 0.413 |
| **Antibiotics and risk of AML** | | | | |  | Robison et al. | 0.97 (0.72, 1.31) | 0.0 % | 0.414 |
|  | Bonaventure et al. | 1.29 (0.69, 2.44) | 82.9 % | 0.001 |  | Schüz et al. (2007) | 0.88 (0.61, 1.26) | 0.0 % | 0.559 |
|  | Kaatsch et al. | 1.05 (0.73, 1.50) | 57.6 % | 0.070 | **Vitamin and mineral supplements and risk of CNS tumors** | | | | |
|  | Momen et al. | 1.51 (1.03, 2.23) | 60.2 % | 0.056 |  | Bunin et al. | 0.75 (0.59, 0.94) | 70.0 % | 0.001 |
|  | Sirirungreung et al. (2023) | 1.27 (0.70, 2.30) | 82.9 % | 0.001 |  | Cordier et al. | 0.83 (0.69, 1.00) | 55.8 % | 0.027 |
|  | Robison et al. | 1.28 (0.70, 2.34) | 82.9 % | 0.001 |  | Milne et al. (2012) | 0.76 (0.59, 0.98) | 71.2 % | 0.001 |
| **Antibiotics and risk of CNS tumors** | | | | |  | Mortensen et al. | 0.78 (0.61, 0.99) | 70.3 % | 0.001 |
|  | Bonaventure et al. | 1.10 (0.92, 1.33) | 44.7 % | 0.093 |  | Ortega-García et al. | 0.81 (0.66, 1.00) | 63.1 % | 0.008 |
|  | Kaatsch et al. | 1.05 (0.89, 1.24) | 31.7 % | 0.186 |  | Preston-Martin et al. | 0.78 (0.60, 1.00) | 67.9 % | 0.003 |
|  | McKinney et al. (1999) | 1.11 (0.94, 1.31) | 38.4 % | 0.136 |  | Schüz et al. (2007) | 0.73 (0.57, 0.92) | 62.9 % | 0.009 |
|  | Momen et al. | 1.09 (0.88, 1.35) | 46.3 % | 0.083 |  | Stålberg et al. | 0.73 (0.56, 0.94) | 68.2 % | 0.003 |
|  | Sirirungreung et al. (2023) | 1.03 (0.86, 1.24) | 26.6 % | 0.225 |  | Vienneau et al. | 0.77 (0.59, 0.99) | 70.4 % | 0.001 |
|  | Sirirungreung et al. (2024) | 1.07 (0.87, 1.31) | 46.8 % | 0.080 | **Vitamin and mineral supplements and risk of neuroblastoma** | | | | |
|  | Stålberg et al. | 1.18 (1.03, 1.35) | 1.5 % | 0.413 |  | Michalek et al. | 0.98 (0.53, 1.84) | 82.3 % | 0.004 |
|  | Thapa et al. | 1.08 (0.90, 1.30) | 47.2 % | 0.078 |  | Mortensen et al. | 0.77 (0.38, 1.55) | 89.5 % | 0.000 |
| **Antibiotics and risk of medulloblastoma** | | | | |  | Olshan et al. | 0.92 (0.44, 1.94) | 87.1 % | 0.000 |
|  | Bonaventure et al. | 1.63 (1.08, 2.45) | 0.0 % | 0.465 |  | Schüz et al. (2007) | 0.63 (0.44, 0.90) | 39.6 % | 0.191 |
|  | Kaatsch et al. | 1.45 (0.98, 2.15) | 0.0 % | 0.655 | **Vitamin C supplements and risk of CNS tumors** | | | | |
|  | Sirirungreung et al. (2023) | 1.77 (1.18, 2.65) | 0.0 % | 0.744 |  | Bunin et al. | 0.67 (0.41, 1.10) | 57.2 % | 0.126 |
|  | Sirirungreung et al. (2024) | 1.51 (1.04, 2.20) | 0.0 % | 0.524 |  | Milne et al. (2012) | 0.82 (0.30, 2.26) | 82.0 % | 0.018 |
| **Antibiotics and risk of germ cell tumors** | | | | |  | Preston-Martin et al. | 1.00 (0.61, 1.63) | 47.6 % | 0.167 |
|  | Momen et al. | 1.50 (1.01, 2.24) | 0.0 % | 1.000 | **Vitamin E supplements and risk of solid tumors** | | | | |
|  | Shu et al. | 1.22 (0.66, 2.23) | 30.4 % | 0.231 |  | Bunin et al. | 0.64 (0.44, 0.95) | 48.1 % | 0.165 |
|  | Sirirungreung et al. (2023) | 1.19 (0.64, 2.22) | 25.1 % | 0.248 |  | Jung et al. | 0.57 (0.34, 0.96) | 11.1 % | 0.289 |
| **Antibiotics and risk of lymphoma** | | | | |  | Preston-Martin et al. | 0.76 (0.57, 1.02) | 0.0 % | 0.644 |
|  | Bonaventure et al. | 1.29 (0.91, 1.83) | 0.0 % | 0.471 | **Vitamin A supplements and risk of solid tumors** | | | | |
|  | Kaatsch et al. | 1.10 (0.80, 1.52) | 0.0 % | 0.407 |  | Jung et al. | 0.63 (0.29, 1.35) | 74.6 % | 0.047 |
|  | McKinney et al. (1999) | 1.19 (0.87, 1.62) | 5.2 % | 0.367 |  | Milne et al. (2012) | 0.56 (0.37, 0.85) | 30.1 % | 0.232 |
|  | Momen et al. | 1.23 (0.90, 1.67) | 0.0 % | 0.442 |  | Preston-Martin et al. | 0.75 (0.55, 1.02) | 38.8 % | 0.201 |
|  | Sirirungreung et al. (2023) | 1.01 (0.70, 1.46) | 0.0 % | 0.573 | **Vitamin and mineral supplements in trimester 1 and risk of CNS tumors** | | | | |
| **Antibiotics and risk of neuroblastoma** | | | | |  | Bunin et al. | 0.80 (0.54, 1.19) | 53.0 % | 0.119 |
|  | Bonaventure et al. | 1.45 (0.90, 2.34) | 65.9 % | 0.032 |  | Milne et al. (2012) | 0.81 (0.49, 1.33) | 50.0 % | 0.135 |
|  | Cook et al. | 1.57 (1.13, 2.18) | 21.7 % | 0.280 |  | Ortega-García et al. | 0.89 (0.71, 1.12) | 0.0 % | 0.664 |
|  | Kaatsch et al. | 1.39 (0.88, 2.20) | 66.9 % | 0.028 |  | Preston-Martin et al. | 0.74 (0.49, 1.12) | 34.1 % | 0.219 |
|  | Sirirungreung et al. (2023) | 1.14 (0.83, 1.57) | 22.5 % | 0.276 | **Vitamin and mineral supplements in trimester 2/3 and risk of CNS tumors** | | | | |
|  | Thapa et al. | 1.28 (0.88, 1.85) | 59.4 % | 0.061 |  | Bunin et al. | 0.77 (0.59, 1.02) | 0.0 % | 0.392 |
| **Antibiotics and risk of Wilms tumor** | | | | |  | Milne et al. (2012) | 0.85 (0.49, 1.49) | 37.7 % | 0.201 |
|  | Bonaventure et al. | 0.93 (0.69, 1.25) | 0.0 % | 0.852 |  | Ortega-García et al. | 0.85 (0.65, 1.10) | 0.0 % | 0.518 |
|  | Kaatsch et al. | 0.91 (0.68, 1.20) | 0.0 % | 0.984 |  | Preston-Martin et al. | 0.84 (0.50, 1.41) | 40.0 % | 0.189 |
|  | Momen et al. | 0.97 (0.68, 1.40) | 0.0 % | 0.896 | **C) Omitted study Suppl. Figure 17 OR (95% CI)** | | | ***I*-squared** | ***P* value** |
|  | Sirirungreung et al. (2023) | 0.94 (0.70, 1.26) | 0.0 % | 0.857 | **Antibiotics in trimester 1 and risk of ALL** | | | | |
| **Nitrosatable antibiotics including nitrofurantoin and risk of childhood cancer** | | | | |  | Gradel et al. | 1.33 (0.87, 2.05) | 0.0 % | 0.813 |
|  | Hjorth et al. | 1.32 (1.11, 1.56) | 0.0 % | 0.834 |  | Hjorth et al. | 1.19 (0.89, 1.57) | 0.0 % | 0.644 |
|  | Momen et al. | 1.31 (1.08, 1.58) | 0.0 % | 0.901 |  | Ye et al. | 1.17 (0.84, 1.62) | 0.0 % | 0.604 |
|  | Sirirungreung et al. (2023) | 1.35 (1.07, 1.70) | 0.0 % | 0.977 | **Antibiotics in trimester 2 and risk of ALL** | | | | |
| **Penicillin and risk of leukemia** | | | | |  | Gradel et al. | 0.84 (0.55, 1.27) | 0.0 % | 0.441 |
|  | Bonaventure et al. | 1.03 (0.80, 1.34) | 35.5 % | 0.213 |  | Hjorth et al. | 1.08 (0.82, 1.43) | 0.1 % | 0.317 |
|  | Pombo-de-Oliveira et al. | 1.05 (0.89, 1.23) | 0.0 % | 0.335 |  | Ye et al. | 0.98 (0.52, 1.88) | 44.0 % | 0.181 |
|  | Sirirungreung et al. (2023) | 0.95 (0.79, 1.14) | 0.0 % | 0.601 | **Antibiotics in trimester 3 and risk of ALL** | | | | |
| **Penicillin and risk of solid tumors** | | | | |  | Gradel et al. | 1.25 (0.76, 2.04) | 32.1 % | 0.225 |
|  | Bonaventure et al. | 1.09 (0.77, 1.55) | 47.4 % | 0.168 |  | Hjorth et al. | 0.89 (0.65, 1.22) | 0.0 % | 0.599 |
|  | Cook et al. | 1.38 (1.03, 1.85) | 0.0 % | 0.404 |  | Ye et al. | 1.13 (0.58, 2.18) | 69.2 % | 0.072 |
|  | Sirirungreung et al. (2023) | 1.20 (0.63, 2.26) | 68.8 % | 0.074 | **D) Omitted study Suppl. Figure 18 OR (95% CI)** | | | ***I*-squared** | ***P* value** |
| **Amoxicillin and risk of childhood cancer** | | | | | **Acetaminophen and risk of childhood cancer** | | | | |
|  | Cook et al. | 1.02 (0.81, 1.29) | 19.1 % | 0.266 |  | Cook et al. | 0.92 (0.56, 1.53) | 43.3 % | 0.171 |
|  | Momen et al. | 0.85 (0.64, 1.13) | 0.0 % | 0.751 |  | Couto et al. | 1.10 (0.86, 1.39) | 0.0 % | 0.701 |
|  | Pombo-de-Oliveira et al. | 1.01 (0.74, 1.37) | 32.9 % | 0.222 |  | Ognjanovic et al. | 0.96 (0.57, 1.62) | 50.0 % | 0.135 |
| **Beta-lactam antibiotics excluding penicillin and risk of childhood cancer** | | | | |  | Stålberg et al. | 0.96 (0.71, 1.31) | 36.2 % | 0.209 |
|  | Cook et al. | 1.17 (0.83, 1.66) | 37.4 % | 0.206 |  |  |  |  |  |
|  | Momen et al. | 0.98 (0.47, 2.05) | 70.1 % | 0.068 |  |  |  |  |  |
|  | Sirirungreung et al. (2023) | 0.77 (0.49, 1.23) | 0.0 % | 0.447 |  |  |  |  |  |

| **D) Omitted study Suppl. Figure 18 OR (95% CI)** | | | ***I*-squared** | ***P* value** | **G) Omitted study Suppl. Figure 21 OR (95% CI)** | | | ***I*-squared** | ***P* value** |
| --- | --- | --- | --- | --- | --- | --- | --- | --- | --- |
| **Aspirin and risk of childhood cancer** | | | | | **Hormones and risk of ALL** | | | | |
|  | Cook et al. | 1.49 (0.95, 2.36) | 0.0 % | 0.820 |  | Ajrouche et al. | 1.04 (0.65, 1.66) | 73.1 % | 0.024 |
|  | Couto et al. | 1.42 (0.93, 2.18) | 0.0 % | 0.732 |  | Bonaventure et al. | 1.34 (1.02, 1.76) | 0.0 % | 0.602 |
|  | Ognjanovic et al. | 1.46 (0.95, 2.25) | 0.0 % | 0.780 |  | Shaw et al. | 1.12 (0.71, 1.75) | 77.3 % | 0.012 |
|  | Schwartzbaum et al. | 1.25 (0.75, 2.07) | 0.0 % | 0.976 |  | Shu et al. | 0.96 (0.67, 1.36) | 47.5 % | 0.149 |
| **Analgesics and risk of ALL** | | | | | **Hormones and risk of neuroblastoma** | | | | |
|  | Bonaventure et al. | 1.11 (0.90, 1.38) | 0.0 % | 0.605 |  | Cook et al. | 1.54 (1.04, 2.30) | 13.9 % | 0.325 |
|  | Couto et al. | 1.14 (0.95, 1.37) | 0.0 % | 0.626 |  | Kramer et al. | 1.41 (0.98, 2.04) | 15.3 % | 0.317 |
|  | McKinney et al. | 1.17 (0.99, 1.39) | 0.0 % | 0.516 |  | Michalek et al. | 1.27 (0.90, 1.79) | 0.0 % | 0.817 |
|  | Ognjanovic et al. | 1.21 (1.01, 1.46) | 0.0 % | 0.586 |  | Olshan et al. | 1.57 (1.10, 2.25) | 0.0 % | 0.432 |
|  | Schüz et al. (2007) | 1.25 (1.04, 1.50) | 0.0 % | 0.859 |  | Schüz et al. (2001) | 1.42 (0.98, 2.06) | 17.0 % | 0.307 |
|  | Shaw et al. | 1.17 (0.98, 1.38) | 0.0 % | 0.603 |  | Schwartzbaum et al. | 1.54 (1.03, 2.29) | 14.6 % | 0.322 |
|  | Wen et al. | 1.18 (0.99, 1.41) | 0.0 % | 0.512 | **Oral contraceptives and risk of ALL** | | | | |
| **Analgesics and risk of AML** | | | | |  | Ajrouche et al. | 1.28 (0.98, 1.66) | 0.0 % | 0.532 |
|  | Bonaventure et al. | 0.90 (0.63, 1.28) | 0.0 % | 0.672 |  | Hargreave et al. | 1.29 (1.02, 1.63) | 0.0 % | 0.534 |
|  | Couto et al. | 0.82 (0.57, 1.16) | 0.0 % | 0.836 |  | Kwan et al. | 1.41 (1.08, 1.85) | 0.0 % | 0.921 |
|  | Ognjanovic et al. | 0.95 (0.61, 1.48) | 0.0 % | 0.718 |  | Shaw et al. | 1.26 (1.00, 1.59) | 0.0 % | 0.610 |
|  | Schüz et al. (2007) | 0.85 (0.60, 1.19) | 0.0 % | 0.670 |  | Shu et al. | 1.19 (0.90, 1.57) | 0.0 % | 0.729 |
| **Analgesics and risk of CNS tumors** | | | | | **Oral contraceptives and risk of neuroblastoma** | | | | |
|  | Bonaventure et al. | 0.98 (0.73, 1.31) | 0.0 % | 0.667 |  | Cook et al. | 2.16 (0.40, 11.86) | 79.6 % | 0.027 |
|  | Cheng et al. | 1.09 (0.82, 1.44) | 0.0 % | 0.468 |  | Olshan et al. | 2.33 (0.51, 10.58) | 75.6 % | 0.043 |
|  | McCredie et al. | 1.01 (0.77, 1.33) | 0.0 % | 0.696 |  | Schüz et al. (2001) | 1.11 (0.68, 1.79) | 0.0 % | 0.712 |
|  | McKinney et al. | 1.07 (0.81, 1.41) | 0.0 % | 0.433 | **H) Omitted study Suppl. Figure 22 OR (95% CI)** | | | ***I*-squared** | ***P* value** |
|  | Schüz et al. (2007) | 1.08 (0.79, 1.48) | 0.0 % | 0.421 | **Antihistamines and risk of CNS tumors** | | | | |
|  | Stålberg et al. | 1.15 (0.82, 1.60) | 0.0 % | 0.520 |  | Cheng et al. | 0.94 (0.67, 1.32) | 0.0 % | 0.494 |
| **Analgesics and risk of neuroblastoma** | | | | |  | Cordier et al. | 0.93 (0.68, 1.27) | 0.0 % | 0.962 |
|  | Bonaventure et al. | 1.28 (0.90, 1.82) | 41.1 % | 0.183 |  | Kuijten et al. | 0.98 (0.70, 1.37) | 0.0 % | 0.469 |
|  | Cook et al. | 1.63 (1.12, 2.37) | 19.3 % | 0.290 |  | McCredie et al. | 0.97 (0.71, 1.34) | 0.0 % | 0.470 |
|  | Schüz et al. (2007) | 1.52 (0.99, 2.31) | 58.3 % | 0.091 |  | Stålberg et al. | 1.11 (0.65, 1.88) | 0.0 % | 0.535 |
|  | Schwartzbaum et al. | 1.26 (0.88, 1.78) | 33.3 % | 0.223 | **Antihistamines and risk of leukemia** | | | | |
| **Analgesics and risk of lymphoma** | | | | |  | Robison et al. | 1.44 (0.90, 2.30) | 0.0 % | 0.775 |
|  | Bonaventure et al. | 0.92 (0.47, 1.81) | 0.0 % | 0.462 |  | Shaw et al. | 1.46 (0.96, 2.23) | 0.0 % | 0.754 |
|  | McKinney et al. | 2.29 (0.50, 10.57) | 85.9 % | 0.008 |  | Wen et al. | 1.64 (0.86, 3.13) | 0.0 % | 0.948 |
|  | Schüz et al. (2007) | 1.85 (0.22, 15.32) | 84.9 % | 0.010 | **I) Omitted study Suppl. Figure 23 OR (95% CI)** | | | ***I*-squared** | ***P* value** |
| **E) Omitted study Suppl. Figure 19 OR (95% CI)** | | | ***I*-squared** | ***P* value** | **Diuretics and risk of CNS tumors** | | | | |
| **Antiemetics and risk of leukemia** | | | | |  | Askins et al. | 1.17 (0.82, 1.69) | 0.0 % | 0.432 |
|  | Kwan et al. | 1.65 (1.15, 2.36) | 0.0 % | 0.975 |  | Kuijten et al. | 1.34 (0.95, 1.90) | 0.0 % | 0.454 |
|  | Orimoloye et al. | 1.46 (1.04, 2.05) | 0.0 % | 0.644 |  | McCredie et al. | 1.33 (0.91, 1.94) | 8.7 % | 0.349 |
|  | Pombo-de-Oliveira et al. | 1.43 (0.99, 2.05) | 0.0 % | 0.672 |  | Schüz et al. (2007) | 1.19 (0.71, 1.98) | 21.0 % | 0.284 |
|  | Robison et al. | 1.39 (0.95, 2.02) | 0.0 % | 0.726 |  | Stålberg et al. | 1.30 (0.67, 2.53) | 26.2 % | 0.255 |
|  | Schüz et al. (2007) | 1.50 (1.07, 2.11) | 0.0 % | 0.637 | **J) Omitted study Suppl. Figure 24 OR (95% CI)** | | | ***I*-squared** | ***P* value** |
| **Antiemetics and risk of ALL** | | | | | **Folic acid supplements and risk of CNS tumors** | | | | |
|  | Kwan et al. | 1.18 (0.95, 1.46) | 0.0 % | 0.691 |  | Bunin et al. | 0.82 (0.64, 1.06) | 55.4 % | 0.062 |
|  | Orimoloye et al. | 1.27 (1.01, 1.59) | 0.0 % | 0.929 |  | Milne et al. (2012) | 0.83 (0.61, 1.13) | 55.6 % | 0.061 |
|  | Schüz et al. (2007) | 1.20 (0.95, 1.51) | 0.0 % | 0.584 |  | Mortensen et al. | 0.78 (0.61, 1.00) | 42.3 % | 0.139 |
|  | Shaw et al. | 1.19 (0.94, 1.51) | 0.0 % | 0.582 |  | Ortega-García et al. | 0.79 (0.57, 1.09) | 50.5 % | 0.088 |
|  | Wen et al. | 1.12 (0.88, 1.43) | 0.0 % | 0.775 |  | Preston-Martin et al. | 0.92 (0.80, 1.07) | 0.0 % | 0.459 |
| **Antiemetics and risk of CNS tumors** | | | | |  | Stålberg et al. | 0.87 (0.68, 1.10) | 48.5 % | 0.101 |
|  | Kuijten et al. | 0.94 (0.71, 1.25) | 0.0 % | 0.679 | **Folic acid supplements in trimester 1 and risk of leukemia** | | | | |
|  | Orimoloye et al. | 1.25 (0.78, 1.98) | 62.0 % | 0.072 |  | Ajrouche et al. | 0.61 (0.16, 2.35) | 94.9 % | 0.000 |
|  | Schüz et al. (2007) | 1.13 (0.67, 1.90) | 69.9 % | 0.036 |  | Amigou et al. | 1.14 (0.95, 1.37) | 0.0 % | 0.678 |
|  | Stålberg et al. | 1.24 (0.76, 2.04) | 62.0 % | 0.072 |  | Milne et al. (2010) | 0.59 (0.16, 2.10) | 94.3 % | 0.000 |
| **Antiemetics and risk of lymphoma** | | | | | **Folic acid supplements in trimester 2/3 and risk of leukemia** | | | | |
|  | McKinney et al. (1999) | 1.16 (0.69, 1.95) | 0.0 % | 0.879 |  | Ajrouche et al. | 0.79 (0.63, 0.99) | 0.0 % | 0.328 |
|  | Orimoloye et al. | 1.36 (0.63, 2.95) | 0.0 % | 0.644 |  | Amigou et al. | 0.95 (0.72, 1.26) | 59.0 % | 0.118 |
|  | Schüz et al. (2007) | 1.22 (0.67, 2.20) | 0.0 % | 0.555 |  | Milne et al. (2010) | 0.87 (0.48, 1.55) | 69.7 % | 0.069 |
| **Antiemetics and risk of neuroblastoma** | | | | | **Folic acid supplements and risk of leukemia** | | | | |
|  | Orimoloye et al. | 1.16 (0.76, 1.77) | 0.0 % | 0.811 |  | Ajrouche et al. | 0.69 (0.23, 2.05) | 91.9 % | 0.000 |
|  | Schüz et al. (2007) | 1.27 (0.83, 1.93) | 0.0 % | 0.686 |  | Amigou et al. | 1.12 (0.89, 1.41) | 0.0 % | 0.725 |
|  | Schwartzbaum et al. | 1.25 (0.72, 2.18) | 0.0 % | 0.591 |  | Pombo-de-Oliveira et al. | 0.67 (0.25, 1.80) | 95.3 % | 0.000 |
| **F) Omitted study Suppl. Figure 20 OR (95% CI)** | | | ***I*-squared** | ***P* value** | **Folic acid supplements and risk of ALL** | | | | |
| **Antihypertensives and risk of ALL** | | | | |  | Ajrouche et al. | 0.77 (0.44, 1.36) | 88.4 % | 0.000 |
|  | Askins et al. | 1.96 (1.10, 3.51) | 0.0 % | 0.944 |  | Amigou et al. | 1.01 (0.75, 1.37) | 63.9 % | 0.026 |
|  | Schüz et al. (2007) | 1.51 (0.90, 2.54) | 0.0 % | 0.649 |  | Dockerty et al. | 0.79 (0.50, 1.27) | 89.3 % | 0.000 |
|  | Wen et al. | 1.64 (1.05, 2.56) | 0.0 % | 0.461 |  | Milne et al. (2010) | 0.76 (0.44, 1.30) | 87.6 % | 0.000 |
| **Antihypertensives and risk of solid tumors** | | | | |  | Mortensen et al. | 0.75 (0.45, 1.24) | 88.2 % | 0.000 |
|  | Askins et al. | 2.30 (1.29, 4.08) | 0.0 % | 0.722 |  | Thompson et al. | 0.93 (0.60, 1.45) | 86.8 % | 0.000 |
|  | Schüz et al. (2007) | 1.58 (0.98, 2.56) | 0.0 % | 0.545 | **Folic acid supplements and risk of AML** | | | | |
|  | Schwartzbaum et al. | 1.83 (1.08, 3.09) | 10.2 % | 0.342 |  | Ajrouche et al. | 0.44 (0.21, 0.91) | 0.0 % | 0.371 |
|  | Shu et al. | 1.81 (1.14, 2.88) | 4.7 % | 0.369 |  | Amigou et al. | 0.92 (0.53, 1.60) | 9.7 % | 0.293 |
|  | Stålberg et al. | 1.54 (0.93, 2.55) | 0.0 % | 0.524 |  | Mortensen et al. | 0.63 (0.18, 2.21) | 75.8 % | 0.042 |
| **G) Omitted study Suppl. Figure 21 OR (95% CI)** | | | ***I*-squared** | ***P* value** | **K) Omitted study Suppl. Figure 25 OR (95% CI)** | | | ***I*-squared** | ***P* value** |
| **Hormones and risk of leukemia** | | | | | **Nervous system medication and risk of leukemia** | | | | |
|  | Ajrouche et al. | 1.61 (1.07, 2.42) | 67.7 % | 0.008 |  | Bonaventure et al. | 1.16 (0.17, 7.66) | 84.0 % | 0.012 |
|  | Bonaventure et al. | 1.56 (1.05, 2.32) | 68.5 % | 0.007 |  | Robison et al. | 1.60 (0.56, 4.56) | 74.6 % | 0.047 |
|  | Hargreave et al. | 1.47 (1.02, 2.13) | 66.7 % | 0.010 |  | van Steensel-Moll et al. | 0.77 (0.36, 1.65) | 35.5 % | 0.213 |
|  | Kwan et al. | 1.65 (1.15, 2.37) | 59.0 % | 0.032 | **Nervous system medication and risk of ALL** | | | | |
|  | Pombo-de-Oliveira et al. | 1.27 (1.04, 1.54) | 0.0 % | 0.436 |  | Bonaventure et al. | 1.72 (0.91, 3.25) | 27.5 % | 0.247 |
|  | Robison et al. | 1.59 (1.06, 2.37) | 68.4 % | 0.007 |  | McKinney et al. (1999) | 1.34 (0.88, 2.05) | 23.1 % | 0.272 |
|  | van Steensel-Moll et al. | 1.46 (1.01, 2.09) | 65.7 % | 0.012 |  | Platamone et al. | 1.62 (0.97, 2.69) | 34.9 % | 0.203 |
|  |  |  |  |  |  | Shaw et al. | 1.28 (1.03, 1.60) | 0.0 % | 0.690 |
|  |  |  |  |  |  | Wen et al. | 1.49 (0.95, 2.35) | 39.7 % | 0.174 |

| **K) Omitted study Suppl. Figure 25 OR (95% CI)** | | | ***I*-squared** | ***P* value** |  |  |  |  |  |
| --- | --- | --- | --- | --- | --- | --- | --- | --- | --- |
| **Nervous system medication and risk of CNS tumors** | | | | |  |  |  |  |  |
|  | Cheng et al. | 1.26 (0.85, 1.88) | 0.0 % | 0.575 |  |  |  |  |  |
|  | Gold et al. | 1.13 (0.76, 1.68) | 0.0 % | 0.660 |  |  |  |  |  |
|  | Goldhaber et al. | 1.32 (0.83, 2.10) | 0.0 % | 0.525 |  |  |  |  |  |
|  | Kuijten et al. | 1.22 (0.81, 1.84) | 0.0 % | 0.454 |  |  |  |  |  |
|  | McCredie et al. | 1.27 (0.85, 1.91) | 0.0 % | 0.557 |  |  |  |  |  |
|  | McKinney et al. (1999) | 1.17 (0.76, 1.79) | 0.0 % | 0.463 |  |  |  |  |  |
|  | Platamone et al. | 1.08 (0.71, 1.64) | 0.0 % | 0.678 |  |  |  |  |  |

Abbreviations: ES, estimate; Suppl., Supplementary; Publication years are provided for authors with multiple studies included in the analysis
