# Supplementary material for: Prenatal exposure to medication and risk of childhood cancer – a systematic review and meta-analysis
Source: BMC Cancer. 2025 Nov 21;25:1841. doi: 10.1186/s12885-025-15316-0 (PMC12667062; doi:10.1186/s12885-025-15316-0)
Supplement: Supplementary file 1 — Supplementary Material 1: Supplementary Figure 1. Prenatal exposure to analgesics and the risk of childhood cancer. Abbreviations: ES, estimate; n.a., not available. Supplementary Figure 2. Prenatal exposure to antibiotics and the risk of childhood cancer. Abbreviations: ES, estimate; 1estimates were calculated with four-square table; * calculation of crude estimates. Supplementary Figure 3. Prenatal exposure to antiemetics and the risk of childhood cancer. Abbreviations: ES, estimate; n.a., not available; 1estimates were calculated with four-square table; * calculation of crude estimates. Supplementary Figure 4. Prenatal exposure to antihistamines and the risk of childhood cancer. Abbreviations: ES, estimate; n.a., not available; 1estimates were calculated with four-square table; * calculation of crude estimates. Supplementary Figure 5. Prenatal exposure to antihypertensives and the risk of childhood cancer. Abbreviations: ES, estimate; n.a., not available. Supplementary Figure 6. Prenatal exposure to antiretroviral HIV-drugs and the risk of childhood cancer. Abbreviations: ES, estimate; n.a., not available; HIV, human immunodeficiency virus; * calculation of crude estimates. Supplementary Figure 7. Prenatal exposure to cold or cough remedies and the risk of childhood cancer. Abbreviations: ES, estimate; n.a., not available; 1estimates were calculated with four-square table; * calculation of crude estimates. Supplementary Figure 8. Prenatal exposure to diuretics and the risk of childhood cancer. Abbreviations: ES, estimate; n.a., not available; 1estimates were calculated with four-square table; *calculation of crude estimates. Supplementary Figure 9. Prenatal exposure to folic acid supplements and the risk of childhood cancer. Abbreviations: ES, estimate; n.a., not available. Supplementary Figure 10. Prenatal exposure to hormones and the risk of childhood cancer. Abbreviations: ES, estimate; n.a., not available; 1estimates were calculated with four-square table; *c [file 12885_2025_15316_MOESM1_ESM.zip › Supplementary Table 4 Stratification by study period_revised.docx]

| **Model** | **Early (ES (95%CI))** | **n** | **I^2^** | **P value** | **Late (ES (95%CI))** | **n** | **I^2^** | **P value** |
| --- | --- | --- | --- | --- | --- | --- | --- | --- |
| Acetaminophen and risk of childhood cancer | 1.10 (0.80, 1.51) | 1 |  |  | 0.92 (0.56, 1.53) | 3 | 43.3 % | 0.171 |
| Aspirin and risk of childhood cancer | 1.48 (0.92, 2.40) | 2 | 0.0 % | 0.489 | 1.28 (0.65, 2.51) | 2 | 0.0 % | 0.857 |
| Analgesics and risk of ALL | 1.03 (0.75, 1.41) | 4 | 0.0 % | 0.559 | 1.24 (1.02, 1.51) | 3 | 0.0 % | 0.524 |
| Analgesics and risk of AML | 1.04 (0.43, 2.53) | 1 |  |  | 0.85 (0.60, 1.19) | 3 | 0.0 % | 0.670 |
| Analgesics and risk of CNS tumors | 1.10 (0.71, 1.68) | 3 | 0.0 % | 0.407 | 1.04 (0.72, 1.48) | 3 | 5.4 % | 0.348 |
| Analgesics and risk of neuroblastoma | 1.28 (0.90, 1.82) | 3 | 41.1 % | 0.183 | 1.99 (1.07, 3.70) | 1 |  |  |
| Analgesics and risk of lymphoma | 0.92 (0.47, 1.81) | 2 | 0.0 % | 0.462 | 5.05 (2.16, 11.81) | 1 |  |  |
| Antibiotics and risk of leukemia | 0.94 (0.73, 1.21) | 3 | 0.0 % | 0.823 | 1.15 (0.98, 1.34) | 2 | 0.0 % | 0.441 |
| Antibiotics and risk of ALL | 1.11 (0.90, 1.38) | 4 | 17.8 % | 0.302 | 1.15 (1.02, 1.29) | 8 | 28.3 % | 0.202 |
| Antibiotics and risk of AML | 1.26 (0.79, 2.01) | 1 |  |  | 1.28 (0.70, 2.34) | 4 | 82.9 % | 0.001 |
| Antibiotics and risk of CNS tumors | 0.76 (0.37, 1.56) | 2 | 0.0 % | 0.452 | 1.11 (0.93, 1.33) | 6 | 48.6 % | 0.084 |
| Antibiotics and risk of germ cell tumors | 1.50 (0.82, 2.76) | 1 |  |  | 1.22 (0.66, 2.23) | 2 | 30.4 % | 0.231 |
| Antibiotics and risk of lymphoma | 0.75 (0.20, 2.82) | 1 |  |  | 1.19 (0.87, 1.62) | 4 | 5.2 % | 0.367 |
| Antibiotics and risk of neuroblastoma | 1.35 (0.49, 3.72) | 2 | 70.0 % | 0.068 | 1.49 (1.04, 2.13) | 3 | 31.6 % | 0.232 |
| Penicillin and risk of leukemia | 0.88 (0.62, 1.24) | 1 |  |  | 1.05 (0.89, 1.23) | 2 | 0.0 % | 0.335 |
| Penicillin and risk of solid tumors | 0.90 (0.61, 1.32) | 1 |  |  | 1.38 (1.03, 1.85) | 2 | 0.0 % | 0.404 |
| Amoxicillin and risk of childhood cancer | 0.85 (0.64, 1.13) | 2 | 0.0 % | 0.751 | 1.12 (0.87, 1.44) | 1 |  |  |
| Beta-lactam antibiotics and risk of childhood cancer | 0.60 (0.27, 1.34) | 1 |  |  | 1.17 (0.83, 1.66) | 2 | 37.4 % | 0.206 |
| Antiemetics and risk of leukemia | 1.46 (1.04, 2.05) | 4 | 0.0 % | 0.644 | 1.65 (0.70, 3.91) | 1 |  |  |
| Antiemetics and risk of ALL | 1.27 (1.01, 1.59) | 4 | 0.0 % | 0.929 | 0.90 (0.55, 1.48) | 1 |  |  |
| Antiemetics and risk of CNS tumors | 1.51 (0.88, 2.60) | 2 | 51.6 % | 0.150 | 0.88 (0.63, 1.22) | 2 | 0.0 % | 0.841 |
| Antiemetics and risk lymphoma | 1.36 (0.63, 2.95) | 2 | 0.0 % | 0.644 | 1.13 (0.60, 2.14) | 1 |  |  |
| Antiemetics and risk of neuroblastoma | 1.16 (0.76, 1.77) | 2 | 0.0 % | 0.811 | 1.45 (0.67, 3.13) | 1 |  |  |
| Antihistamines and risk of CNS tumors | 1.12 (0.53, 2.38) | 3 | 8.1 % | 0.337 | 0.94 (0.66, 1.33) | 2 | 0.0 % | 0.614 |
| Antihypertensives and risk of ALL | 1.96 (1.10, 3.51) | 2 | 0.0 % | 0.944 | 1.42 (0.79, 2.55) | 1 |  |  |
| Antihypertensives and risk of solid tumors | 2.06 (0.98, 4.33) | 3 | 0.0 % | 0.570 | 1.70 (0.77, 3.77) | 2 | 49.6 % | 0.159 |
| Diuretics and risk of CNS tumors | 0.98 (0.41, 2.31) | 3 | 26.3 % | 0.257 | 1.40 (0.84, 2.34) | 2 | 24.6 % | 0.249 |
| Folic acid supplements and risk of leukemia | 1.22 (0.73, 2.04) | 1 |  |  | 0.67 (0.25, 1.80) | 2 | 95.3 % | 0.000 |
| Folic acid supplements and risk of ALL | 0.64 (0.24, 1.71) | 2 | 72.0 % | 0.059 | 0.91 (0.56, 1.49) | 4 | 90.1 % | 0.000 |
| Folic acid supplements and risk of CNS tumors | 0.65 (0.31, 1.33) | 2 | 44.7 % | 0.179 | 0.92 (0.77, 1.09) | 4 | 14.7 % | 0.319 |
| Hormones and risk of leukemia | 1.78 (0.93, 3.37) | 4 | 79.7 % | 0.002 | 1.35 (1.02, 1.78) | 3 | 0.0 % | 0.583 |
| Hormones and risk of ALL | 1.36 (0.96, 1.91) | 2 | 0.0 % | 0.319 | 0.97 (0.58, 1.61) | 2 | 73.0 % | 0.054 |
| Oral contraceptives and risk of ALL | 1.29 (0.96, 1.72) | 3 | 8.6 % | 0.335 | 1.28 (0.86, 1.91) | 2 | 0.0 % | 0.896 |
| Nervous system medication and risk of leukemia | 1.16 (0.17, 7.66) | 2 | 84.0 % | 0.012 | 0.99 (0.56, 1.75) | 1 |  |  |
| Nervous system medication and risk of ALL | 2.25 (1.14, 4.45) | 3 | 3.2 % | 0.356 | 1.24 (0.98, 1.57) | 2 | 0.0 % | 0.646 |
| Nervous system medication and risk of CNS tumors | 1.13 (0.73, 1.76) | 5 | 0.0 % | 0.647 | 1.29 (0.38, 4.35) | 2 | 48.1 % | 0.165 |
| Vitamin and mineral supplements and risk of leukemia | 0.90 (0.63, 1.28) | 1 |  |  | 0.62 (0.50, 0.78) | 2 | 0.0 % | 0.393 |
| Vitamin and mineral supplements and risk of ALL | 0.82 (0.64, 1.06) | 5 | 64.6 % | 0.024 | 0.81 (0.58, 1.13) | 5 | 68.6 % | 0.013 |
| Vitamin and mineral supplements and risk of AML | 1.09 (0.76, 1.56) | 2 | 0.0 % | 0.763 | 0.83 (0.54, 1.28) | 3 | 0.0 % | 0.396 |
| Vitamin and mineral supplements and risk of CNS tumors | 0.76 (0.48, 1.20) | 4 | 80.7 % | 0.001 | 0.77 (0.60, 0.99) | 5 | 53.8 % | 0.070 |
| Vitamin and mineral supplements and risk of neuroblastoma | 0.77 (0.38, 1.55) | 3 | 89.5 % | 0.000 | 1.05 (0.53, 2.07) | 1 |  |  |
| Vitamin C supplements and risk of CNS tumors | 0.82 (0.30, 2.26) | 2 | 82.0 % | 0.018 | 0.83 (0.59, 1.17) | 1 |  |  |
| Vitamin E supplements and risk of solid tumors | 0.57 (0.34, 0.96) | 2 | 11.1 % | 0.289 | 0.75 (0.56, 1.01) | 1 |  |  |
| Vitamin and mineral supplements in trimester 1 and risk of CNS tumors | 0.57 (0.19, 1.70) | 2 | 65.7 % | 0.088 | 0.89 (0.70, 1.14) | 2 | 0.0 % | 0.366 |
| Vitamin and mineral supplements in trimester 2/3 and risk of CNS tumors | 0.92 (0.63, 1.36) | 2 | 0.0 % | 0.324 | 0.55 (0.16, 1.91) | 2 | 45.8 % | 0.174 |

**Supplementary Table 4 Stratification by study period**

< 2010: early study, ≥ 2010 late study

Abbreviations: CI, confidence interval; OR, odds ratio; ALL, acute lymphocytic leukemia; AML, acute myeloid leukemia; CNS, central nervous system
