# Supplementary material for: Prenatal exposure to medication and risk of childhood cancer – a systematic review and meta-analysis
Source: BMC Cancer. 2025 Nov 21;25:1841. doi: 10.1186/s12885-025-15316-0 (PMC12667062; doi:10.1186/s12885-025-15316-0)
Supplement: Supplementary file 1 — Supplementary Material 1: Supplementary Figure 1. Prenatal exposure to analgesics and the risk of childhood cancer. Abbreviations: ES, estimate; n.a., not available. Supplementary Figure 2. Prenatal exposure to antibiotics and the risk of childhood cancer. Abbreviations: ES, estimate; 1estimates were calculated with four-square table; * calculation of crude estimates. Supplementary Figure 3. Prenatal exposure to antiemetics and the risk of childhood cancer. Abbreviations: ES, estimate; n.a., not available; 1estimates were calculated with four-square table; * calculation of crude estimates. Supplementary Figure 4. Prenatal exposure to antihistamines and the risk of childhood cancer. Abbreviations: ES, estimate; n.a., not available; 1estimates were calculated with four-square table; * calculation of crude estimates. Supplementary Figure 5. Prenatal exposure to antihypertensives and the risk of childhood cancer. Abbreviations: ES, estimate; n.a., not available. Supplementary Figure 6. Prenatal exposure to antiretroviral HIV-drugs and the risk of childhood cancer. Abbreviations: ES, estimate; n.a., not available; HIV, human immunodeficiency virus; * calculation of crude estimates. Supplementary Figure 7. Prenatal exposure to cold or cough remedies and the risk of childhood cancer. Abbreviations: ES, estimate; n.a., not available; 1estimates were calculated with four-square table; * calculation of crude estimates. Supplementary Figure 8. Prenatal exposure to diuretics and the risk of childhood cancer. Abbreviations: ES, estimate; n.a., not available; 1estimates were calculated with four-square table; *calculation of crude estimates. Supplementary Figure 9. Prenatal exposure to folic acid supplements and the risk of childhood cancer. Abbreviations: ES, estimate; n.a., not available. Supplementary Figure 10. Prenatal exposure to hormones and the risk of childhood cancer. Abbreviations: ES, estimate; n.a., not available; 1estimates were calculated with four-square table; *c [file 12885_2025_15316_MOESM1_ESM.zip › Supplementary Table 5 Stratification by study region_revised.docx]

| **Model** | **AM (ES (95%CI))** | **n** | **I^2^** | **P value** | **EU (ES (95%CI))** | **n** | **I^2^** | **P value** | **AS (ES (95%CI))** | **n** | **I^2^** | **P value** | **OC (ES (95%CI))** | **n** | **I^2^** | **P value** |
| --- | --- | --- | --- | --- | --- | --- | --- | --- | --- | --- | --- | --- | --- | --- | --- | --- |
| Acetaminophen and risk of childhood cancer | 0.96 (0.71, 1.31) | 3 | 36.2 % | 0.209 | 1.70 (0.57, 5.10) | 1 |  |  |  |  |  |  |  |  |  |  |
| Analgesics and risk of ALL | 1.20 (0.93, 1.56) | 4 | 0.0 % | 0.617 | 1.14 (0.88, 1.48) | 3 | 18.6 % | 0.293 |  |  |  |  |  |  |  |  |
| Analgesics and risk of AML | 0.87 (0.59, 1.28) | 2 | 0.0 % | 0.413 | 0.86 (0.49, 1.50) | 2 | 0.0 % | 0.577 |  |  |  |  |  |  |  |  |
| Analgesics and risk of CNS tumors | 0.80 (0.32, 2.00) | 1 |  |  | 1.03 (0.77, 1.38) | 4 | 0.0 % | 0.585 |  |  |  |  | 2.20 (0.72, 6.76) | 1 |  |  |
| Analgesics and risk of neuroblastoma | 1.39 (0.82, 2.36) | 2 | 68.0 % | 0.077 | 1.46 (0.79, 2.71) | 2 | 48.1 % | 0.165 |  |  |  |  |  |  |  |  |
| Antibiotics and risk of leukemia | 0.93 (0.71, 1.21) | 2 | 0.0 % | 0.643 | 1.14 (0.98, 1.33) | 3 | 0.0 % | 0.739 |  |  |  |  |  |  |  |  |
| Antibiotics and risk of ALL | 1.05 (0.88, 1.25) | 4 | 0.0 % | 0.754 | 1.15 (1.00, 1.34) | 7 | 41.3 % | 0.115 | 1.30 (1.04, 1.63) | 1 |  |  |  |  |  |  |
| Antibiotics and risk of AML | 1.26 (0.79, 2.01) | 1 |  |  | 1.28 (0.70, 2.34) | 4 | 82.9 % | 0.001 |  |  |  |  |  |  |  |  |
| Antibiotics and risk of CNS tumors | 1.23 (0.29, 5.21) | 1 |  |  | 1.06 (0.86, 1.32) | 6 | 55.6 % | 0.046 | 1.16 (0.84, 1.60) | 1 |  |  |  |  |  |  |
| Antibiotics and risk of germ cell tumors | 1.50 (0.82, 2.76) | 1 |  |  | 1.22 (0.66, 2.23) | 2 | 30.4 % | 0.231 |  |  |  |  |  |  |  |  |
| Antibiotics and risk of medulloblastoma |  |  |  |  | 1.51 (1.04, 2.20) | 3 | 0.0 % | 0.524 | 1.96 (0.85, 4.51) | 1 |  |  |  |  |  |  |
| Antibiotics and risk of neuroblastoma | 1.35 (0.49, 3.72) | 2 | 70.0 % | 0.068 | 1.49 (1.04, 2.13) | 3 | 31.6 % | 0.232 |  |  |  |  |  |  |  |  |
| Penicillin and risk of leukemia | 0.88 (0.62, 1.24) | 1 |  |  | 0.98 (0.79, 1.21) | 1 |  |  | 1.15 (0.90, 1.47) | 1 |  |  |  |  |  |  |
| Penicillin and risk of solid tumors | 0.90 (0.61, 1.32) | 1 |  |  | 1.73 (0.95, 3.16) | 1 |  |  | 1.29 (0.92, 1.80) | 1 |  |  |  |  |  |  |
| Amoxicillin and risk of childhood cancer | 0.85 (0.64, 1.13) | 2 | 0.0 % | 0.751 | 1.12 (0.87, 1.44) | 1 |  |  |  |  |  |  |  |  |  |  |
| Beta-lactam antibiotics and risk of childhood cancer | 0.60 (0.27, 1.34) | 1 |  |  | 0.88 (0.50, 1.56) | 1 |  |  | 1.31 (1.04, 1.65) | 1 |  |  |  |  |  |  |
| Antibiotics in trimester 1 and risk of ALL | 1.30 (0.80, 2.11) | 1 |  |  | 1.17 (0.84, 1.62) | 2 | 0.0 % | 0.604 |  |  |  |  |  |  |  |  |
| Antibiotics in trimester 2 and risk of ALL | 0.90 (0.57, 1.42) | 1 |  |  | 0.98 (0.52, 1.88) | 2 | 44.0 % | 0.181 |  |  |  |  |  |  |  |  |
| Antibiotics in trimester 3 and risk of ALL | 1.00 (0.59, 1.68) | 1 |  |  | 1.13 (0.58, 2.18) | 2 | 69.2 % | 0.072 |  |  |  |  |  |  |  |  |
| Antiemetics and risk of leukemia | 1.47 (1.02, 2.14) | 3 | 0.0 % | 0.439 | 1.51 (0.81, 2.79) | 2 | 0.0 % | 0.767 |  |  |  |  |  |  |  |  |
| Antiemetics and risk of ALL | 1.30 (1.00, 1.70) | 3 | 0.0 % | 0.867 | 1.04 (0.75, 1.45) | 2 | 0.0 % | 0.437 |  |  |  |  |  |  |  |  |
| Antiemetics and risk of CNS tumors | 2.00 (1.17, 3.42) | 1 |  |  | 0.94 (0.71, 1.75) | 3 | 0.0 % | 0.679 |  |  |  |  |  |  |  |  |
| Antiemetics and risk of neuroblastoma | 1.20 (0.73, 1.98) | 1 |  |  | 1.25 (0.72, 2.18) | 2 | 0.0 % | 0.591 |  |  |  |  |  |  |  |  |
| Antihistamines and risk of CNS tumors | 1.01 (0.57, 1.80) | 2 | 0.0 % | 0.689 | 1.41 (0.37, 5.29) | 2 | 57.4 % | 0.126 |  |  |  |  | 0.80 (0.13, 5.06) | 1 |  |  |
| Antihypertensives and risk of ALL | 1.90 (0.63, 5.75) | 1 |  |  | 1.64 (1.05, 2.56) | 2 | 0.0 % | 0.461 |  |  |  |  |  |  |  |  |
| Antihypertensives and risk of solid tumors | 1.47 (0.55, 3.97) | 2 | 0.0 % | 0.724 | 1.96 (1.04, 3.69) | 3 | 35.4 % | 0.213 |  |  |  |  |  |  |  |  |
| Diuretics and risk of CNS tumors | 0.50 (0.11, 2.18) | 1 |  |  | 1.39 (0.98, 1.98) | 3 | 0.0 % | 0.465 |  |  |  |  | 0.50 (0.08, 3.32) | 1 |  |  |
| Folic acid supplements in trimester 1 and risk of leukemia |  |  |  |  | 0.59 (0.16, 2.10) | 2 | 94.3 % | 0.000 |  |  |  |  | 1.19 (0.91, 1.56) | 1 |  |  |
| Folic acid supplements in trimester 2/3 and risk of leukemia |  |  |  |  | 0.87 (0.48, 1.55) | 2 | 69.7 % | 0.069 |  |  |  |  | 0.83 (0.65, 1.06) | 1 |  |  |
| Folic acid supplements and risk of leukemia | 1.22 (0.73, 2.04) | 1 |  |  | 0.67 (0.25, 1.80) | 2 | 95.3 % | 0.000 |  |  |  |  |  |  |  |  |
| Folic acid supplements and risk of ALL |  |  |  |  | 0.83 (0.41, 1.66) | 3 | 92.4 % | 0.000 |  |  |  |  | 0.82 (0.40, 1.68) | 3 | 79.9 % | 0.007 |
| Folic acid supplements and risk of CNS tumors | 0.65 (0.31, 1.33) | 2 | 44.7 % | 0.179 | 0.80 (0.57, 1.14) | 4 | 79.0 % | 0.001 |  |  |  |  | 0.67 (0.41, 1.09) | 2 | 65.4 % | 0.089 |
| Hormones and risk of leukemia | 1.81 (0.77, 4.25) | 3 | 85.0 % | 0.001 | 1.43 (1.10, 1.84) | 4 | 0.0 % | 0.563 |  |  |  |  |  |  |  |  |
| Hormones and risk of ALL | 1.36 (0.96, 1.91) | 2 | 0.0 % | 0.319 | 0.97 (0.58, 1.61) | 2 | 73.0 % | 0.054 |  |  |  |  |  |  |  |  |
| Hormones and risk of neuroblastoma | 1.57 (1.02, 2.43) | 4 | 19.5 % | 0.292 | 1.80 (0.68, 4.77) | 1 |  |  |  |  |  |  |  |  |  |  |
| Oral contraceptives and risk of ALL | 1.29 (0.96, 1.72) | 3 | 8.6 % | 0.335 | 1.28 (0.86, 1.91) | 2 | 0.0 % | 0.896 |  |  |  |  |  |  |  |  |
| Nervous system medication and risk of leukemia | 0.42 (0.12, 1.43) | 1 |  |  | 1.60 (0.56, 4.56) | 2 | 74.6 % | 0.047 |  |  |  |  |  |  |  |  |
| Nervous system medication and risk of ALL | 2.61 (0.58, 11.80) | 2 | 48.4 % | 0.164 | 1.28 (1.03, 1.61) | 3 | 0.0 % | 0.482 |  |  |  |  |  |  |  |  |
| Nervous system medication and risk of CNS tumors | 1.07 (0.63, 1.80) | 4 | 0.0 % | 0.491 | 1.66 (0.87, 3.16) | 2 | 0.0 % | 0.506 |  |  |  |  | 0.70 (0.20, 2.47) | 1 |  |  |
| Vitamin and mineral supplements and risk of leukemia | 0.87 (0.64, 1.18) | 2 | 0.0 % | 0.709 | 0.60 (0.47, 0.76) | 1 |  |  |  |  |  |  |  |  |  |  |
| Vitamin and mineral supplements and risk of ALL | 0.72 (0.50, 1.03) | 4 | 66.1 % | 0.031 | 0.89 (0.66, 1.19) | 3 | 68.8 % | 0.040 |  |  |  |  | 0.84 (0.45, 1.59) | 3 | 76.7 % | 0.014 |
| Vitamin and mineral supplements and risk of AML | 1.08 (0.64, 1.81) | 2 | 0.0 % | 0.737 | 0.92 (0.62, 1.35) | 3 | 22.2 % | 0.277 |  |  |  |  |  |  |  |  |
| Vitamin and mineral supplements and risk of CNS tumors | 0.83 (0.51, 1.35) | 2 | 51.1 % | 0.153 | 0.73 (0.55, 0.95) | 7 | 74.2 % | 0.001 | 0.70 (0.54, 0.90) | 1 |  |  | 0.73 (0.60, 0.90) | 2 | 0.0 % | 0.537 |
| Vitamin and mineral supplements and risk of neuroblastoma | 0.55 (0.41, 0.74) | 2 | 0.0 % | 0.542 | 1.39 (1.02, 1.90) | 2 | 0.0 % | 0.360 |  |  |  |  |  |  |  |  |
| Vitamin C supplements and risk of CNS tumors | 0.82 (0.30, 2.26) | 2 | 82.0 % | 0.018 | 0.50 (0.29, 0.87) | 1 |  |  | 0.50 (0.29, 0.87) | 1 |  |  | 0.67 (0.41, 1.10) | 2 | 57.2 % | 0.126 |
| Vitamin E supplements and risk of solid tumors | 0.68 (0.50, 0.91) | 3 | 14.5 % | 0.311 | 0.50 (0.31, 0.82) | 1 |  |  | 0.50 (0.31, 0.82) | 1 |  |  | 0.68 (0.39, 1.18) | 2 | 70.2 % | 0.067 |
| Vitamin A supplements and risk of solid tumors | 0.56 (0.37, 0.85) | 2 | 30.1 % | 0.232 | 0.40 (0.20, 0.80) | 1 |  |  | 0.40 (0.20, 0.80) | 1 |  |  | 0.63 (0.29, 1.35) | 2 | 74.6 % | 0.047 |
| Vitamin C supplements in trimester 1 and risk of CNS tumors | 0.97 (0.72, 1.31) | 2 | 0.0 % | 0.760 | 0.61 (0.19, 2.01) | 2 | 75.0 % | 0.045 | 1.00 (0.71, 1.41) | 1 |  |  | 0.89 (0.70, 1.14) | 2 | 0.0 % | 0.366 |
| Vitamin C supplements in trimester 2/3 and risk of CNS tumors | 0.92 (0.63, 1.36) | 3 | 0.0 % | 0.324 | 0.55 (0.15, 1.96) | 2 | 45.4 % | 0.176 | 0.80 (0.50, 1.29) | 1 |  |  | 0.79 (0.60, 1.05) | 2 | 0.0 % | 0.967 |

**Supplementary Table 5 Stratification by study region**

Oceania: Australia and New Zealand; the study by Preston-Martin et al. (1998) was conducted in America, Europe and Oceania, and thus counted multiple times

Abbreviations: AM, America; EU, Europe; AS, Asia; OC, Oceania; CI, confidence interval; OR, odds ratio; ALL, acute lymphocytic leukemia; AML, acute myeloid leukemia; CNS, central nervous system
