# Supplementary material for: Prenatal exposure to medication and risk of childhood cancer – a systematic review and meta-analysis
Source: BMC Cancer. 2025 Nov 21;25:1841. doi: 10.1186/s12885-025-15316-0 (PMC12667062; doi:10.1186/s12885-025-15316-0)
Supplement: Supplementary file 1 — Supplementary Material 1: Supplementary Figure 1. Prenatal exposure to analgesics and the risk of childhood cancer. Abbreviations: ES, estimate; n.a., not available. Supplementary Figure 2. Prenatal exposure to antibiotics and the risk of childhood cancer. Abbreviations: ES, estimate; 1estimates were calculated with four-square table; * calculation of crude estimates. Supplementary Figure 3. Prenatal exposure to antiemetics and the risk of childhood cancer. Abbreviations: ES, estimate; n.a., not available; 1estimates were calculated with four-square table; * calculation of crude estimates. Supplementary Figure 4. Prenatal exposure to antihistamines and the risk of childhood cancer. Abbreviations: ES, estimate; n.a., not available; 1estimates were calculated with four-square table; * calculation of crude estimates. Supplementary Figure 5. Prenatal exposure to antihypertensives and the risk of childhood cancer. Abbreviations: ES, estimate; n.a., not available. Supplementary Figure 6. Prenatal exposure to antiretroviral HIV-drugs and the risk of childhood cancer. Abbreviations: ES, estimate; n.a., not available; HIV, human immunodeficiency virus; * calculation of crude estimates. Supplementary Figure 7. Prenatal exposure to cold or cough remedies and the risk of childhood cancer. Abbreviations: ES, estimate; n.a., not available; 1estimates were calculated with four-square table; * calculation of crude estimates. Supplementary Figure 8. Prenatal exposure to diuretics and the risk of childhood cancer. Abbreviations: ES, estimate; n.a., not available; 1estimates were calculated with four-square table; *calculation of crude estimates. Supplementary Figure 9. Prenatal exposure to folic acid supplements and the risk of childhood cancer. Abbreviations: ES, estimate; n.a., not available. Supplementary Figure 10. Prenatal exposure to hormones and the risk of childhood cancer. Abbreviations: ES, estimate; n.a., not available; 1estimates were calculated with four-square table; *c [file 12885_2025_15316_MOESM1_ESM.zip › Supplementary Table 7 Stratification by exposure method_revised.docx]

| **Model** | **Low (ES (95%CI))** | **n** | **I^2^** | **P value** | **High (ES (95%CI))** | **n** | **I^2^** | **P value** |
| --- | --- | --- | --- | --- | --- | --- | --- | --- |
| Analgesics and risk of ALL | 1.10 (0.88, 1.37) | 5 | 0.0 % | 0.485 | 1.29 (1.00, 1.67) | 2 | 0.0 % | 0.985 |
| Analgesics and risk of AML | 0.90 (0.63, 1.28) | 3 | 0.0 % | 0.672 | 0.75 (0.36, 1.56) | 1 |  |  |
| Analgesics and risk of CNS tumors | 1.07 (0.70, 1.63) | 3 | 1.9 % | 0.361 | 1.05 (0.74, 1.50) | 3 | 0.0 % | 0.384 |
| Analgesics and risk of neuroblastoma | 1.28 (0.90, 1.82) | 3 | 41.1 % | 0.183 | 1.99 (1.07, 3.70) | 1 |  |  |
| Analgesics and risk of lymphoma | 1.06 (0.49, 2.29) | 1 |  |  | 1.85 (0.22, 15.32) | 2 | 84.9 % | 0.010 |
| Antibiotics and risk of acute leukemia | 0.94 (0.73, 1.21) | 3 | 0.0 % | 0.823 | 1.15 (0.98, 1.34) | 2 | 0.0 % | 0.441 |
| Antibiotics and risk of ALL | 1.07 (0.90, 1.27) | 4 | 0.0 % | 0.671 | 1.17 (1.02, 1.33) | 8 | 39.3 % | 0.117 |
| Antibiotics and risk of AML | 1.96 (0.79, 4.89) | 2 | 81.8 % | 0.019 | 0.98 (0.60, 1.59) | 3 | 69.0 % | 0.040 |
| Antibiotics and risk of CNS tumors | 1.56 (1.01, 2.40) | 1 |  |  | 1.05 (0.89, 1.24) | 4 | 31.7 % | 0.186 |
| Antibiotics and risk of germ cell tumors | 1.50 (0.82, 2.76) | 1 |  |  | 1.22 (0.66, 2.23) | 2 | 30.4 % | 0.231 |
| Antibiotics and risk of lymphoma | 1.53 (0.76, 3.09) | 1 |  |  | 1.10 (0.80, 1.52) | 4 | 0.0 % | 0.407 |
| Antibiotics and risk of medulloblastoma | 2.07 (1.03, 4.17) | 1 |  |  | 1.45 (0.98, 2.15) | 3 | 0.0 % | 0.655 |
| Antibiotics and risk of neuroblastoma | 1.01 (0.72, 1.42) | 2 | 5.4 % | 0.304 | 1.66 (1.06, 2.60) | 3 | 42.0 % | 0.178 |
| Antibiotics and risk of renal tumors | 1.12 (0.55, 2.29) | 1 |  |  | 0.91 (0.68, 1.20) | 3 | 0.0 % | 0.984 |
| Penicillin and risk of acute leukemia | 0.88 (0.62, 1.24) | 1 |  |  | 1.05 (0.89, 1.23) | 2 | 0.0 % | 0.335 |
| Penicillin and risk of solid tumors | 0.90 (0.61, 1.32) | 1 |  |  | 1.38 (1.03, 1.85) | 2 | 0.0 % | 0.404 |
| Amoxicillin and risk of childhood cancer | 0.85 (0.64, 1.13) | 2 | 0.0 % | 0.751 | 1.12 (0.87, 1.44) | 1 |  |  |
| Beta-lactam antibiotics and risk of childhood cancer | 0.60 (0.27, 1.34) | 1 |  |  | 1.17 (0.83, 1.66) | 2 | 37.4 % | 0.206 |
| Antiemetics and risk of acute leukemia | 1.46 (1.04, 2.05) | 4 | 0.0 % | 0.644 | 1.65 (0.70, 3.91) | 1 |  |  |
| Antiemetics and risk of ALL | 1.27 (1.01, 1.59) | 4 | 0.0 % | 0.929 | 0.90 (0.55, 1.48) | 1 |  |  |
| Antiemetics and risk of CNS tumors | 1.51 (0.88, 2.60) | 2 | 51.6 % | 0.150 | 0.88 (0.63, 1.22) | 2 | 0.0 % | 0.841 |
| Antiemetics and risk of lymphoma | 1.23 (0.51, 2.98) | 1 |  |  | 1.22 (0.67, 2.20) | 2 | 0.0 % | 0.555 |
| Antiemetics and risk of neuroblastoma | 1.16 (0.76, 1.77) | 2 | 0.0 % | 0.811 | 1.45 (0.67, 3.13) | 1 |  |  |
| Antihistamines and risk of CNS tumors | 1.11 (0.65, 1.88) | 4 | 0.0 % | 0.535 | 0.90 (0.61, 1.32) | 1 |  |  |
| Antihypertensives and risk of ALL | 1.96 (1.10, 3.51) | 2 | 0.0 % | 0.944 | 1.42 (0.79, 2.55) | 1 |  |  |
| Antihypertensives and risk of solid tumors | 2.06 (0.98, 4.33) | 3 | 0.0 % | 0.570 | 1.70 (0.77, 3.77) | 2 | 49.6 % | 0.159 |
| Diuretics and risk of CNS tumors | 0.98 (0.41, 2.31) | 3 | 26.3 % | 0.257 | 1.40 (0.84, 2.34) | 2 | 24.6 % | 0.249 |
| Folic acid supplements and risk of ALL | 0.75 (0.45, 1.24) | 5 | 88.2 % | 0.000 | 1.30 (0.87, 1.95) | 1 |  |  |
| Folic acid supplements and risk of AML | 0.63 (0.18, 2.21) | 2 | 75.8 % | 0.042 | 0.59 (0.22, 1.59) | 1 |  |  |
| Folic acid supplements and risk of CNS tumors | 0.81 (0.62, 1.05) | 4 | 76.3 % | 0.002 | 0.88 (0.46, 1.70) | 2 | 66.3 % | 0.085 |
| Hormones and risk of leukemia | 1.55 (0.98, 2.45) | 5 | 73.3 % | 0.005 | 1.50 (1.01, 2.22) | 2 | 0.0 % | 0.487 |
| Hormones and risk of ALL | 1.34 (1.02, 1.76) | 3 | 0.0 % | 0.602 | 0.77 (0.59, 1.01) | 1 |  |  |
| Oral contraceptives and risk of ALL | 1.29 (1.02, 1.63) | 4 | 0.0 % | 0.534 | 1.22 (0.53, 2.81) | 1 |  |  |
| Nervous system medication and risk of leukemia | 1.16 (0.17, 7.66) | 2 | 84.0 % | 0.012 | 0.99 (0.56, 1.75) | 1 |  |  |
| Nervous system medication and risk of ALL | 2.61 (0.58, 11.80) | 2 | 48.4 % | 0.164 | 1.28 (1.03, 1.61) | 3 | 0.0 % | 0.482 |
| Nervous system medication and risk of CNS tumors | 1.04 (0.54, 2.02) | 4 | 0.0 % | 0.431 | 1.30 (0.80, 2.09) | 2 | 0.0 % | 0.429 |
| Vitamin and mineral supplements and risk of ALL | 0.79 (0.65, 0.95) | 9 | 58.6 % | 0.013 | 1.31 (0.89, 1.93) | 1 |  |  |
| Vitamin and mineral supplements and risk of AML | 0.98 (0.73, 1.31) | 4 | 0.0 % | 0.413 | 0.96 (0.43, 2.16) | 1 |  |  |
| Vitamin and mineral supplements and risk of CNS tumors | 0.70 (0.49, 1.01) | 6 | 76.3 % | 0.001 | 0.84 (0.66, 1.06) | 3 | 38.0 % | 0.199 |
| Vitamin and mineral supplements and risk of neuroblastoma | 0.77 (0.38, 1.55) | 3 | 89.5 % | 0.000 | 1.05 (0.53, 2.07) | 1 |  |  |

**Supplementary Table 7 Stratification by exposure method**

Low: self-reports; high: medical documentation, registry data

Abbreviations: CI, confidence interval; OR, odds ratio; ALL, acute lymphocytic leukemia; AML, acute myeloid leukemia; CNS, central nervous system
