# Supplementary material for: Prenatal exposure to medication and risk of childhood cancer – a systematic review and meta-analysis
Source: BMC Cancer. 2025 Nov 21;25:1841. doi: 10.1186/s12885-025-15316-0 (PMC12667062; doi:10.1186/s12885-025-15316-0)
Supplement: Supplementary file 1 — Supplementary Material 1: Supplementary Figure 1. Prenatal exposure to analgesics and the risk of childhood cancer. Abbreviations: ES, estimate; n.a., not available. Supplementary Figure 2. Prenatal exposure to antibiotics and the risk of childhood cancer. Abbreviations: ES, estimate; 1estimates were calculated with four-square table; * calculation of crude estimates. Supplementary Figure 3. Prenatal exposure to antiemetics and the risk of childhood cancer. Abbreviations: ES, estimate; n.a., not available; 1estimates were calculated with four-square table; * calculation of crude estimates. Supplementary Figure 4. Prenatal exposure to antihistamines and the risk of childhood cancer. Abbreviations: ES, estimate; n.a., not available; 1estimates were calculated with four-square table; * calculation of crude estimates. Supplementary Figure 5. Prenatal exposure to antihypertensives and the risk of childhood cancer. Abbreviations: ES, estimate; n.a., not available. Supplementary Figure 6. Prenatal exposure to antiretroviral HIV-drugs and the risk of childhood cancer. Abbreviations: ES, estimate; n.a., not available; HIV, human immunodeficiency virus; * calculation of crude estimates. Supplementary Figure 7. Prenatal exposure to cold or cough remedies and the risk of childhood cancer. Abbreviations: ES, estimate; n.a., not available; 1estimates were calculated with four-square table; * calculation of crude estimates. Supplementary Figure 8. Prenatal exposure to diuretics and the risk of childhood cancer. Abbreviations: ES, estimate; n.a., not available; 1estimates were calculated with four-square table; *calculation of crude estimates. Supplementary Figure 9. Prenatal exposure to folic acid supplements and the risk of childhood cancer. Abbreviations: ES, estimate; n.a., not available. Supplementary Figure 10. Prenatal exposure to hormones and the risk of childhood cancer. Abbreviations: ES, estimate; n.a., not available; 1estimates were calculated with four-square table; *c [file 12885_2025_15316_MOESM1_ESM.zip › Supplementary Table 8 Stratification by controlling for confounders_revised.docx]

| **Model** | **Low (ES (95%CI))** | **n** | **I^2^** | **P value** | **High (ES (95%CI))** | **n** | **I^2^** | **P value** |
| --- | --- | --- | --- | --- | --- | --- | --- | --- |
| Acetaminophen and risk of childhood cancer | 1.14 (0.84, 1.54) | 2 | 0.0 % | 0.455 | 0.81 (0.45, 1.45) | 2 | 56.5 % | 0.130 |
| Aspirin and risk of childhood cancer | 1.20 (0.56, 2.59) | 1 |  |  | 1.49 (0.95, 2.36) | 3 | 0.0 % | 0.820 |
| Analgesics and risk of ALL | 1.17 (0.93, 1.48) | 4 | 4.2 % | 0.372 | 1.18 (0.90, 1.54) | 3 | 0.0 % | 0.557 |
| Analgesics and risk of AML | 0.86 (0.49, 1.50) | 2 | 0.0 % | 0.577 | 0.87 (0.59, 1.28) | 2 | 0.0 % | 0.413 |
| Analgesics and risk of neuroblastoma | 1.26 (0.88, 1.78) | 3 | 33.3 % | 0.223 | 1.90 (1.13, 3.19) | 1 |  |  |
| Antibiotics and risk of acute leukemia | 0.88 (0.62, 1.24) | 1 |  |  | 1.13 (0.98, 1.30) | 4 | 0.0 % | 0.811 |
| Antibiotics and risk of ALL | 1.18 (1.01, 1.39) | 7 | 47.4 % | 0.077 | 1.10 (0.97, 1.26) | 5 | 0.0 % | 0.735 |
| Antibiotics and risk of AML | 1.64 (0.95, 2.84) | 3 | 72.0 % | 0.028 | 0.85 (0.39, 1.86) | 2 | 79.7 % | 0.026 |
| Antibiotics and risk of CNS tumors | 1.09 (0.88, 1.35) | 7 | 46.3 % | 0.083 | 1.08 (0.81, 1.37) | 1 |  |  |
| Antibiotics and risk of germ cell tumors | 1.50 (1.01, 2.24) | 2 | 0.0 % | 1.000 | 0.77 (0.30, 2.00) | 1 |  |  |
| Antibiotics and risk of lymphoma | 1.23 (0.90, 1.67) | 4 | 0.0 % | 0.442 | 0.78 (0.33, 1.86) | 1 |  |  |
| Antibiotics and risk of renal tumors | 0.97 (0.68, 1.40) | 3 | 0.0 % | 0.896 | 0.89 (0.61, 1.31) | 1 |  |  |
| Nitrosatable antibiotics and risk of childhood cancer | 1.30 (1.05, 1.61) | 1 |  |  | 1.35 (1.07, 1.70) | 2 | 0.0 % | 0.977 |
| Amoxicillin and risk of childhood cancer | 0.85 (0.64, 1.13) | 2 | 0.0 % | 0.751 | 1.12 (0.87, 1.44) | 1 |  |  |
| Beta-lactam antibiotics and risk of childhood cancer | 0.98 (0.47, 2.05) | 2 | 70.1 % | 0.068 | 0.88 (0.50, 1.56) | 1 |  |  |
| Antibiotics in trimester 1 and risk of ALL | 1.48 (0.57, 3.86) | 1 |  |  | 1.19 (0.89, 1.57) | 2 | 0.0 % | 0.644 |
| Antibiotics in trimester 2 and risk of ALL | 0.58 (0.21, 1.61) | 1 |  |  | 1.08 (0.82, 1.43) | 2 | 0.1 % | 0.317 |
| Antibiotics in trimester 3 and risk of ALL | 1.66 (0.88, 3.12) | 1 |  |  | 0.89 (0.65, 1.22) | 2 | 0.0 % | 0.599 |
| Antiemetics and risk of acute leukemia | 1.57 (0.92, 2.66) | 2 | 0.0 % | 0.708 | 1.44 (0.97, 2.14) | 3 | 0.0 % | 0.465 |
| Antiemetics and risk of ALL | 1.19 (0.88, 1.60) | 2 | 0.0 % | 0.934 | 1.20 (0.90, 1.60) | 3 | 0.0 % | 0.378 |
| Antiemetics and risk of CNS tumors | 1.25 (0.78, 1.98) | 3 | 62.0 % | 0.072 | 0.84 (0.50, 1.42) | 1 |  |  |
| Antiemetics and risk of lymphoma | 1.36 (0.63, 2.95) | 2 | 0.0 % | 0.644 | 1.13 (0.60, 2.14) | 1 |  |  |
| Antiemetics and risk of neuroblastoma | 1.07 (0.48, 2.38) | 1 |  |  | 1.27 (0.83, 1.93) | 2 | 0.0 % | 0.686 |
| Antihistamines and risk of acute leukemia | 1.70 (0.49, 5.84) | 1 |  |  | 1.46 (0.96, 2.23) | 2 | 0.0 % | 0.754 |
| Antihypertensives and risk of ALL | 1.99 (1.01, 3.93) | 1 |  |  | 1.51 (0.90, 2.54) | 2 | 0.0 % | 0.649 |
| Antihypertensives and risk of solid tumors | 2.87 (1.42, 5.80) | 2 | 0.0 % | 0.830 | 1.28 (0.72, 2.26) | 3 | 0.0 % | 0.885 |
| Diuretics and risk of CNS tumors | 1.17 (0.82, 1.69) | 4 | 0.0 % | 0.432 | 2.18 (0.87, 5.47) | 1 |  |  |
| Folic acid supplements in trimester 1 and risk of leukemia | 1.14 (0.95, 1.37) | 2 | 0.0 % | 0.678 | 0.30 (0.17, 0.52) | 1 |  |  |
| Folic acid supplements in trimester 2/3 and risk of leukemia | 0.95 (0.72, 1.26) | 2 | 59.0 % | 0.118 | 0.60 (0.33, 1.10) | 1 |  |  |
| Folic acid supplements and risk of leukemia | 1.12 (0.89, 1.41) | 2 | 0.0 % | 0.725 | 0.40 (0.28, 0.57) | 1 |  |  |
| Folic acid supplements and risk of ALL | 1.14 (0.95, 1.37) | 3 | 0.0 % | 0.914 | 0.60 (0.26, 1.37) | 3 | 90.5 % | 0.000 |
| Folic acid supplements and risk of AML | 1.10 (0.60, 2.01) | 1 |  |  | 0.44 (0.21, 0.91) | 2 | 0.0 % | 0.371 |
| Folic acid supplements and risk of CNS tumors | 0.67 (0.48, 0.93) | 3 | 36.2 % | 0.209 | 0.98 (0.83, 1.16) | 3 | 0.0 % | 0.603 |
| Hormones and risk of leukemia | 2.00 (0.89, 4.46) | 3 | 81.5 % | 0.005 | 1.33 (0.96, 1.83) | 4 | 36.4 % | 0.194 |
| Hormones and risk of neuroblastoma | 1.54 (1.03, 2.29) | 5 | 14.6 % | 0.322 | 1.20 (0.61, 2.35) | 1 |  |  |
| Oral contraceptives and risk of ALL | 1.44 (1.08, 1.91) | 3 | 0.0 % | 0.836 | 1.05 (0.72, 1.53) | 2 | 0.0 % | 0.692 |
| Nervous system medication and risk of leukemia | 0.99 (0.56, 1.75) | 1 |  |  | 1.16 (0.17, 7.66) | 2 | 84.0 % | 0.012 |
| Nervous system medication and risk of ALL | 1.82 (0.93, 3.55) | 3 | 56.4 % | 0.101 | 1.07 (0.51, 2.21) | 2 | 0.0 % | 0.864 |
| Nervous system medication and risk of CNS tumors | 1.15 (0.68, 1.94) | 5 | 0.0 % | 0.561 | 1.33 (0.62, 2.84) | 2 | 40.3 % | 0.195 |
| Vitamin and mineral supplements and risk of leukemia | 0.87 (0.64, 1.18) | 2 | 0.0 % | 0.709 | 0.60 (0.47, 0.76) | 1 |  |  |
| Vitamin and mineral supplements and risk of ALL | 0.89 (0.73, 1.09) | 6 | 49.5 % | 0.078 | 0.74 (0.51, 1.10) | 4 | 74.8 % | 0.008 |
| Vitamin and mineral supplements and risk of AML | 1.14 (0.79, 1.67) | 2 | 0.0 % | 0.899 | 0.81 (0.54, 1.22) | 3 | 0.0 % | 0.505 |
| Vitamin and mineral supplements and risk of CNS tumors | 0.80 (0.64, 1.01) | 6 | 70.9 % | 0.004 | 0.64 (0.30, 1.35) | 3 | 68.3 % | 0.043 |
| Vitamin and mineral supplements and risk of neuroblastoma | 0.77 (0.38, 1.55) | 3 | 89.5 % | 0.000 | 1.05 (0.53, 2.07) | 1 |  |  |
| Vitamin C supplements and risk of CNS tumors | 0.67 (0.41, 1.10) | 2 | 57.2 % | 0.126 | 1.40 (0.73, 2.70) | 1 |  |  |
| Vitamin E supplements and risk of solid tumors | 0.50 (0.31, 0.82) | 1 |  |  | 0.76 (0.57, 1.02) | 2 | 0.0 % | 0.644 |
| Vitamin A supplements and risk of solid tumors | 0.63 (0.29, 1.35) | 2 | 74.6 % | 0.047 | 0.64 (0.46, 0.90) | 1 |  |  |
| Vitamin and mineral supplements in trimester 1 and risk of CNS tumors | 0.89 (0.70, 1.14) | 2 | 0.0 % | 0.366 | 0.57 (0.19, 1.70) | 2 | 65.7 % | 0.088 |
| Vitamin and mineral supplements in trimester 2/3 and risk of CNS tumors | 0.79 (0.60, 1.05) | 2 | 0.0 % | 0.967 | 0.61 (0.10, 3.63) | 2 | 64.9 % | 0.092 |

**Supplementary Table 8 Stratification by controlling for confounders**

Low: basic or no adjustment; high: adjustment for other diseases, types of medication, pesticides, X-rays/ionizing radiation, alcohol or nicotine.

Abbreviations: CI, confidence interval; OR, odds ratio; ALL, acute lymphocytic leukemia; AML, acute myeloid leukemia; CNS, central nervous system
