# Supplementary material for: Prenatal exposure to medication and risk of childhood cancer – a systematic review and meta-analysis
Source: BMC Cancer. 2025 Nov 21;25:1841. doi: 10.1186/s12885-025-15316-0 (PMC12667062; doi:10.1186/s12885-025-15316-0)
Supplement: Supplementary file 1 — Supplementary Material 1: Supplementary Figure 1. Prenatal exposure to analgesics and the risk of childhood cancer. Abbreviations: ES, estimate; n.a., not available. Supplementary Figure 2. Prenatal exposure to antibiotics and the risk of childhood cancer. Abbreviations: ES, estimate; 1estimates were calculated with four-square table; * calculation of crude estimates. Supplementary Figure 3. Prenatal exposure to antiemetics and the risk of childhood cancer. Abbreviations: ES, estimate; n.a., not available; 1estimates were calculated with four-square table; * calculation of crude estimates. Supplementary Figure 4. Prenatal exposure to antihistamines and the risk of childhood cancer. Abbreviations: ES, estimate; n.a., not available; 1estimates were calculated with four-square table; * calculation of crude estimates. Supplementary Figure 5. Prenatal exposure to antihypertensives and the risk of childhood cancer. Abbreviations: ES, estimate; n.a., not available. Supplementary Figure 6. Prenatal exposure to antiretroviral HIV-drugs and the risk of childhood cancer. Abbreviations: ES, estimate; n.a., not available; HIV, human immunodeficiency virus; * calculation of crude estimates. Supplementary Figure 7. Prenatal exposure to cold or cough remedies and the risk of childhood cancer. Abbreviations: ES, estimate; n.a., not available; 1estimates were calculated with four-square table; * calculation of crude estimates. Supplementary Figure 8. Prenatal exposure to diuretics and the risk of childhood cancer. Abbreviations: ES, estimate; n.a., not available; 1estimates were calculated with four-square table; *calculation of crude estimates. Supplementary Figure 9. Prenatal exposure to folic acid supplements and the risk of childhood cancer. Abbreviations: ES, estimate; n.a., not available. Supplementary Figure 10. Prenatal exposure to hormones and the risk of childhood cancer. Abbreviations: ES, estimate; n.a., not available; 1estimates were calculated with four-square table; *c [file 12885_2025_15316_MOESM1_ESM.zip › Supplementary Table 9 Stratification by study quality_revised.docx]

| **Model** | **Low (ES (95%CI))** | **n** | **I^2^** | **P value** | **High (ES (95%CI))** | **n** | **I^2^** | **P value** |
| --- | --- | --- | --- | --- | --- | --- | --- | --- |
| Antibiotics and risk of leukemia | 0.94 (0.73, 1.21) | 3 | 0.0 % | 0.823 | 1.15 (0.98, 1.34) | 2 | 0.0 % | 0.441 |
| Antibiotics and risk of ALL | 1.10 (0.96, 1.27) | 8 | 34.4 % | 0.153 | 1.22 (1.07, 1.39) | 4 | 0.0 % | 0.830 |
| Antibiotics and risk of AML | 1.51 (1.03, 2.23) | 4 | 60.2 % | 0.056 | 0.57 (0.34, 0.96) | 1 |  |  |
| Antibiotics and risk of CNS tumors | 1.06 (0.79, 1.41) | 5 | 64.0 % | 0.025 | 1.10 (0.90, 1.34) | 3 | 0.0 % | 0.833 |
| Antibiotics and risk of germ cell tumors | 1.50 (1.01, 2.24) | 2 | 0.0 % | 1.000 | 0.77 (0.30, 2.00) | 1 |  |  |
| Antibiotics and risk of lymphoma | 1.23 (0.90, 1.67) | 4 | 0.0 % | 0.442 | 0.78 (0.33, 1.85) | 1 |  |  |
| Antibiotics and risk of medulloblastoma | 1.51 (1.04, 2.20) | 3 | 0.0 % | 0.524 | 1.96 (0.85, 4.51) | 1 |  |  |
| Antibiotics and risk of neuroblastoma | 1.28 (0.88, 1.85) | 4 | 59.4 % | 0.061 | 2.60 (0.89, 7.59) | 1 |  |  |
| Antibiotics and risk of renal tumors | 0.97 (0.68, 1.40) | 3 | 0.0 % | 0.896 | 0.89 (0.61, 1.31) | 1 |  |  |
| Nitrosatable antibiotics and risk of childhood cancer | 1.30 (1.05, 1.61) | 1 |  |  | 1.35 (1.07, 1.70) | 2 | 0.0 % | 0.977 |
| Amoxicillin and risk of childhood cancer | 0.85 (0.64, 1.13) | 2 | 0.0 % | 0.751 | 1.12 (0.87, 1.44) | 1 |  |  |
| Penicillin and risk of leukemia | 0.95 (0.79, 1.14) | 2 | 0.0 % | 0.601 | 1.15 (0.90, 1.47) | 1 |  |  |
| Penicillin and risk of solid tumors | 1.20 (0.63, 2.26) | 2 | 68.8 % | 0.074 | 1.29 (0.92, 1.80) | 1 |  |  |
| Beta-lactam antibiotics and risk of childhood cancer | 0.60 (0.27, 1.34) | 1 |  |  | 1.17 (0.83, 1.66) | 2 | 37.4 % | 0.206 |
| Antibiotics in trimester 1 and risk of ALL | 1.48 (0.57, 3.86) | 1 |  |  | 1.19 (0.89, 1.57) | 2 | 0.0 % | 0.644 |
| Antibiotics in trimester 2 and risk of ALL | 0.58 (0.21, 1.61) | 1 |  |  | 1.08 (0.82, 1.43) | 2 | 0.1 % | 0.317 |
| Antibiotics in trimester 3 and risk of ALL | 1.66 (0.88, 3.12) | 1 |  |  | 0.89 (0.65, 1.22) | 2 | 0.0 % | 0.599 |
| Antiemetics and risk of leukemia | 1.46 (1.04, 2.05) | 4 | 0.0 % | 0.644 | 1.65 (0.70, 3.91) | 1 |  |  |
| Antiemetics and risk of ALL | 1.27 (1.01, 1.59) | 4 | 0.0 % | 0.929 | 0.90 (0.55, 1.48) | 1 |  |  |
| Antiemetics and risk of CNS tumors | 1.25 (0.78, 1.98) | 3 | 62.0 % | 0.072 | 0.84 (0.50, 1.42) | 1 |  |  |
| Antiemetics and risk of lymphoma | 1.36 (0.63, 2.95) | 2 | 0.0 % | 0.644 | 1.13 (0.60, 2.14) | 1 |  |  |
| Antiemetics and risk of neuroblastoma | 1.16 (0.76, 1.77) | 2 | 0.0 % | 0.811 | 1.45 (0.67, 3.13) | 1 |  |  |
| Folic acid supplements and risk of ALL | 0.75 (0.45, 1.24) | 5 | 88.2 % | 0.000 | 1.30 (0.87, 1.95) | 1 |  |  |
| Folic acid supplements and risk of AML | 0.63 (0.18, 2.21) | 2 | 75.8 % | 0.042 | 0.59 (0.22, 1.59) | 1 |  |  |
| Folic acid supplements and risk of CNS tumors | 0.78 (0.61, 1.00) | 5 | 42.3 % | 0.139 | 1.18 (0.78, 1.78) | 1 |  |  |
| Hormones and risk of leukemia | 1.47 (1.02, 2.13) | 6 | 66.7 % | 0.010 | 1.78 (0.95, 3.32) | 1 |  |  |
| Hormones and risk of ALL | 1.09 (076, 1.57) | 4 | 65.9 % | 0.032 | 1.57 (1.10, 2.25) | 1 |  |  |
| Oral contraceptives and risk of ALL | 1.29 (1.02, 1.63) | 4 | 0.0 % | 0.534 | 1.22 (0.53, 2.81) | 1 |  |  |
| Vitamin and mineral supplements and risk of ALL | 0.79 (0.65, 0.95) | 9 | 58.6 % | 0.013 | 1.31 (0.89, 1.93) | 1 |  |  |
| Vitamin and mineral supplements and risk of AML | 0.98 (0.73, 1.31) | 4 | 0.0 % | 0.413 | 0.96 (0.43, 2.16) | 1 |  |  |
| Vitamin and mineral supplements and risk of CNS tumors | 0.78 (0.61, 0.99) | 8 | 70.3 % | 0.001 | 0.68 (0.42, 1.10) | 1 |  |  |
| Vitamin and mineral supplements and risk of neuroblastoma | 0.77 (0.38, 1.55) | 3 | 89.5 % | 0.000 | 1.05 (0.53, 2.07) | 1 |  |  |

**Supplementary Table 9 Stratification by study quality**

Low: quality score <28.5, high: quality score ≥28.5

Abbreviations: CI, confidence interval; OR, odds ratio; ALL, acute lymphocytic leukemia; AML, acute myeloid leukemia; CNS, central nervous system
