# Supplementary figures and images for: Prenatal exposure to medication and risk of childhood cancer – a systematic review and meta-analysis
Source: BMC Cancer. 2025 Nov 21;25:1841. doi: 10.1186/s12885-025-15316-0 (PMC12667062; doi:10.1186/s12885-025-15316-0)

**
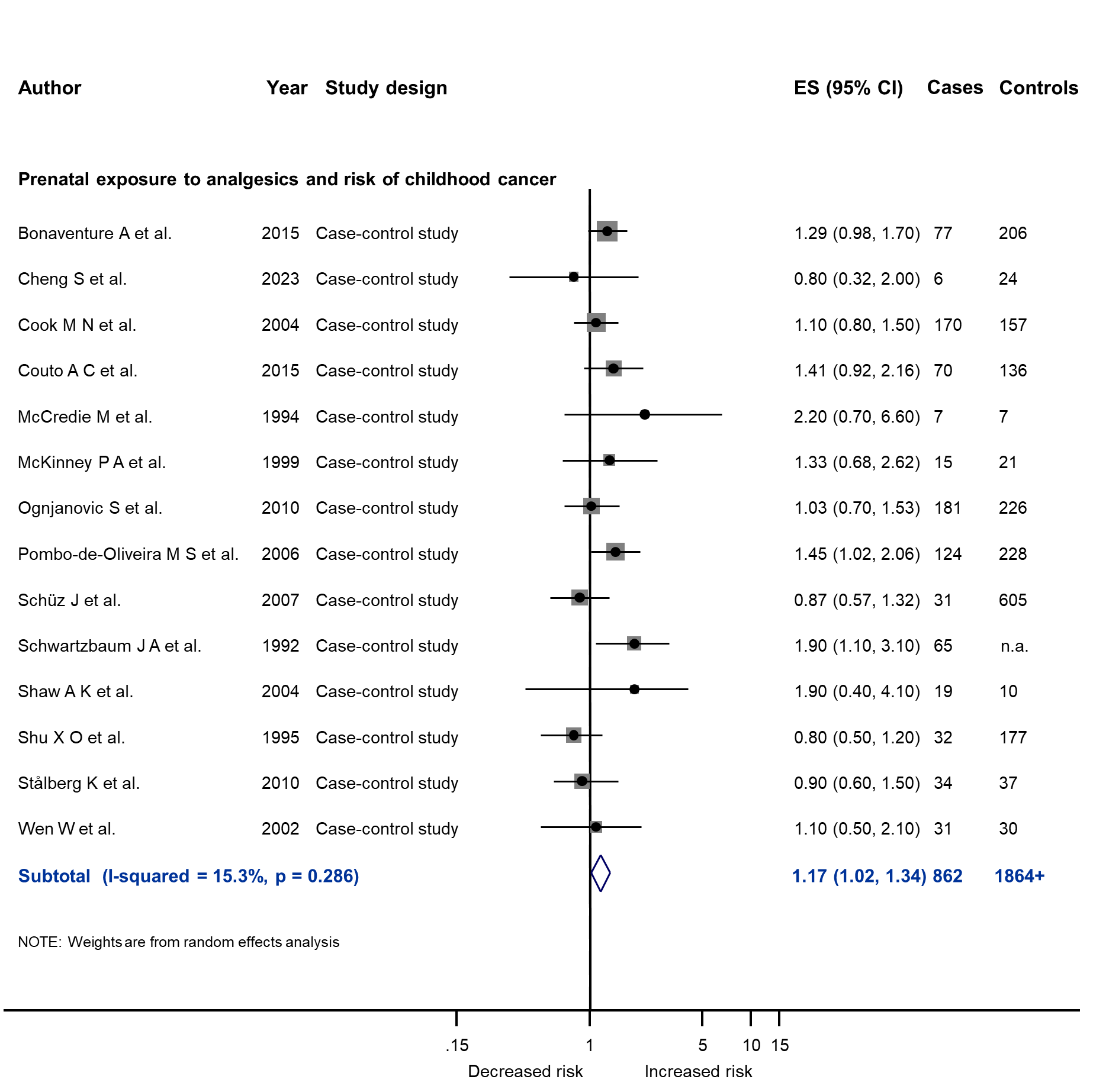
**

Supplement: Supplementary file 1 — Supplementary Material 1: Supplementary Figure 1. Prenatal exposure to analgesics and the risk of childhood cancer. Abbreviations: ES, estimate; n.a., not available. Supplementary Figure 2. Prenatal exposure to antibiotics and the risk of childhood cancer. Abbreviations: ES, estimate; 1estimates were calculated with four-square table; * calculation of crude estimates. Supplementary Figure 3. Prenatal exposure to antiemetics and the risk of childhood cancer. Abbreviations: ES, estimate; n.a., not available; 1estimates were calculated with four-square table; * calculation of crude estimates. Supplementary Figure 4. Prenatal exposure to antihistamines and the risk of childhood cancer. Abbreviations: ES, estimate; n.a., not available; 1estimates were calculated with four-square table; * calculation of crude estimates. Supplementary Figure 5. Prenatal exposure to antihypertensives and the risk of childhood cancer. Abbreviations: ES, estimate; n.a., not available. Supplementary Figure 6. Prenatal exposure to antiretroviral HIV-drugs and the risk of childhood cancer. Abbreviations: ES, estimate; n.a., not available; HIV, human immunodeficiency virus; * calculation of crude estimates. Supplementary Figure 7. Prenatal exposure to cold or cough remedies and the risk of childhood cancer. Abbreviations: ES, estimate; n.a., not available; 1estimates were calculated with four-square table; * calculation of crude estimates. Supplementary Figure 8. Prenatal exposure to diuretics and the risk of childhood cancer. Abbreviations: ES, estimate; n.a., not available; 1estimates were calculated with four-square table; *calculation of crude estimates. Supplementary Figure 9. Prenatal exposure to folic acid supplements and the risk of childhood cancer. Abbreviations: ES, estimate; n.a., not available. Supplementary Figure 10. Prenatal exposure to hormones and the risk of childhood cancer. Abbreviations: ES, estimate; n.a., not available; 1estimates were calculated with four-square table; *c [file 12885_2025_15316_MOESM1_ESM.zip › Supplementary Figure 1 Prenatal exposure to analgesics and the risk of childhood cancer.docx]

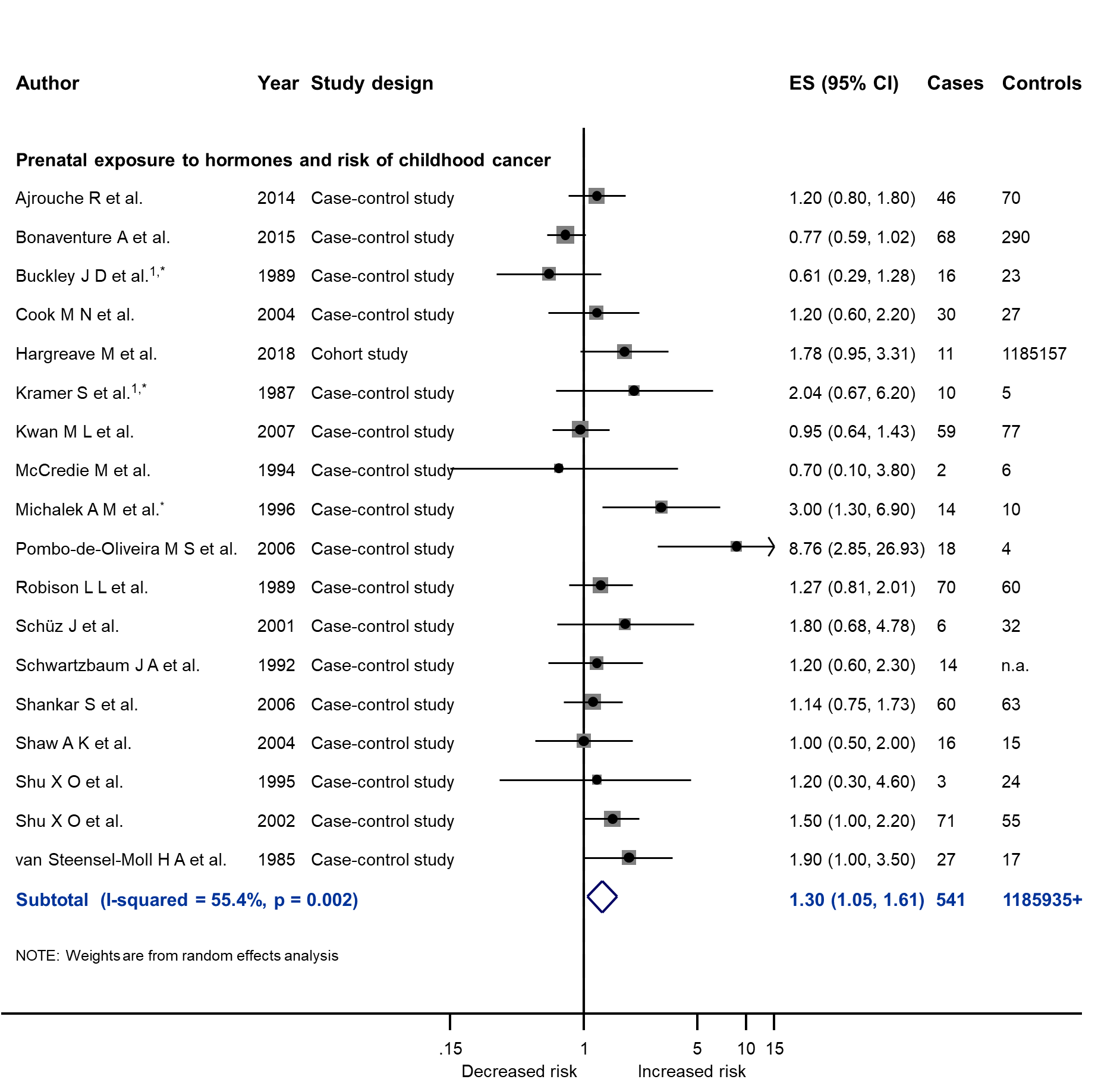

Supplement: Supplementary file 1 — Supplementary Material 1: Supplementary Figure 1. Prenatal exposure to analgesics and the risk of childhood cancer. Abbreviations: ES, estimate; n.a., not available. Supplementary Figure 2. Prenatal exposure to antibiotics and the risk of childhood cancer. Abbreviations: ES, estimate; 1estimates were calculated with four-square table; * calculation of crude estimates. Supplementary Figure 3. Prenatal exposure to antiemetics and the risk of childhood cancer. Abbreviations: ES, estimate; n.a., not available; 1estimates were calculated with four-square table; * calculation of crude estimates. Supplementary Figure 4. Prenatal exposure to antihistamines and the risk of childhood cancer. Abbreviations: ES, estimate; n.a., not available; 1estimates were calculated with four-square table; * calculation of crude estimates. Supplementary Figure 5. Prenatal exposure to antihypertensives and the risk of childhood cancer. Abbreviations: ES, estimate; n.a., not available. Supplementary Figure 6. Prenatal exposure to antiretroviral HIV-drugs and the risk of childhood cancer. Abbreviations: ES, estimate; n.a., not available; HIV, human immunodeficiency virus; * calculation of crude estimates. Supplementary Figure 7. Prenatal exposure to cold or cough remedies and the risk of childhood cancer. Abbreviations: ES, estimate; n.a., not available; 1estimates were calculated with four-square table; * calculation of crude estimates. Supplementary Figure 8. Prenatal exposure to diuretics and the risk of childhood cancer. Abbreviations: ES, estimate; n.a., not available; 1estimates were calculated with four-square table; *calculation of crude estimates. Supplementary Figure 9. Prenatal exposure to folic acid supplements and the risk of childhood cancer. Abbreviations: ES, estimate; n.a., not available. Supplementary Figure 10. Prenatal exposure to hormones and the risk of childhood cancer. Abbreviations: ES, estimate; n.a., not available; 1estimates were calculated with four-square table; *c [file 12885_2025_15316_MOESM1_ESM.zip › Supplementary Figure 10 Prenatal exposure to hormones and the risk of childhood cancer_revised.docx]

**
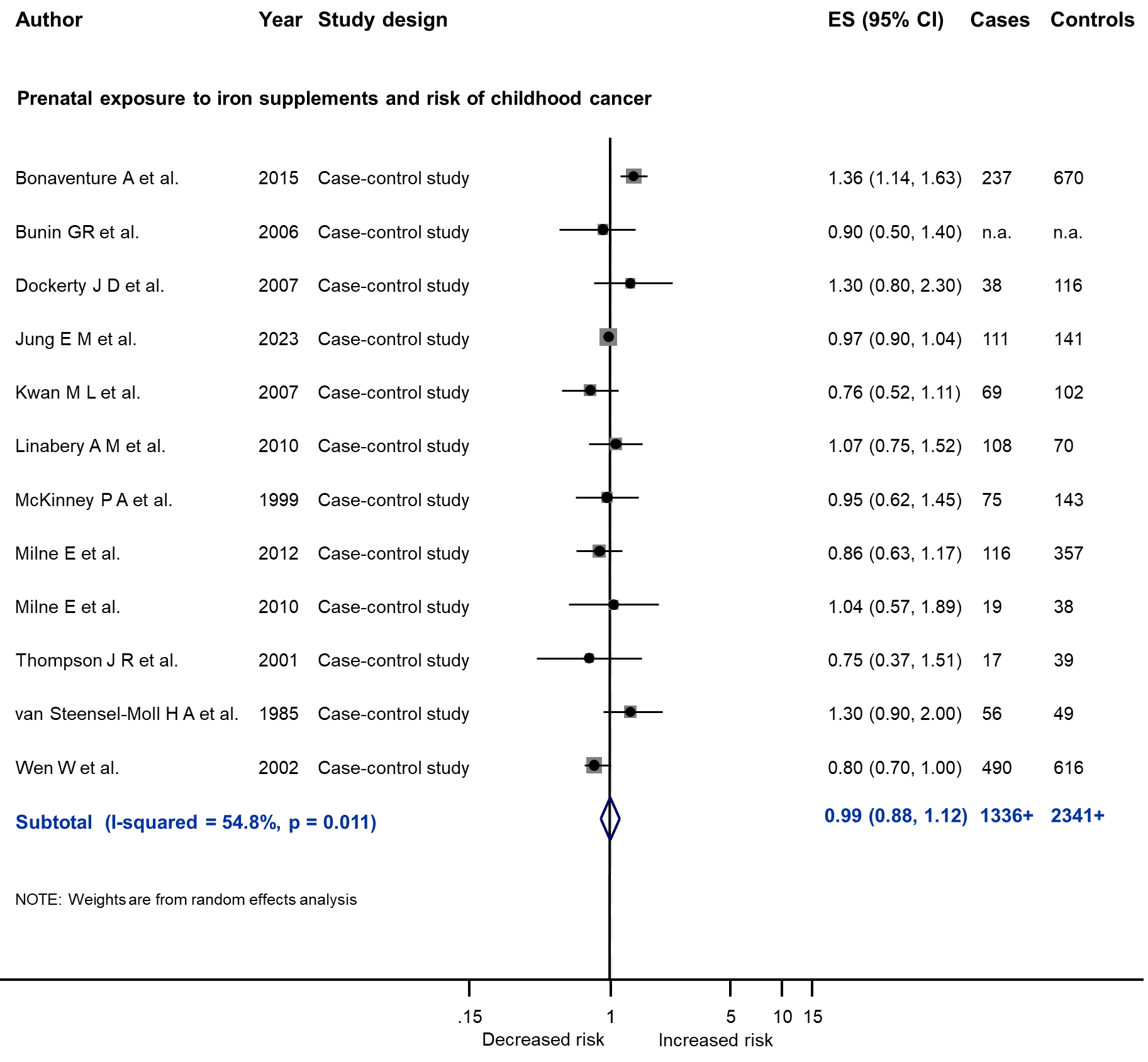
**

Supplement: Supplementary file 1 — Supplementary Material 1: Supplementary Figure 1. Prenatal exposure to analgesics and the risk of childhood cancer. Abbreviations: ES, estimate; n.a., not available. Supplementary Figure 2. Prenatal exposure to antibiotics and the risk of childhood cancer. Abbreviations: ES, estimate; 1estimates were calculated with four-square table; * calculation of crude estimates. Supplementary Figure 3. Prenatal exposure to antiemetics and the risk of childhood cancer. Abbreviations: ES, estimate; n.a., not available; 1estimates were calculated with four-square table; * calculation of crude estimates. Supplementary Figure 4. Prenatal exposure to antihistamines and the risk of childhood cancer. Abbreviations: ES, estimate; n.a., not available; 1estimates were calculated with four-square table; * calculation of crude estimates. Supplementary Figure 5. Prenatal exposure to antihypertensives and the risk of childhood cancer. Abbreviations: ES, estimate; n.a., not available. Supplementary Figure 6. Prenatal exposure to antiretroviral HIV-drugs and the risk of childhood cancer. Abbreviations: ES, estimate; n.a., not available; HIV, human immunodeficiency virus; * calculation of crude estimates. Supplementary Figure 7. Prenatal exposure to cold or cough remedies and the risk of childhood cancer. Abbreviations: ES, estimate; n.a., not available; 1estimates were calculated with four-square table; * calculation of crude estimates. Supplementary Figure 8. Prenatal exposure to diuretics and the risk of childhood cancer. Abbreviations: ES, estimate; n.a., not available; 1estimates were calculated with four-square table; *calculation of crude estimates. Supplementary Figure 9. Prenatal exposure to folic acid supplements and the risk of childhood cancer. Abbreviations: ES, estimate; n.a., not available. Supplementary Figure 10. Prenatal exposure to hormones and the risk of childhood cancer. Abbreviations: ES, estimate; n.a., not available; 1estimates were calculated with four-square table; *c [file 12885_2025_15316_MOESM1_ESM.zip › Supplementary Figure 11 Prenatal exposure to iron supplements and the risk of childhood cancer.docx]

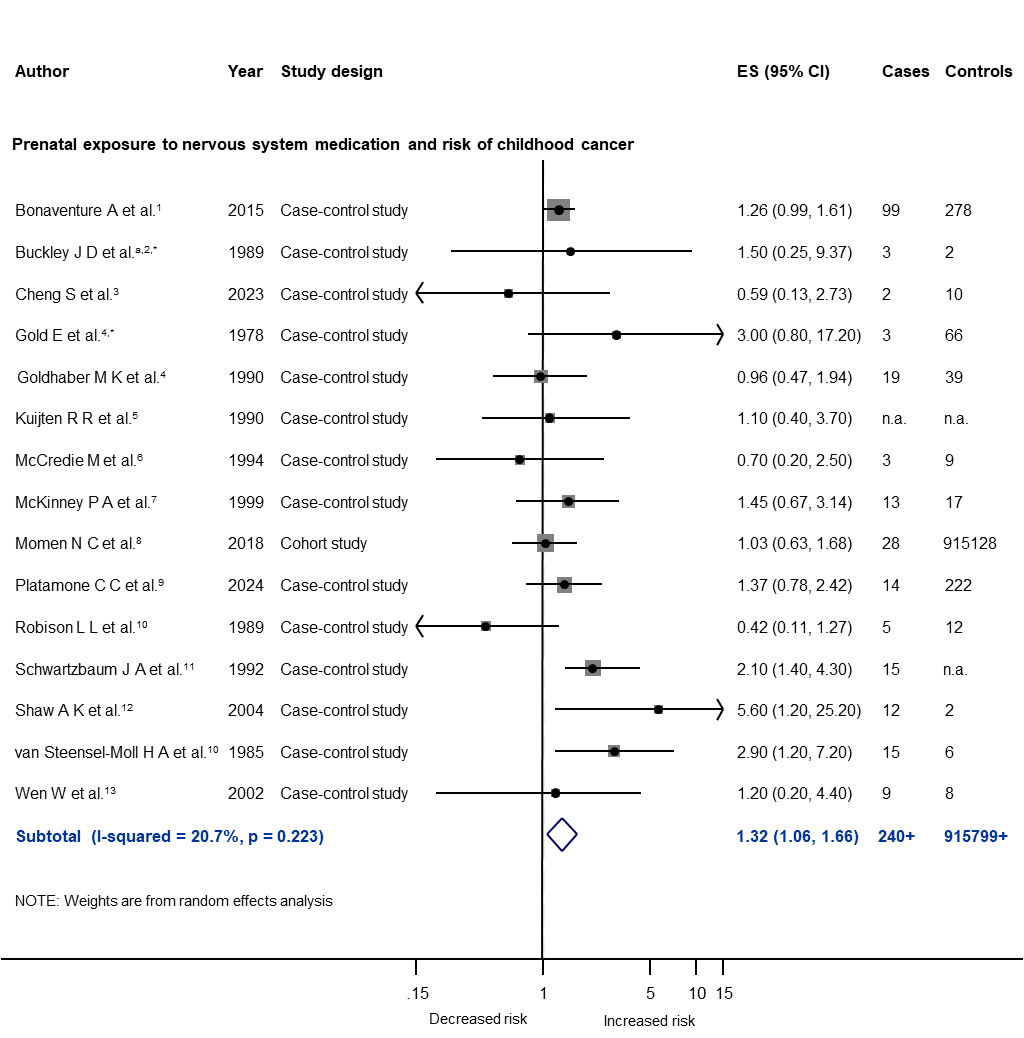

Supplement: Supplementary file 1 — Supplementary Material 1: Supplementary Figure 1. Prenatal exposure to analgesics and the risk of childhood cancer. Abbreviations: ES, estimate; n.a., not available. Supplementary Figure 2. Prenatal exposure to antibiotics and the risk of childhood cancer. Abbreviations: ES, estimate; 1estimates were calculated with four-square table; * calculation of crude estimates. Supplementary Figure 3. Prenatal exposure to antiemetics and the risk of childhood cancer. Abbreviations: ES, estimate; n.a., not available; 1estimates were calculated with four-square table; * calculation of crude estimates. Supplementary Figure 4. Prenatal exposure to antihistamines and the risk of childhood cancer. Abbreviations: ES, estimate; n.a., not available; 1estimates were calculated with four-square table; * calculation of crude estimates. Supplementary Figure 5. Prenatal exposure to antihypertensives and the risk of childhood cancer. Abbreviations: ES, estimate; n.a., not available. Supplementary Figure 6. Prenatal exposure to antiretroviral HIV-drugs and the risk of childhood cancer. Abbreviations: ES, estimate; n.a., not available; HIV, human immunodeficiency virus; * calculation of crude estimates. Supplementary Figure 7. Prenatal exposure to cold or cough remedies and the risk of childhood cancer. Abbreviations: ES, estimate; n.a., not available; 1estimates were calculated with four-square table; * calculation of crude estimates. Supplementary Figure 8. Prenatal exposure to diuretics and the risk of childhood cancer. Abbreviations: ES, estimate; n.a., not available; 1estimates were calculated with four-square table; *calculation of crude estimates. Supplementary Figure 9. Prenatal exposure to folic acid supplements and the risk of childhood cancer. Abbreviations: ES, estimate; n.a., not available. Supplementary Figure 10. Prenatal exposure to hormones and the risk of childhood cancer. Abbreviations: ES, estimate; n.a., not available; 1estimates were calculated with four-square table; *c [file 12885_2025_15316_MOESM1_ESM.zip › Supplementary Figure 12 Prenatal exposure to nervous system medication and the risk of childhood cancer_revised.docx]

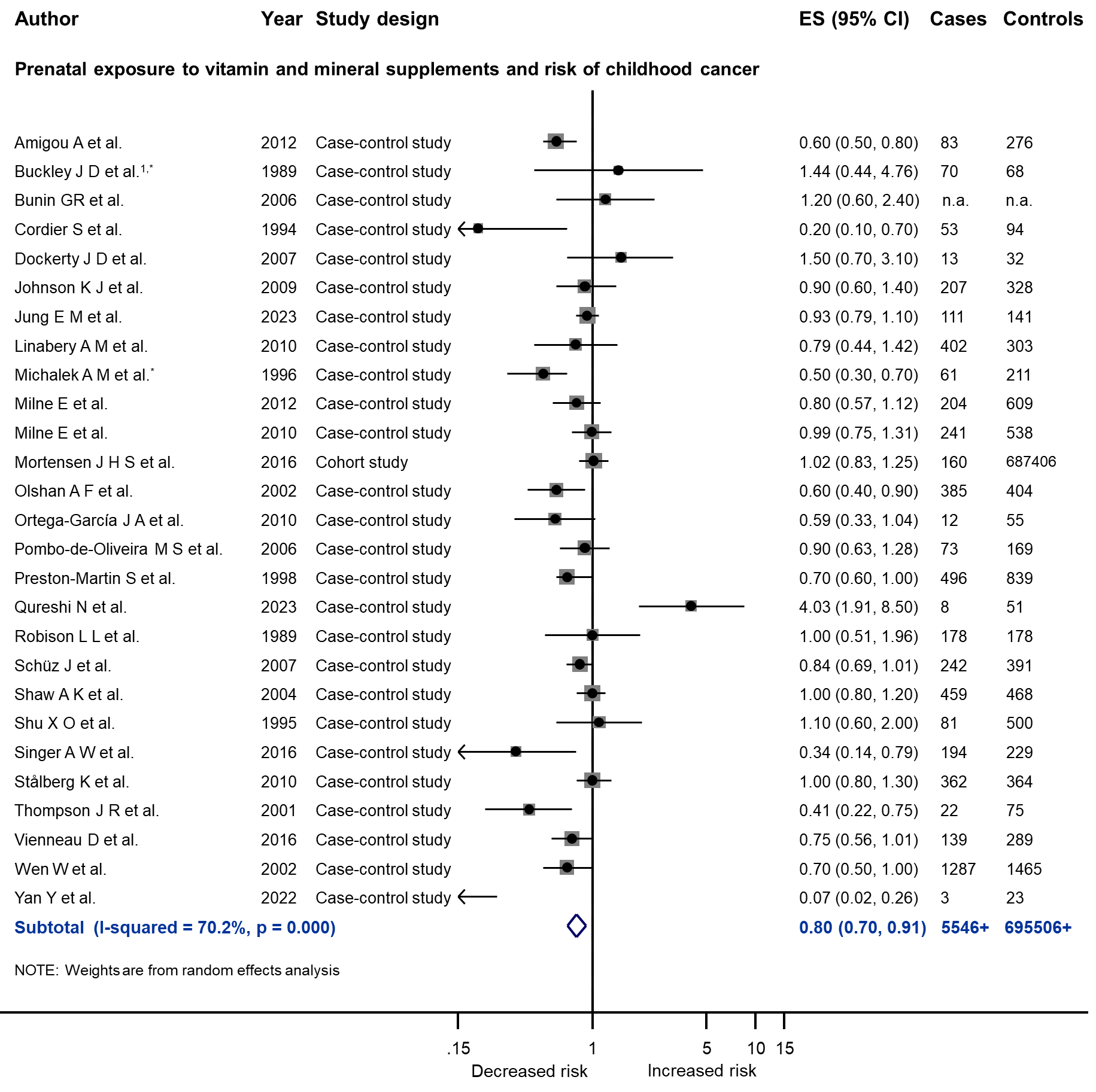

Supplement: Supplementary file 1 — Supplementary Material 1: Supplementary Figure 1. Prenatal exposure to analgesics and the risk of childhood cancer. Abbreviations: ES, estimate; n.a., not available. Supplementary Figure 2. Prenatal exposure to antibiotics and the risk of childhood cancer. Abbreviations: ES, estimate; 1estimates were calculated with four-square table; * calculation of crude estimates. Supplementary Figure 3. Prenatal exposure to antiemetics and the risk of childhood cancer. Abbreviations: ES, estimate; n.a., not available; 1estimates were calculated with four-square table; * calculation of crude estimates. Supplementary Figure 4. Prenatal exposure to antihistamines and the risk of childhood cancer. Abbreviations: ES, estimate; n.a., not available; 1estimates were calculated with four-square table; * calculation of crude estimates. Supplementary Figure 5. Prenatal exposure to antihypertensives and the risk of childhood cancer. Abbreviations: ES, estimate; n.a., not available. Supplementary Figure 6. Prenatal exposure to antiretroviral HIV-drugs and the risk of childhood cancer. Abbreviations: ES, estimate; n.a., not available; HIV, human immunodeficiency virus; * calculation of crude estimates. Supplementary Figure 7. Prenatal exposure to cold or cough remedies and the risk of childhood cancer. Abbreviations: ES, estimate; n.a., not available; 1estimates were calculated with four-square table; * calculation of crude estimates. Supplementary Figure 8. Prenatal exposure to diuretics and the risk of childhood cancer. Abbreviations: ES, estimate; n.a., not available; 1estimates were calculated with four-square table; *calculation of crude estimates. Supplementary Figure 9. Prenatal exposure to folic acid supplements and the risk of childhood cancer. Abbreviations: ES, estimate; n.a., not available. Supplementary Figure 10. Prenatal exposure to hormones and the risk of childhood cancer. Abbreviations: ES, estimate; n.a., not available; 1estimates were calculated with four-square table; *c [file 12885_2025_15316_MOESM1_ESM.zip › Supplementary Figure 13 Prenatal exposure to vitamin and mineral supplements and the risk of childhood cancer_revised.docx]

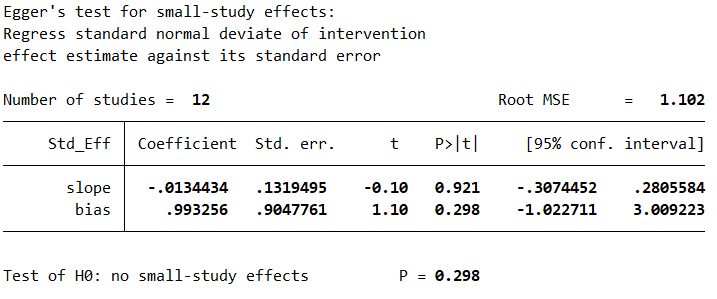

Supplement: Supplementary file 1 — Supplementary Material 1: Supplementary Figure 1. Prenatal exposure to analgesics and the risk of childhood cancer. Abbreviations: ES, estimate; n.a., not available. Supplementary Figure 2. Prenatal exposure to antibiotics and the risk of childhood cancer. Abbreviations: ES, estimate; 1estimates were calculated with four-square table; * calculation of crude estimates. Supplementary Figure 3. Prenatal exposure to antiemetics and the risk of childhood cancer. Abbreviations: ES, estimate; n.a., not available; 1estimates were calculated with four-square table; * calculation of crude estimates. Supplementary Figure 4. Prenatal exposure to antihistamines and the risk of childhood cancer. Abbreviations: ES, estimate; n.a., not available; 1estimates were calculated with four-square table; * calculation of crude estimates. Supplementary Figure 5. Prenatal exposure to antihypertensives and the risk of childhood cancer. Abbreviations: ES, estimate; n.a., not available. Supplementary Figure 6. Prenatal exposure to antiretroviral HIV-drugs and the risk of childhood cancer. Abbreviations: ES, estimate; n.a., not available; HIV, human immunodeficiency virus; * calculation of crude estimates. Supplementary Figure 7. Prenatal exposure to cold or cough remedies and the risk of childhood cancer. Abbreviations: ES, estimate; n.a., not available; 1estimates were calculated with four-square table; * calculation of crude estimates. Supplementary Figure 8. Prenatal exposure to diuretics and the risk of childhood cancer. Abbreviations: ES, estimate; n.a., not available; 1estimates were calculated with four-square table; *calculation of crude estimates. Supplementary Figure 9. Prenatal exposure to folic acid supplements and the risk of childhood cancer. Abbreviations: ES, estimate; n.a., not available. Supplementary Figure 10. Prenatal exposure to hormones and the risk of childhood cancer. Abbreviations: ES, estimate; n.a., not available; 1estimates were calculated with four-square table; *c [file 12885_2025_15316_MOESM1_ESM.zip › Supplementary Figure 14 Publication bias analysis on antibiotics and the risk of ALL.docx]

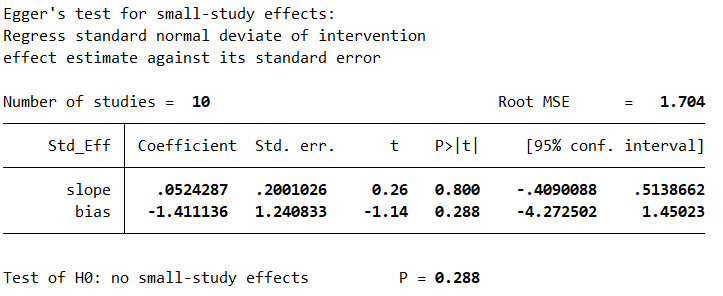

Supplement: Supplementary file 1 — Supplementary Material 1: Supplementary Figure 1. Prenatal exposure to analgesics and the risk of childhood cancer. Abbreviations: ES, estimate; n.a., not available. Supplementary Figure 2. Prenatal exposure to antibiotics and the risk of childhood cancer. Abbreviations: ES, estimate; 1estimates were calculated with four-square table; * calculation of crude estimates. Supplementary Figure 3. Prenatal exposure to antiemetics and the risk of childhood cancer. Abbreviations: ES, estimate; n.a., not available; 1estimates were calculated with four-square table; * calculation of crude estimates. Supplementary Figure 4. Prenatal exposure to antihistamines and the risk of childhood cancer. Abbreviations: ES, estimate; n.a., not available; 1estimates were calculated with four-square table; * calculation of crude estimates. Supplementary Figure 5. Prenatal exposure to antihypertensives and the risk of childhood cancer. Abbreviations: ES, estimate; n.a., not available. Supplementary Figure 6. Prenatal exposure to antiretroviral HIV-drugs and the risk of childhood cancer. Abbreviations: ES, estimate; n.a., not available; HIV, human immunodeficiency virus; * calculation of crude estimates. Supplementary Figure 7. Prenatal exposure to cold or cough remedies and the risk of childhood cancer. Abbreviations: ES, estimate; n.a., not available; 1estimates were calculated with four-square table; * calculation of crude estimates. Supplementary Figure 8. Prenatal exposure to diuretics and the risk of childhood cancer. Abbreviations: ES, estimate; n.a., not available; 1estimates were calculated with four-square table; *calculation of crude estimates. Supplementary Figure 9. Prenatal exposure to folic acid supplements and the risk of childhood cancer. Abbreviations: ES, estimate; n.a., not available. Supplementary Figure 10. Prenatal exposure to hormones and the risk of childhood cancer. Abbreviations: ES, estimate; n.a., not available; 1estimates were calculated with four-square table; *c [file 12885_2025_15316_MOESM1_ESM.zip › Supplementary Figure 15 Publication bias analysis on vitamin and mineral supplements and the risk of ALL.docx]

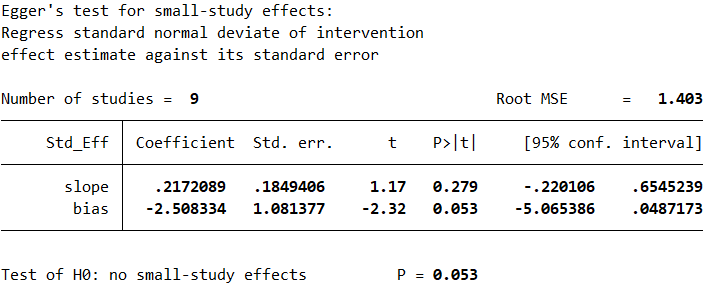

Supplement: Supplementary file 1 — Supplementary Material 1: Supplementary Figure 1. Prenatal exposure to analgesics and the risk of childhood cancer. Abbreviations: ES, estimate; n.a., not available. Supplementary Figure 2. Prenatal exposure to antibiotics and the risk of childhood cancer. Abbreviations: ES, estimate; 1estimates were calculated with four-square table; * calculation of crude estimates. Supplementary Figure 3. Prenatal exposure to antiemetics and the risk of childhood cancer. Abbreviations: ES, estimate; n.a., not available; 1estimates were calculated with four-square table; * calculation of crude estimates. Supplementary Figure 4. Prenatal exposure to antihistamines and the risk of childhood cancer. Abbreviations: ES, estimate; n.a., not available; 1estimates were calculated with four-square table; * calculation of crude estimates. Supplementary Figure 5. Prenatal exposure to antihypertensives and the risk of childhood cancer. Abbreviations: ES, estimate; n.a., not available. Supplementary Figure 6. Prenatal exposure to antiretroviral HIV-drugs and the risk of childhood cancer. Abbreviations: ES, estimate; n.a., not available; HIV, human immunodeficiency virus; * calculation of crude estimates. Supplementary Figure 7. Prenatal exposure to cold or cough remedies and the risk of childhood cancer. Abbreviations: ES, estimate; n.a., not available; 1estimates were calculated with four-square table; * calculation of crude estimates. Supplementary Figure 8. Prenatal exposure to diuretics and the risk of childhood cancer. Abbreviations: ES, estimate; n.a., not available; 1estimates were calculated with four-square table; *calculation of crude estimates. Supplementary Figure 9. Prenatal exposure to folic acid supplements and the risk of childhood cancer. Abbreviations: ES, estimate; n.a., not available. Supplementary Figure 10. Prenatal exposure to hormones and the risk of childhood cancer. Abbreviations: ES, estimate; n.a., not available; 1estimates were calculated with four-square table; *c [file 12885_2025_15316_MOESM1_ESM.zip › Supplementary Figure 16 Publication bias analysis on vitamin and mineral supplements and the risk of CNS tumors.docx]

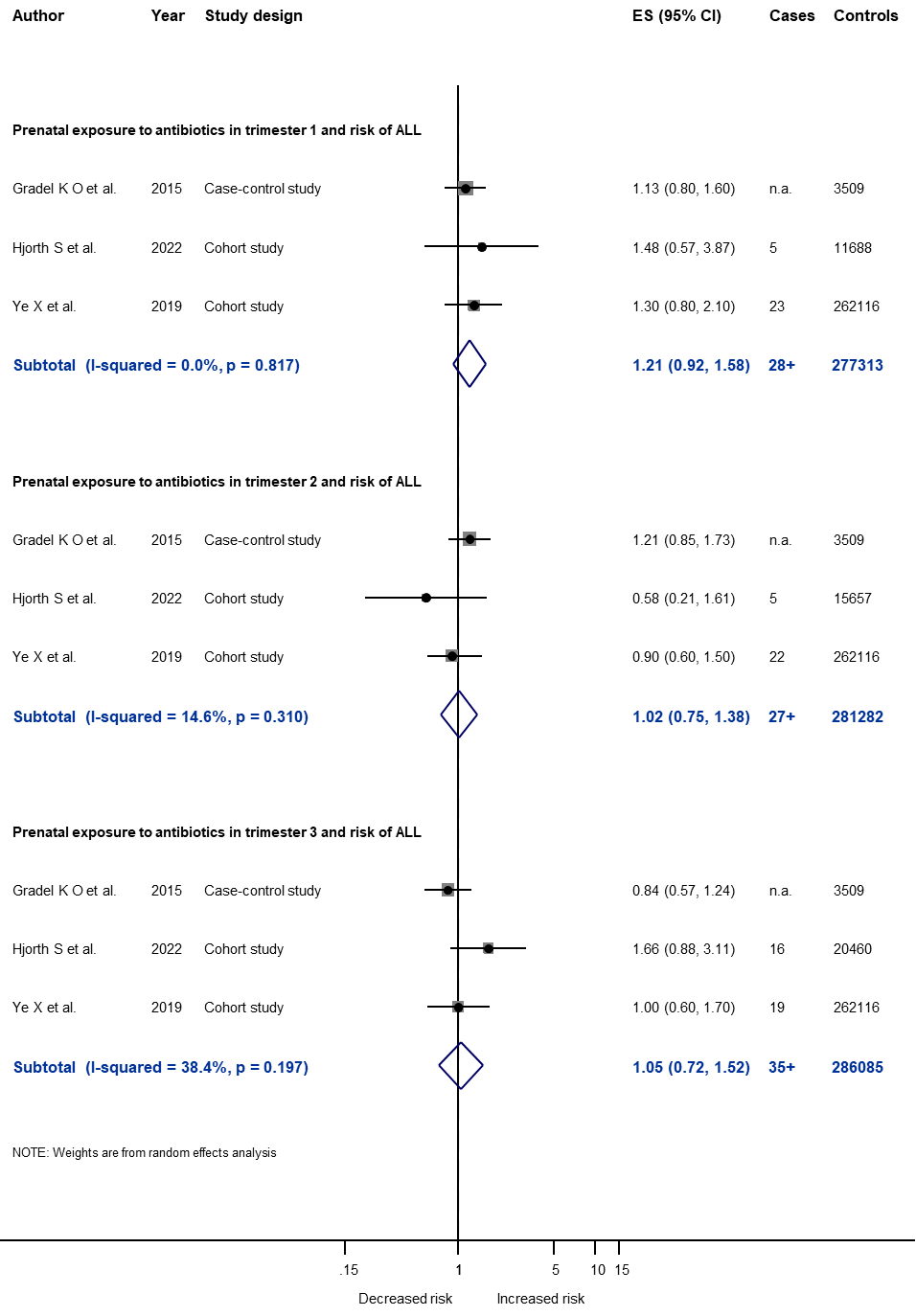

Supplement: Supplementary file 1 — Supplementary Material 1: Supplementary Figure 1. Prenatal exposure to analgesics and the risk of childhood cancer. Abbreviations: ES, estimate; n.a., not available. Supplementary Figure 2. Prenatal exposure to antibiotics and the risk of childhood cancer. Abbreviations: ES, estimate; 1estimates were calculated with four-square table; * calculation of crude estimates. Supplementary Figure 3. Prenatal exposure to antiemetics and the risk of childhood cancer. Abbreviations: ES, estimate; n.a., not available; 1estimates were calculated with four-square table; * calculation of crude estimates. Supplementary Figure 4. Prenatal exposure to antihistamines and the risk of childhood cancer. Abbreviations: ES, estimate; n.a., not available; 1estimates were calculated with four-square table; * calculation of crude estimates. Supplementary Figure 5. Prenatal exposure to antihypertensives and the risk of childhood cancer. Abbreviations: ES, estimate; n.a., not available. Supplementary Figure 6. Prenatal exposure to antiretroviral HIV-drugs and the risk of childhood cancer. Abbreviations: ES, estimate; n.a., not available; HIV, human immunodeficiency virus; * calculation of crude estimates. Supplementary Figure 7. Prenatal exposure to cold or cough remedies and the risk of childhood cancer. Abbreviations: ES, estimate; n.a., not available; 1estimates were calculated with four-square table; * calculation of crude estimates. Supplementary Figure 8. Prenatal exposure to diuretics and the risk of childhood cancer. Abbreviations: ES, estimate; n.a., not available; 1estimates were calculated with four-square table; *calculation of crude estimates. Supplementary Figure 9. Prenatal exposure to folic acid supplements and the risk of childhood cancer. Abbreviations: ES, estimate; n.a., not available. Supplementary Figure 10. Prenatal exposure to hormones and the risk of childhood cancer. Abbreviations: ES, estimate; n.a., not available; 1estimates were calculated with four-square table; *c [file 12885_2025_15316_MOESM1_ESM.zip › Supplementary Figure 17 Prenatal exposure to antibiotics in different trimesters and the risk of ALL in children_revised.docx]

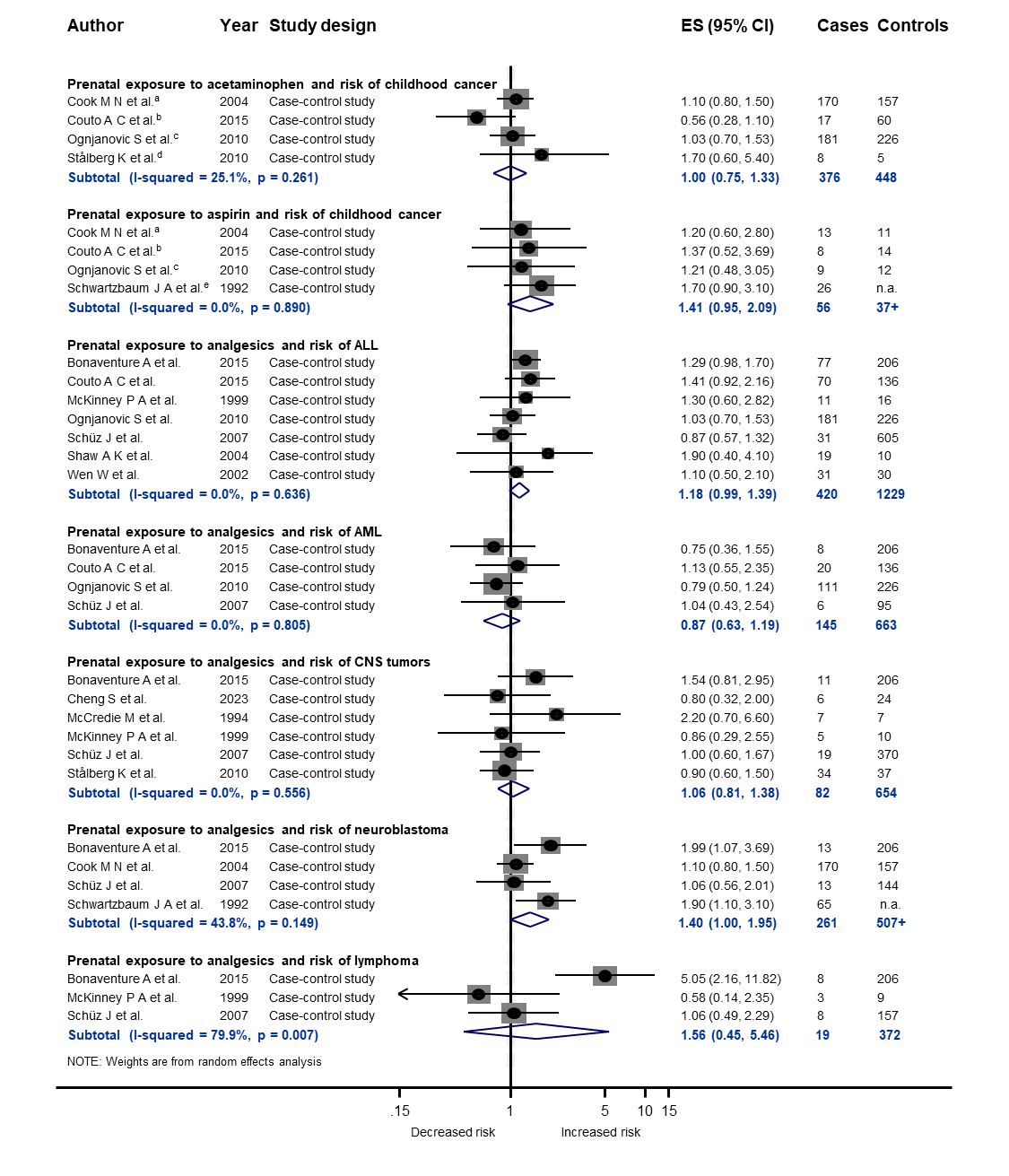

Supplement: Supplementary file 1 — Supplementary Material 1: Supplementary Figure 1. Prenatal exposure to analgesics and the risk of childhood cancer. Abbreviations: ES, estimate; n.a., not available. Supplementary Figure 2. Prenatal exposure to antibiotics and the risk of childhood cancer. Abbreviations: ES, estimate; 1estimates were calculated with four-square table; * calculation of crude estimates. Supplementary Figure 3. Prenatal exposure to antiemetics and the risk of childhood cancer. Abbreviations: ES, estimate; n.a., not available; 1estimates were calculated with four-square table; * calculation of crude estimates. Supplementary Figure 4. Prenatal exposure to antihistamines and the risk of childhood cancer. Abbreviations: ES, estimate; n.a., not available; 1estimates were calculated with four-square table; * calculation of crude estimates. Supplementary Figure 5. Prenatal exposure to antihypertensives and the risk of childhood cancer. Abbreviations: ES, estimate; n.a., not available. Supplementary Figure 6. Prenatal exposure to antiretroviral HIV-drugs and the risk of childhood cancer. Abbreviations: ES, estimate; n.a., not available; HIV, human immunodeficiency virus; * calculation of crude estimates. Supplementary Figure 7. Prenatal exposure to cold or cough remedies and the risk of childhood cancer. Abbreviations: ES, estimate; n.a., not available; 1estimates were calculated with four-square table; * calculation of crude estimates. Supplementary Figure 8. Prenatal exposure to diuretics and the risk of childhood cancer. Abbreviations: ES, estimate; n.a., not available; 1estimates were calculated with four-square table; *calculation of crude estimates. Supplementary Figure 9. Prenatal exposure to folic acid supplements and the risk of childhood cancer. Abbreviations: ES, estimate; n.a., not available. Supplementary Figure 10. Prenatal exposure to hormones and the risk of childhood cancer. Abbreviations: ES, estimate; n.a., not available; 1estimates were calculated with four-square table; *c [file 12885_2025_15316_MOESM1_ESM.zip › Supplementary Figure 18 Prenatal exposure to analgesics and the risk of different childhood cancer sites_revised.docx]

**
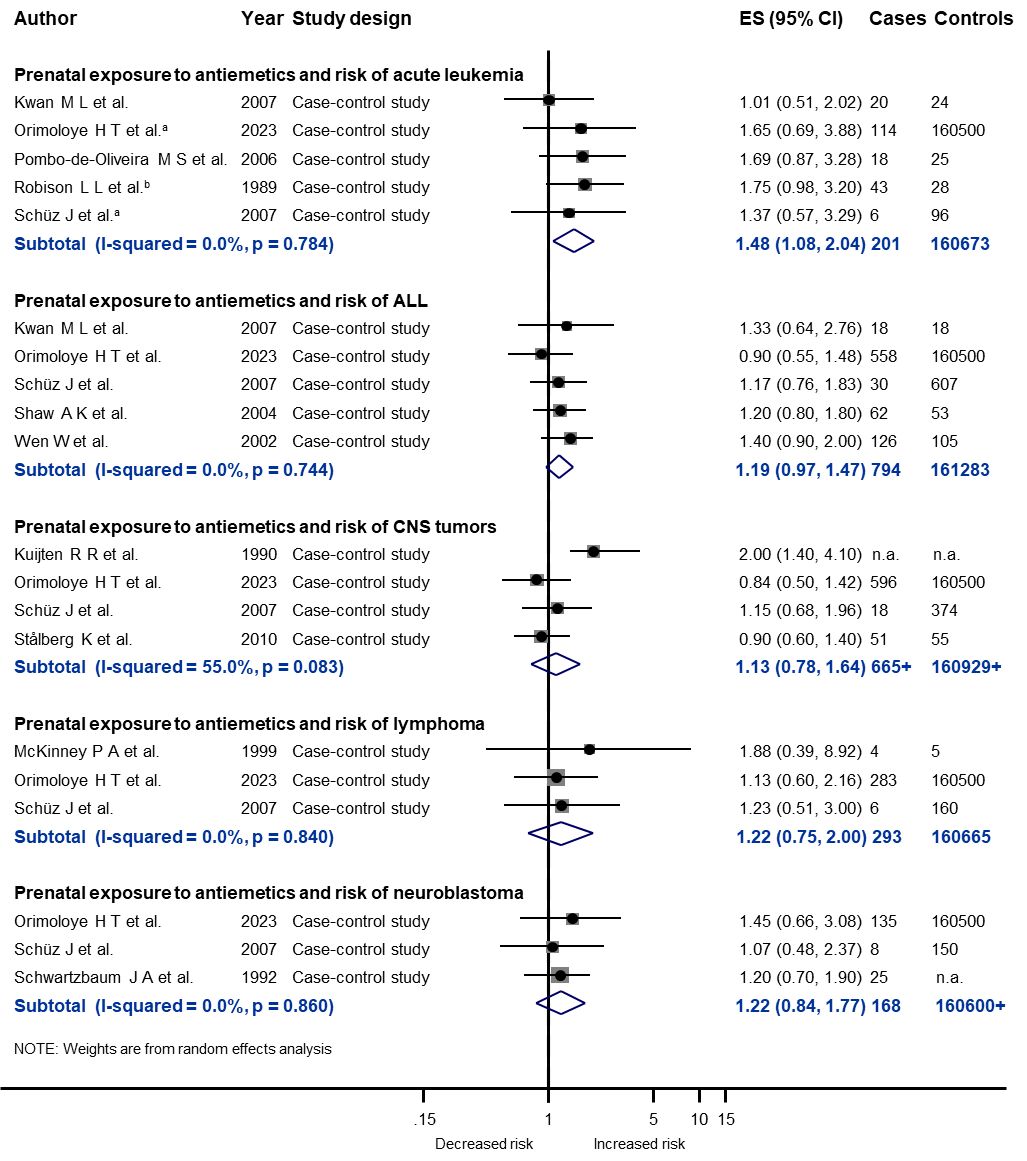
**

Supplement: Supplementary file 1 — Supplementary Material 1: Supplementary Figure 1. Prenatal exposure to analgesics and the risk of childhood cancer. Abbreviations: ES, estimate; n.a., not available. Supplementary Figure 2. Prenatal exposure to antibiotics and the risk of childhood cancer. Abbreviations: ES, estimate; 1estimates were calculated with four-square table; * calculation of crude estimates. Supplementary Figure 3. Prenatal exposure to antiemetics and the risk of childhood cancer. Abbreviations: ES, estimate; n.a., not available; 1estimates were calculated with four-square table; * calculation of crude estimates. Supplementary Figure 4. Prenatal exposure to antihistamines and the risk of childhood cancer. Abbreviations: ES, estimate; n.a., not available; 1estimates were calculated with four-square table; * calculation of crude estimates. Supplementary Figure 5. Prenatal exposure to antihypertensives and the risk of childhood cancer. Abbreviations: ES, estimate; n.a., not available. Supplementary Figure 6. Prenatal exposure to antiretroviral HIV-drugs and the risk of childhood cancer. Abbreviations: ES, estimate; n.a., not available; HIV, human immunodeficiency virus; * calculation of crude estimates. Supplementary Figure 7. Prenatal exposure to cold or cough remedies and the risk of childhood cancer. Abbreviations: ES, estimate; n.a., not available; 1estimates were calculated with four-square table; * calculation of crude estimates. Supplementary Figure 8. Prenatal exposure to diuretics and the risk of childhood cancer. Abbreviations: ES, estimate; n.a., not available; 1estimates were calculated with four-square table; *calculation of crude estimates. Supplementary Figure 9. Prenatal exposure to folic acid supplements and the risk of childhood cancer. Abbreviations: ES, estimate; n.a., not available. Supplementary Figure 10. Prenatal exposure to hormones and the risk of childhood cancer. Abbreviations: ES, estimate; n.a., not available; 1estimates were calculated with four-square table; *c [file 12885_2025_15316_MOESM1_ESM.zip › Supplementary Figure 19 Prenatal exposure to antiemetics and the risk of different childhood cancer sites_revised.docx]

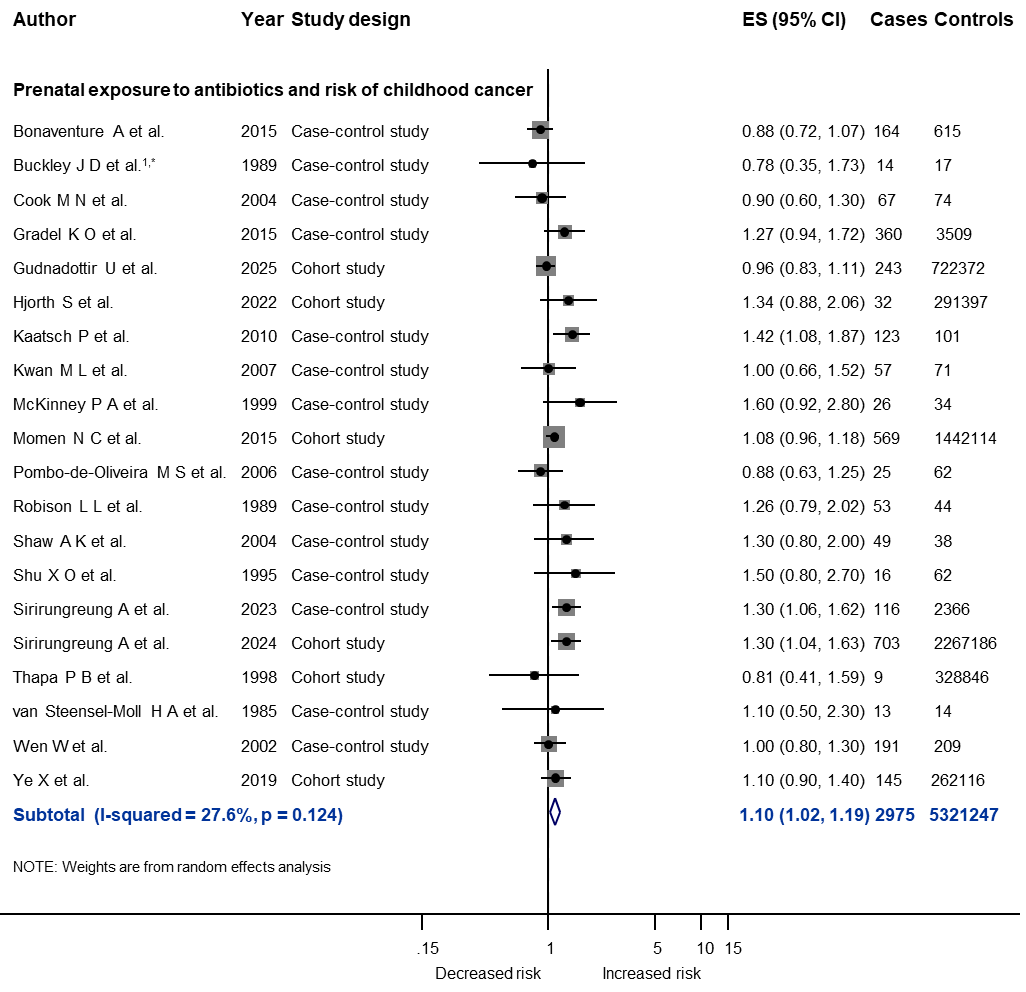

Supplement: Supplementary file 1 — Supplementary Material 1: Supplementary Figure 1. Prenatal exposure to analgesics and the risk of childhood cancer. Abbreviations: ES, estimate; n.a., not available. Supplementary Figure 2. Prenatal exposure to antibiotics and the risk of childhood cancer. Abbreviations: ES, estimate; 1estimates were calculated with four-square table; * calculation of crude estimates. Supplementary Figure 3. Prenatal exposure to antiemetics and the risk of childhood cancer. Abbreviations: ES, estimate; n.a., not available; 1estimates were calculated with four-square table; * calculation of crude estimates. Supplementary Figure 4. Prenatal exposure to antihistamines and the risk of childhood cancer. Abbreviations: ES, estimate; n.a., not available; 1estimates were calculated with four-square table; * calculation of crude estimates. Supplementary Figure 5. Prenatal exposure to antihypertensives and the risk of childhood cancer. Abbreviations: ES, estimate; n.a., not available. Supplementary Figure 6. Prenatal exposure to antiretroviral HIV-drugs and the risk of childhood cancer. Abbreviations: ES, estimate; n.a., not available; HIV, human immunodeficiency virus; * calculation of crude estimates. Supplementary Figure 7. Prenatal exposure to cold or cough remedies and the risk of childhood cancer. Abbreviations: ES, estimate; n.a., not available; 1estimates were calculated with four-square table; * calculation of crude estimates. Supplementary Figure 8. Prenatal exposure to diuretics and the risk of childhood cancer. Abbreviations: ES, estimate; n.a., not available; 1estimates were calculated with four-square table; *calculation of crude estimates. Supplementary Figure 9. Prenatal exposure to folic acid supplements and the risk of childhood cancer. Abbreviations: ES, estimate; n.a., not available. Supplementary Figure 10. Prenatal exposure to hormones and the risk of childhood cancer. Abbreviations: ES, estimate; n.a., not available; 1estimates were calculated with four-square table; *c [file 12885_2025_15316_MOESM1_ESM.zip › Supplementary Figure 2 Prenatal exposure to antibiotics and the risk of childhood cancer_revised.docx]

**
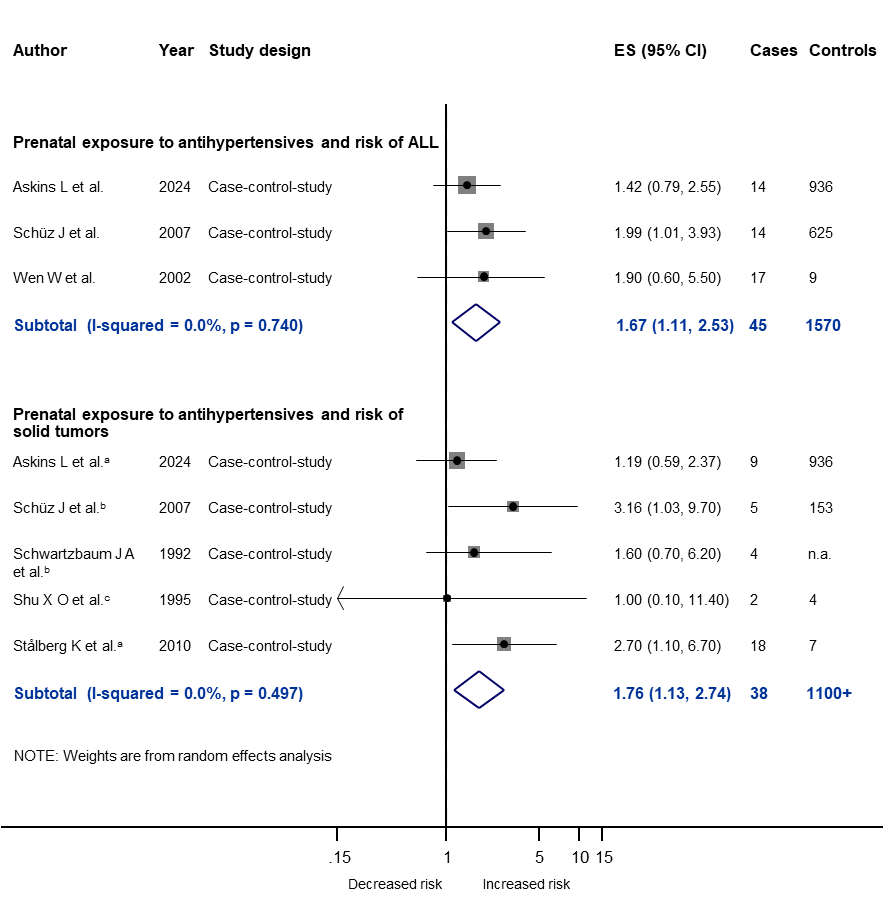
**

Supplement: Supplementary file 1 — Supplementary Material 1: Supplementary Figure 1. Prenatal exposure to analgesics and the risk of childhood cancer. Abbreviations: ES, estimate; n.a., not available. Supplementary Figure 2. Prenatal exposure to antibiotics and the risk of childhood cancer. Abbreviations: ES, estimate; 1estimates were calculated with four-square table; * calculation of crude estimates. Supplementary Figure 3. Prenatal exposure to antiemetics and the risk of childhood cancer. Abbreviations: ES, estimate; n.a., not available; 1estimates were calculated with four-square table; * calculation of crude estimates. Supplementary Figure 4. Prenatal exposure to antihistamines and the risk of childhood cancer. Abbreviations: ES, estimate; n.a., not available; 1estimates were calculated with four-square table; * calculation of crude estimates. Supplementary Figure 5. Prenatal exposure to antihypertensives and the risk of childhood cancer. Abbreviations: ES, estimate; n.a., not available. Supplementary Figure 6. Prenatal exposure to antiretroviral HIV-drugs and the risk of childhood cancer. Abbreviations: ES, estimate; n.a., not available; HIV, human immunodeficiency virus; * calculation of crude estimates. Supplementary Figure 7. Prenatal exposure to cold or cough remedies and the risk of childhood cancer. Abbreviations: ES, estimate; n.a., not available; 1estimates were calculated with four-square table; * calculation of crude estimates. Supplementary Figure 8. Prenatal exposure to diuretics and the risk of childhood cancer. Abbreviations: ES, estimate; n.a., not available; 1estimates were calculated with four-square table; *calculation of crude estimates. Supplementary Figure 9. Prenatal exposure to folic acid supplements and the risk of childhood cancer. Abbreviations: ES, estimate; n.a., not available. Supplementary Figure 10. Prenatal exposure to hormones and the risk of childhood cancer. Abbreviations: ES, estimate; n.a., not available; 1estimates were calculated with four-square table; *c [file 12885_2025_15316_MOESM1_ESM.zip › Supplementary Figure 20 Prenatal exposure to antihypertensives and the risk of ALL and solid tumors in children_revised.docx]

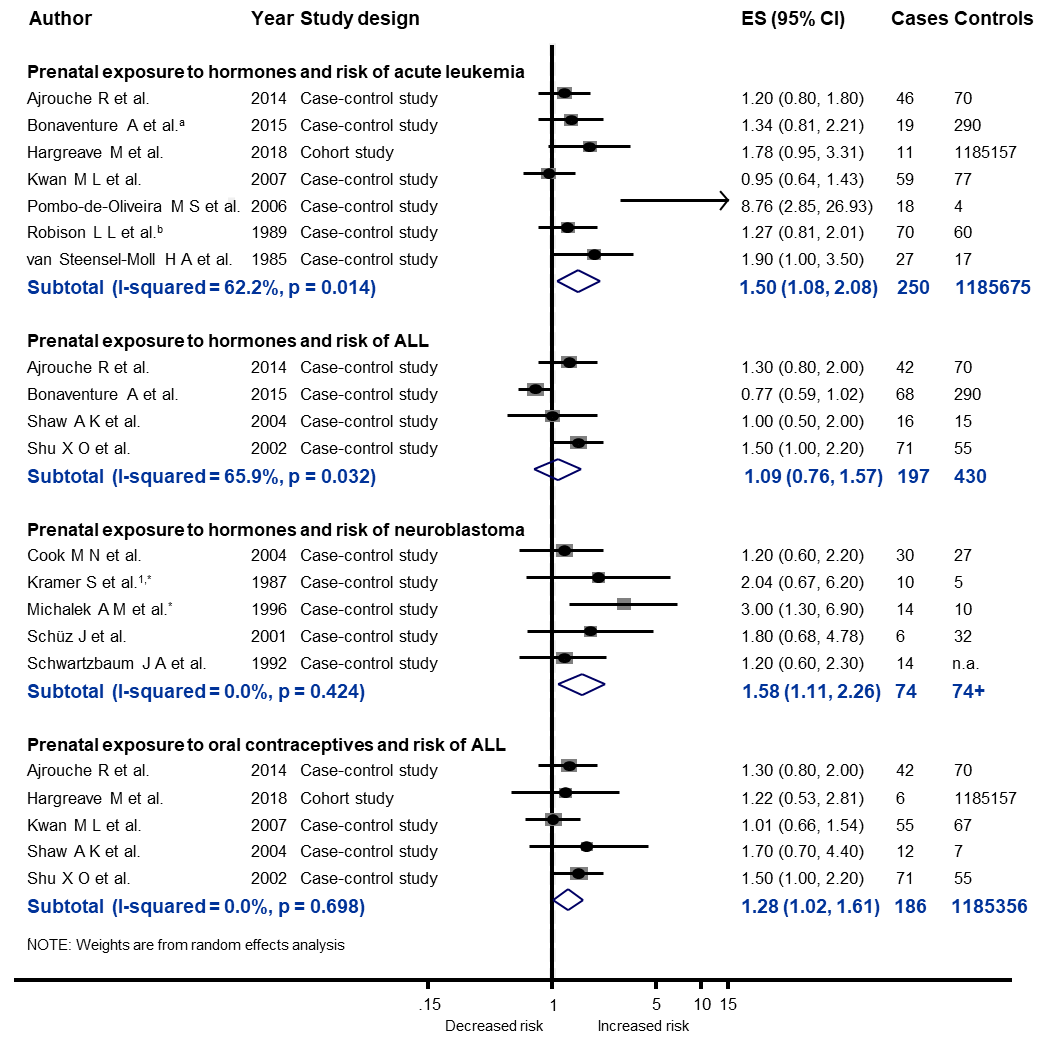

Supplement: Supplementary file 1 — Supplementary Material 1: Supplementary Figure 1. Prenatal exposure to analgesics and the risk of childhood cancer. Abbreviations: ES, estimate; n.a., not available. Supplementary Figure 2. Prenatal exposure to antibiotics and the risk of childhood cancer. Abbreviations: ES, estimate; 1estimates were calculated with four-square table; * calculation of crude estimates. Supplementary Figure 3. Prenatal exposure to antiemetics and the risk of childhood cancer. Abbreviations: ES, estimate; n.a., not available; 1estimates were calculated with four-square table; * calculation of crude estimates. Supplementary Figure 4. Prenatal exposure to antihistamines and the risk of childhood cancer. Abbreviations: ES, estimate; n.a., not available; 1estimates were calculated with four-square table; * calculation of crude estimates. Supplementary Figure 5. Prenatal exposure to antihypertensives and the risk of childhood cancer. Abbreviations: ES, estimate; n.a., not available. Supplementary Figure 6. Prenatal exposure to antiretroviral HIV-drugs and the risk of childhood cancer. Abbreviations: ES, estimate; n.a., not available; HIV, human immunodeficiency virus; * calculation of crude estimates. Supplementary Figure 7. Prenatal exposure to cold or cough remedies and the risk of childhood cancer. Abbreviations: ES, estimate; n.a., not available; 1estimates were calculated with four-square table; * calculation of crude estimates. Supplementary Figure 8. Prenatal exposure to diuretics and the risk of childhood cancer. Abbreviations: ES, estimate; n.a., not available; 1estimates were calculated with four-square table; *calculation of crude estimates. Supplementary Figure 9. Prenatal exposure to folic acid supplements and the risk of childhood cancer. Abbreviations: ES, estimate; n.a., not available. Supplementary Figure 10. Prenatal exposure to hormones and the risk of childhood cancer. Abbreviations: ES, estimate; n.a., not available; 1estimates were calculated with four-square table; *c [file 12885_2025_15316_MOESM1_ESM.zip › Supplementary Figure 21 Prenatal exposure to hormones and the risk of different childhood cancer sites_revised.docx]

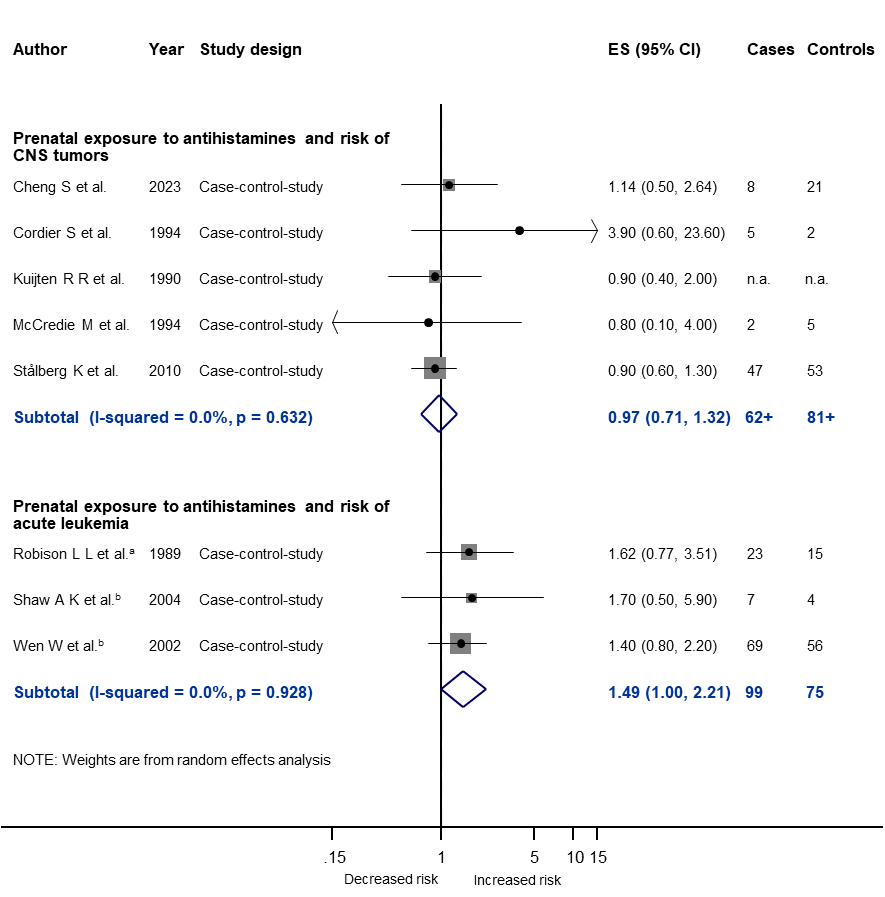

Supplement: Supplementary file 1 — Supplementary Material 1: Supplementary Figure 1. Prenatal exposure to analgesics and the risk of childhood cancer. Abbreviations: ES, estimate; n.a., not available. Supplementary Figure 2. Prenatal exposure to antibiotics and the risk of childhood cancer. Abbreviations: ES, estimate; 1estimates were calculated with four-square table; * calculation of crude estimates. Supplementary Figure 3. Prenatal exposure to antiemetics and the risk of childhood cancer. Abbreviations: ES, estimate; n.a., not available; 1estimates were calculated with four-square table; * calculation of crude estimates. Supplementary Figure 4. Prenatal exposure to antihistamines and the risk of childhood cancer. Abbreviations: ES, estimate; n.a., not available; 1estimates were calculated with four-square table; * calculation of crude estimates. Supplementary Figure 5. Prenatal exposure to antihypertensives and the risk of childhood cancer. Abbreviations: ES, estimate; n.a., not available. Supplementary Figure 6. Prenatal exposure to antiretroviral HIV-drugs and the risk of childhood cancer. Abbreviations: ES, estimate; n.a., not available; HIV, human immunodeficiency virus; * calculation of crude estimates. Supplementary Figure 7. Prenatal exposure to cold or cough remedies and the risk of childhood cancer. Abbreviations: ES, estimate; n.a., not available; 1estimates were calculated with four-square table; * calculation of crude estimates. Supplementary Figure 8. Prenatal exposure to diuretics and the risk of childhood cancer. Abbreviations: ES, estimate; n.a., not available; 1estimates were calculated with four-square table; *calculation of crude estimates. Supplementary Figure 9. Prenatal exposure to folic acid supplements and the risk of childhood cancer. Abbreviations: ES, estimate; n.a., not available. Supplementary Figure 10. Prenatal exposure to hormones and the risk of childhood cancer. Abbreviations: ES, estimate; n.a., not available; 1estimates were calculated with four-square table; *c [file 12885_2025_15316_MOESM1_ESM.zip › Supplementary Figure 22 Prenatal exposure to antihistamines and the risk of acute leukemia and CNS tumors in children_revised.docx]

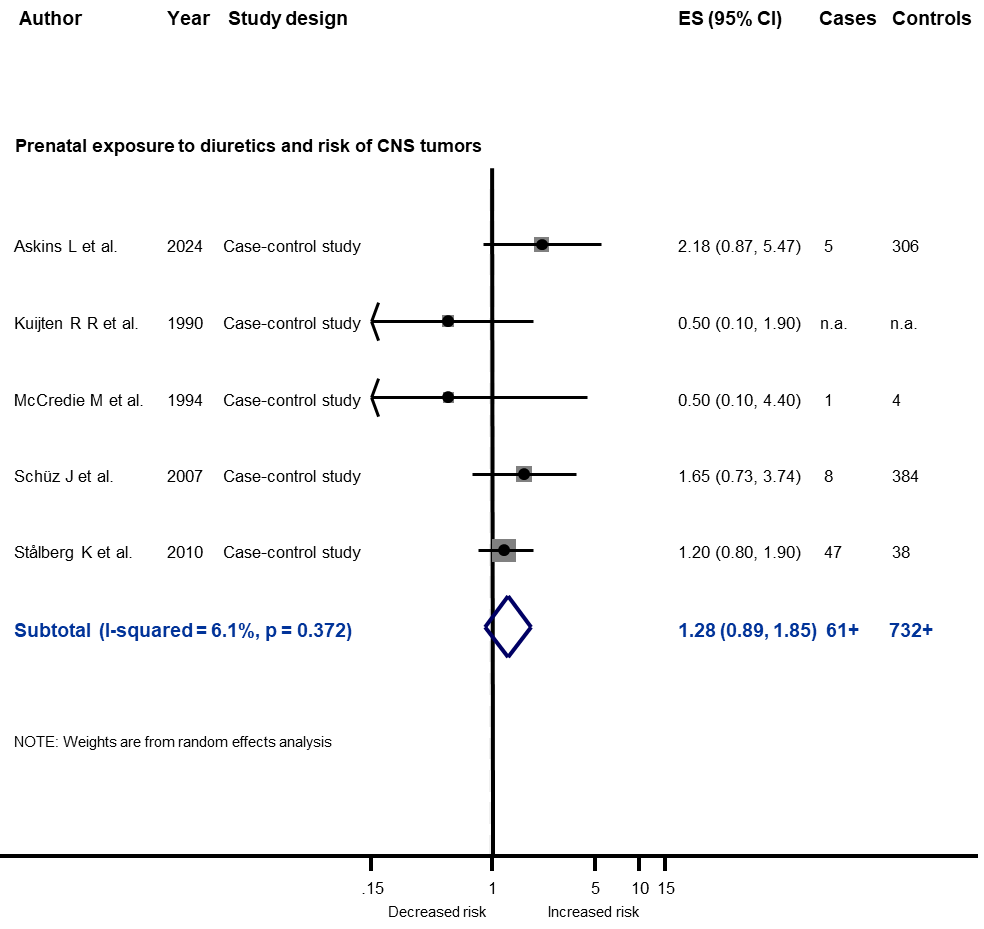

Supplement: Supplementary file 1 — Supplementary Material 1: Supplementary Figure 1. Prenatal exposure to analgesics and the risk of childhood cancer. Abbreviations: ES, estimate; n.a., not available. Supplementary Figure 2. Prenatal exposure to antibiotics and the risk of childhood cancer. Abbreviations: ES, estimate; 1estimates were calculated with four-square table; * calculation of crude estimates. Supplementary Figure 3. Prenatal exposure to antiemetics and the risk of childhood cancer. Abbreviations: ES, estimate; n.a., not available; 1estimates were calculated with four-square table; * calculation of crude estimates. Supplementary Figure 4. Prenatal exposure to antihistamines and the risk of childhood cancer. Abbreviations: ES, estimate; n.a., not available; 1estimates were calculated with four-square table; * calculation of crude estimates. Supplementary Figure 5. Prenatal exposure to antihypertensives and the risk of childhood cancer. Abbreviations: ES, estimate; n.a., not available. Supplementary Figure 6. Prenatal exposure to antiretroviral HIV-drugs and the risk of childhood cancer. Abbreviations: ES, estimate; n.a., not available; HIV, human immunodeficiency virus; * calculation of crude estimates. Supplementary Figure 7. Prenatal exposure to cold or cough remedies and the risk of childhood cancer. Abbreviations: ES, estimate; n.a., not available; 1estimates were calculated with four-square table; * calculation of crude estimates. Supplementary Figure 8. Prenatal exposure to diuretics and the risk of childhood cancer. Abbreviations: ES, estimate; n.a., not available; 1estimates were calculated with four-square table; *calculation of crude estimates. Supplementary Figure 9. Prenatal exposure to folic acid supplements and the risk of childhood cancer. Abbreviations: ES, estimate; n.a., not available. Supplementary Figure 10. Prenatal exposure to hormones and the risk of childhood cancer. Abbreviations: ES, estimate; n.a., not available; 1estimates were calculated with four-square table; *c [file 12885_2025_15316_MOESM1_ESM.zip › Supplementary Figure 23 Prenatal exposure to diuretics and the risk of CNS tumors in children_revised.docx]

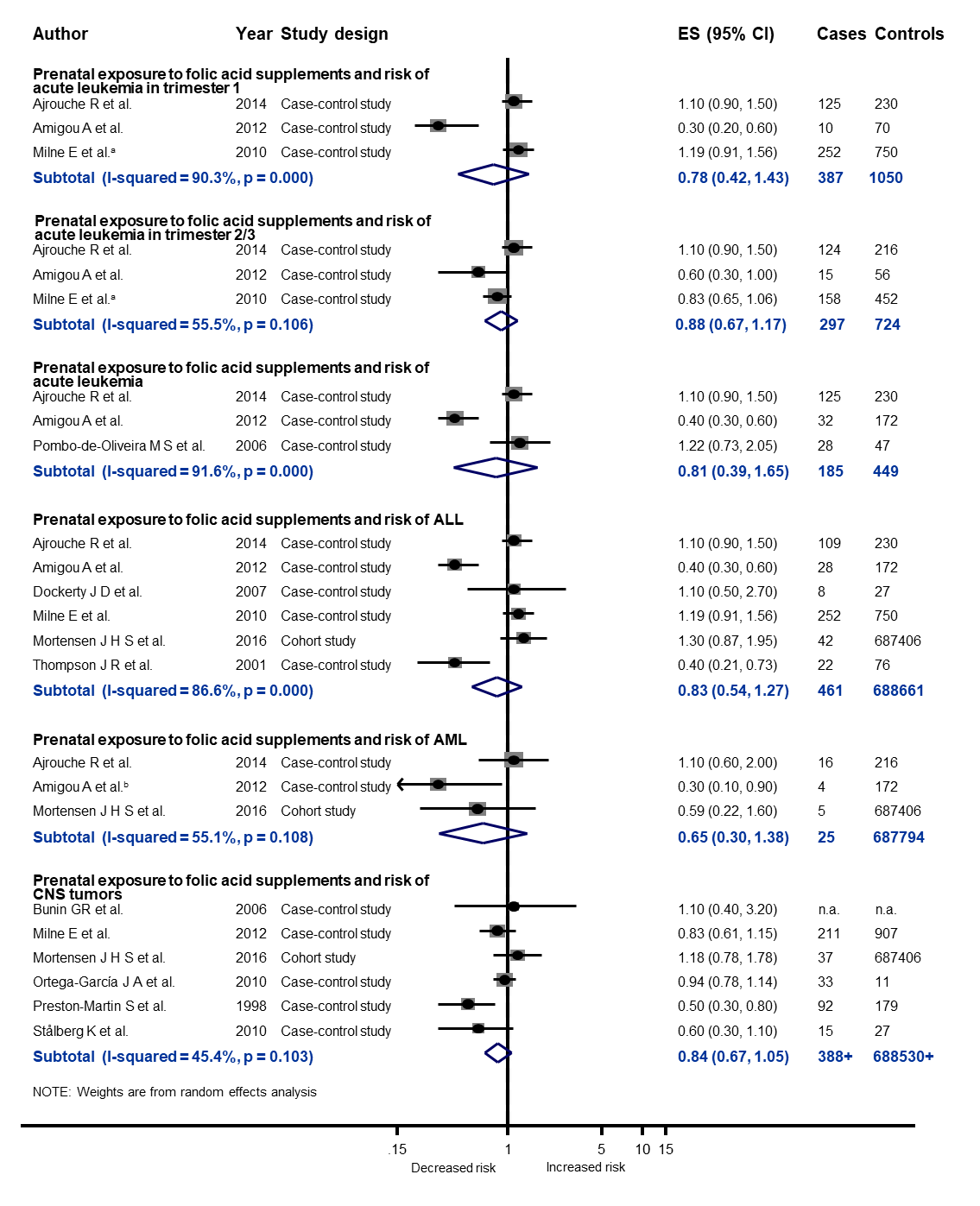

Supplement: Supplementary file 1 — Supplementary Material 1: Supplementary Figure 1. Prenatal exposure to analgesics and the risk of childhood cancer. Abbreviations: ES, estimate; n.a., not available. Supplementary Figure 2. Prenatal exposure to antibiotics and the risk of childhood cancer. Abbreviations: ES, estimate; 1estimates were calculated with four-square table; * calculation of crude estimates. Supplementary Figure 3. Prenatal exposure to antiemetics and the risk of childhood cancer. Abbreviations: ES, estimate; n.a., not available; 1estimates were calculated with four-square table; * calculation of crude estimates. Supplementary Figure 4. Prenatal exposure to antihistamines and the risk of childhood cancer. Abbreviations: ES, estimate; n.a., not available; 1estimates were calculated with four-square table; * calculation of crude estimates. Supplementary Figure 5. Prenatal exposure to antihypertensives and the risk of childhood cancer. Abbreviations: ES, estimate; n.a., not available. Supplementary Figure 6. Prenatal exposure to antiretroviral HIV-drugs and the risk of childhood cancer. Abbreviations: ES, estimate; n.a., not available; HIV, human immunodeficiency virus; * calculation of crude estimates. Supplementary Figure 7. Prenatal exposure to cold or cough remedies and the risk of childhood cancer. Abbreviations: ES, estimate; n.a., not available; 1estimates were calculated with four-square table; * calculation of crude estimates. Supplementary Figure 8. Prenatal exposure to diuretics and the risk of childhood cancer. Abbreviations: ES, estimate; n.a., not available; 1estimates were calculated with four-square table; *calculation of crude estimates. Supplementary Figure 9. Prenatal exposure to folic acid supplements and the risk of childhood cancer. Abbreviations: ES, estimate; n.a., not available. Supplementary Figure 10. Prenatal exposure to hormones and the risk of childhood cancer. Abbreviations: ES, estimate; n.a., not available; 1estimates were calculated with four-square table; *c [file 12885_2025_15316_MOESM1_ESM.zip › Supplementary Figure 24 Prenatal exposure to folic acid supplements and the risk of different childhood cancer sites_revised.docx]

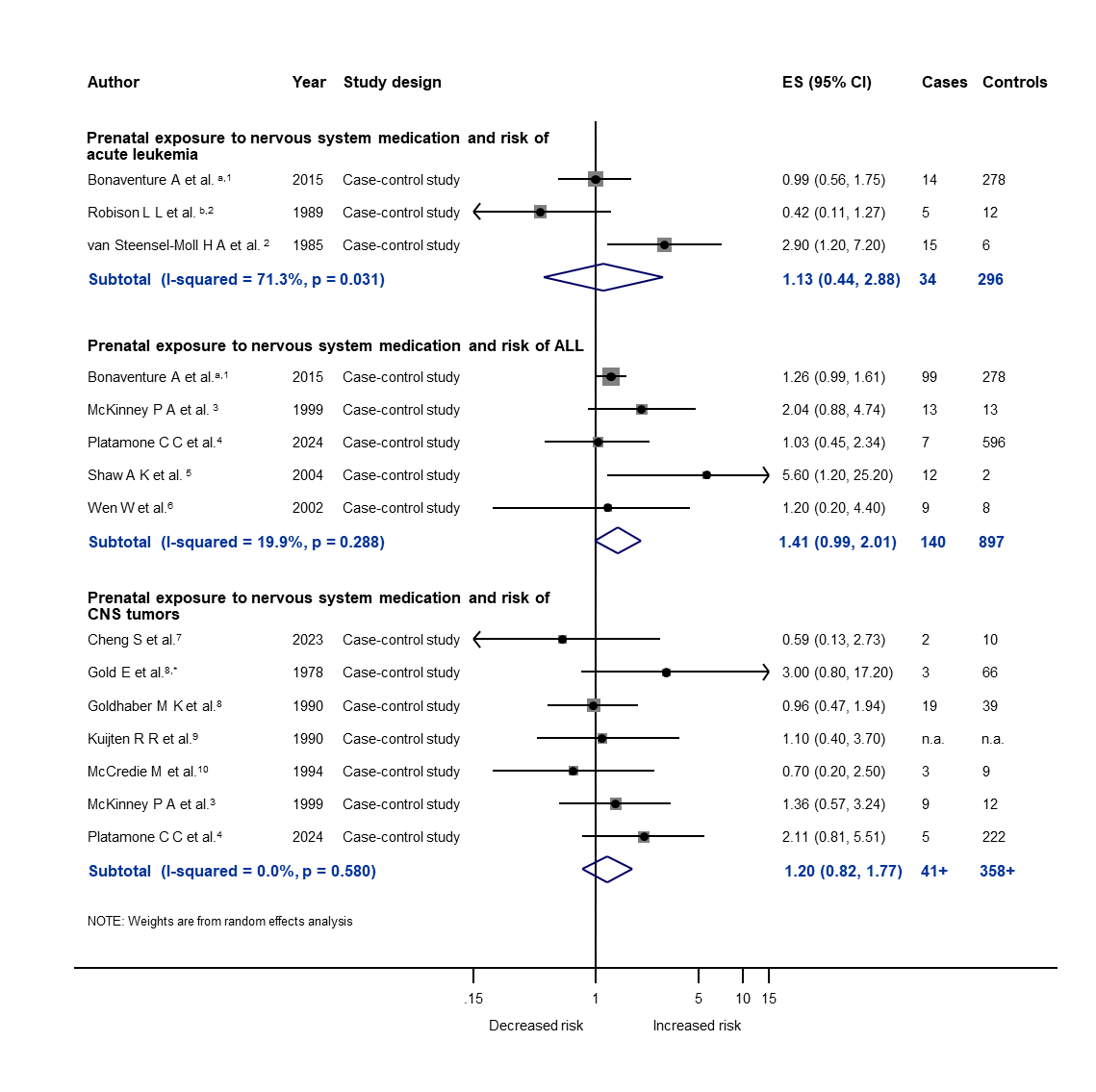

Supplement: Supplementary file 1 — Supplementary Material 1: Supplementary Figure 1. Prenatal exposure to analgesics and the risk of childhood cancer. Abbreviations: ES, estimate; n.a., not available. Supplementary Figure 2. Prenatal exposure to antibiotics and the risk of childhood cancer. Abbreviations: ES, estimate; 1estimates were calculated with four-square table; * calculation of crude estimates. Supplementary Figure 3. Prenatal exposure to antiemetics and the risk of childhood cancer. Abbreviations: ES, estimate; n.a., not available; 1estimates were calculated with four-square table; * calculation of crude estimates. Supplementary Figure 4. Prenatal exposure to antihistamines and the risk of childhood cancer. Abbreviations: ES, estimate; n.a., not available; 1estimates were calculated with four-square table; * calculation of crude estimates. Supplementary Figure 5. Prenatal exposure to antihypertensives and the risk of childhood cancer. Abbreviations: ES, estimate; n.a., not available. Supplementary Figure 6. Prenatal exposure to antiretroviral HIV-drugs and the risk of childhood cancer. Abbreviations: ES, estimate; n.a., not available; HIV, human immunodeficiency virus; * calculation of crude estimates. Supplementary Figure 7. Prenatal exposure to cold or cough remedies and the risk of childhood cancer. Abbreviations: ES, estimate; n.a., not available; 1estimates were calculated with four-square table; * calculation of crude estimates. Supplementary Figure 8. Prenatal exposure to diuretics and the risk of childhood cancer. Abbreviations: ES, estimate; n.a., not available; 1estimates were calculated with four-square table; *calculation of crude estimates. Supplementary Figure 9. Prenatal exposure to folic acid supplements and the risk of childhood cancer. Abbreviations: ES, estimate; n.a., not available. Supplementary Figure 10. Prenatal exposure to hormones and the risk of childhood cancer. Abbreviations: ES, estimate; n.a., not available; 1estimates were calculated with four-square table; *c [file 12885_2025_15316_MOESM1_ESM.zip › Supplementary Figure 25 Prenatal exposure to nervous system medication and the risk of different childhood cancer sites_revised.docx]

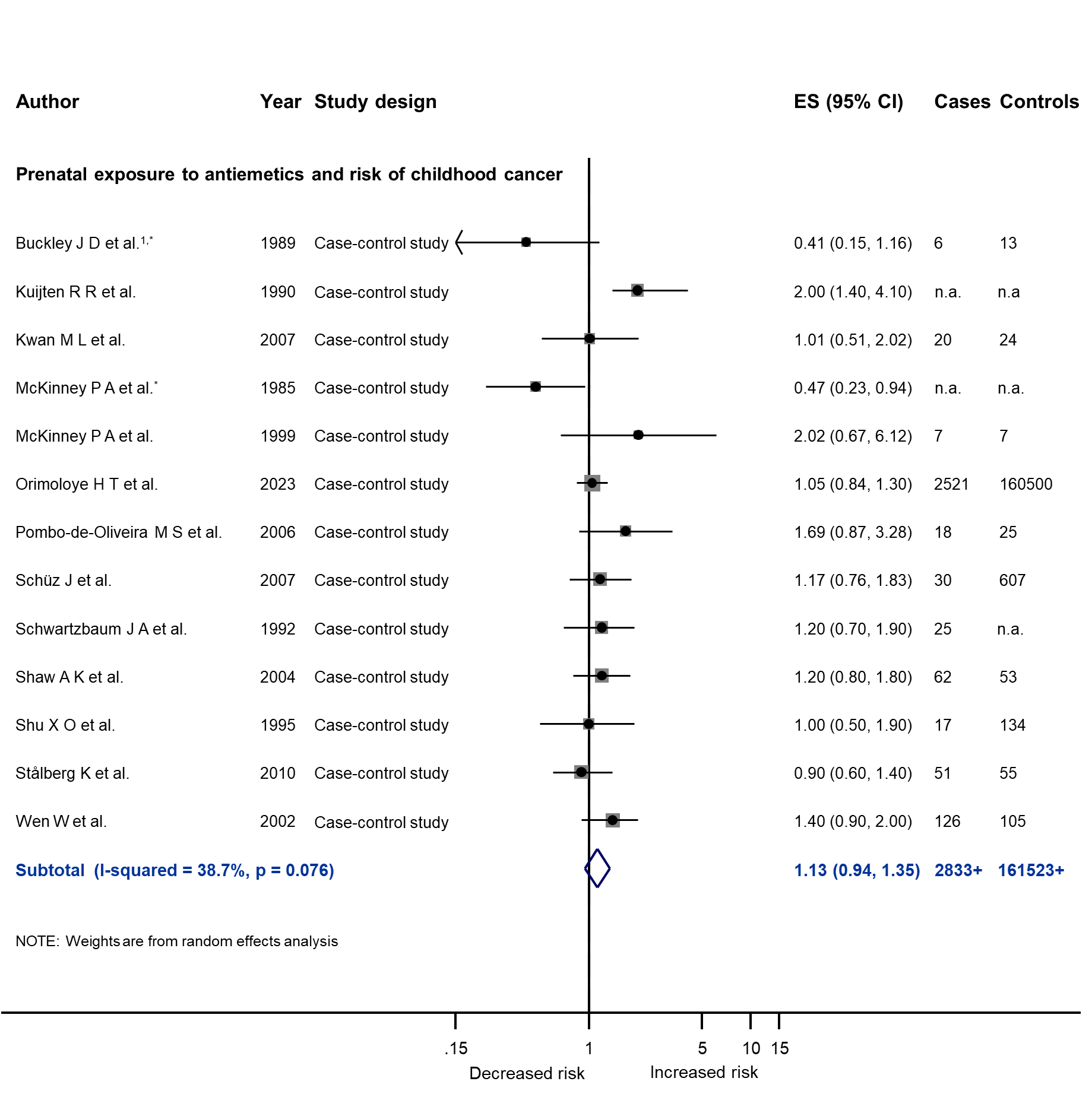

Supplement: Supplementary file 1 — Supplementary Material 1: Supplementary Figure 1. Prenatal exposure to analgesics and the risk of childhood cancer. Abbreviations: ES, estimate; n.a., not available. Supplementary Figure 2. Prenatal exposure to antibiotics and the risk of childhood cancer. Abbreviations: ES, estimate; 1estimates were calculated with four-square table; * calculation of crude estimates. Supplementary Figure 3. Prenatal exposure to antiemetics and the risk of childhood cancer. Abbreviations: ES, estimate; n.a., not available; 1estimates were calculated with four-square table; * calculation of crude estimates. Supplementary Figure 4. Prenatal exposure to antihistamines and the risk of childhood cancer. Abbreviations: ES, estimate; n.a., not available; 1estimates were calculated with four-square table; * calculation of crude estimates. Supplementary Figure 5. Prenatal exposure to antihypertensives and the risk of childhood cancer. Abbreviations: ES, estimate; n.a., not available. Supplementary Figure 6. Prenatal exposure to antiretroviral HIV-drugs and the risk of childhood cancer. Abbreviations: ES, estimate; n.a., not available; HIV, human immunodeficiency virus; * calculation of crude estimates. Supplementary Figure 7. Prenatal exposure to cold or cough remedies and the risk of childhood cancer. Abbreviations: ES, estimate; n.a., not available; 1estimates were calculated with four-square table; * calculation of crude estimates. Supplementary Figure 8. Prenatal exposure to diuretics and the risk of childhood cancer. Abbreviations: ES, estimate; n.a., not available; 1estimates were calculated with four-square table; *calculation of crude estimates. Supplementary Figure 9. Prenatal exposure to folic acid supplements and the risk of childhood cancer. Abbreviations: ES, estimate; n.a., not available. Supplementary Figure 10. Prenatal exposure to hormones and the risk of childhood cancer. Abbreviations: ES, estimate; n.a., not available; 1estimates were calculated with four-square table; *c [file 12885_2025_15316_MOESM1_ESM.zip › Supplementary Figure 3 Prenatal exposure to antiemetics and the risk of childhood cancer_revised.docx]

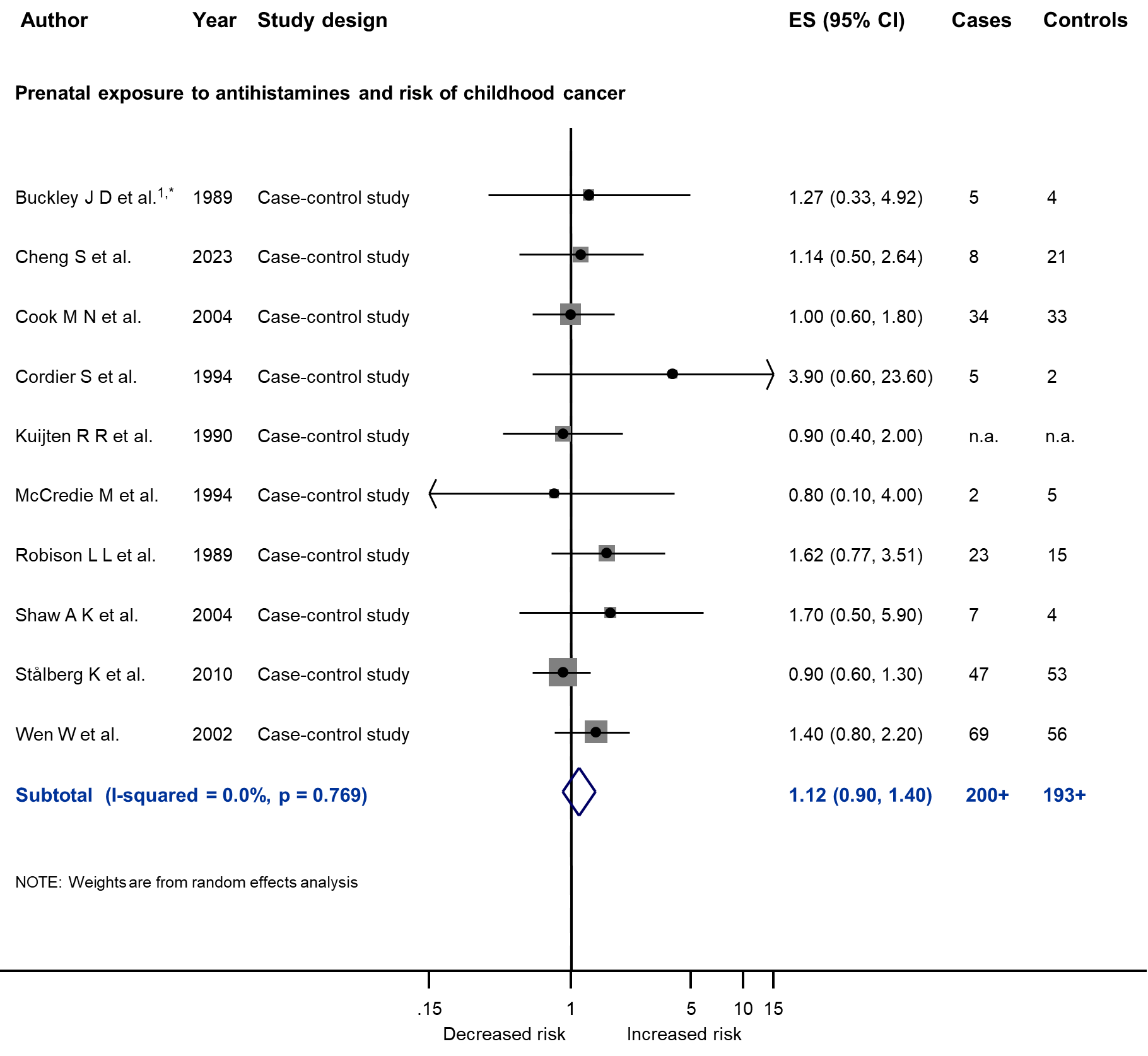

Supplement: Supplementary file 1 — Supplementary Material 1: Supplementary Figure 1. Prenatal exposure to analgesics and the risk of childhood cancer. Abbreviations: ES, estimate; n.a., not available. Supplementary Figure 2. Prenatal exposure to antibiotics and the risk of childhood cancer. Abbreviations: ES, estimate; 1estimates were calculated with four-square table; * calculation of crude estimates. Supplementary Figure 3. Prenatal exposure to antiemetics and the risk of childhood cancer. Abbreviations: ES, estimate; n.a., not available; 1estimates were calculated with four-square table; * calculation of crude estimates. Supplementary Figure 4. Prenatal exposure to antihistamines and the risk of childhood cancer. Abbreviations: ES, estimate; n.a., not available; 1estimates were calculated with four-square table; * calculation of crude estimates. Supplementary Figure 5. Prenatal exposure to antihypertensives and the risk of childhood cancer. Abbreviations: ES, estimate; n.a., not available. Supplementary Figure 6. Prenatal exposure to antiretroviral HIV-drugs and the risk of childhood cancer. Abbreviations: ES, estimate; n.a., not available; HIV, human immunodeficiency virus; * calculation of crude estimates. Supplementary Figure 7. Prenatal exposure to cold or cough remedies and the risk of childhood cancer. Abbreviations: ES, estimate; n.a., not available; 1estimates were calculated with four-square table; * calculation of crude estimates. Supplementary Figure 8. Prenatal exposure to diuretics and the risk of childhood cancer. Abbreviations: ES, estimate; n.a., not available; 1estimates were calculated with four-square table; *calculation of crude estimates. Supplementary Figure 9. Prenatal exposure to folic acid supplements and the risk of childhood cancer. Abbreviations: ES, estimate; n.a., not available. Supplementary Figure 10. Prenatal exposure to hormones and the risk of childhood cancer. Abbreviations: ES, estimate; n.a., not available; 1estimates were calculated with four-square table; *c [file 12885_2025_15316_MOESM1_ESM.zip › Supplementary Figure 4 Prenatal exposure to antihistamines and the risk of childhood cancer_revised.docx]

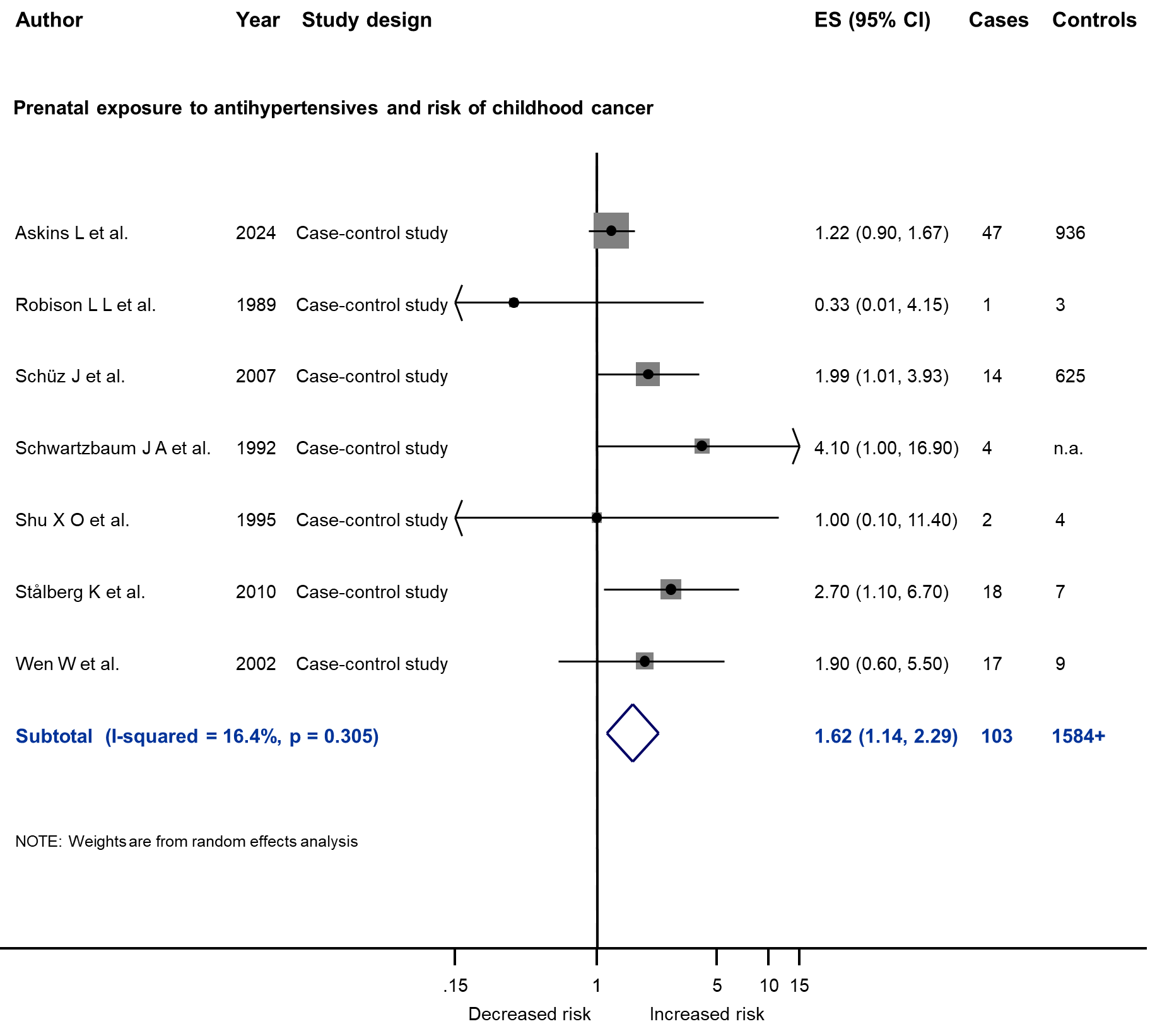

Supplement: Supplementary file 1 — Supplementary Material 1: Supplementary Figure 1. Prenatal exposure to analgesics and the risk of childhood cancer. Abbreviations: ES, estimate; n.a., not available. Supplementary Figure 2. Prenatal exposure to antibiotics and the risk of childhood cancer. Abbreviations: ES, estimate; 1estimates were calculated with four-square table; * calculation of crude estimates. Supplementary Figure 3. Prenatal exposure to antiemetics and the risk of childhood cancer. Abbreviations: ES, estimate; n.a., not available; 1estimates were calculated with four-square table; * calculation of crude estimates. Supplementary Figure 4. Prenatal exposure to antihistamines and the risk of childhood cancer. Abbreviations: ES, estimate; n.a., not available; 1estimates were calculated with four-square table; * calculation of crude estimates. Supplementary Figure 5. Prenatal exposure to antihypertensives and the risk of childhood cancer. Abbreviations: ES, estimate; n.a., not available. Supplementary Figure 6. Prenatal exposure to antiretroviral HIV-drugs and the risk of childhood cancer. Abbreviations: ES, estimate; n.a., not available; HIV, human immunodeficiency virus; * calculation of crude estimates. Supplementary Figure 7. Prenatal exposure to cold or cough remedies and the risk of childhood cancer. Abbreviations: ES, estimate; n.a., not available; 1estimates were calculated with four-square table; * calculation of crude estimates. Supplementary Figure 8. Prenatal exposure to diuretics and the risk of childhood cancer. Abbreviations: ES, estimate; n.a., not available; 1estimates were calculated with four-square table; *calculation of crude estimates. Supplementary Figure 9. Prenatal exposure to folic acid supplements and the risk of childhood cancer. Abbreviations: ES, estimate; n.a., not available. Supplementary Figure 10. Prenatal exposure to hormones and the risk of childhood cancer. Abbreviations: ES, estimate; n.a., not available; 1estimates were calculated with four-square table; *c [file 12885_2025_15316_MOESM1_ESM.zip › Supplementary Figure 5 Prenatal exposure to antihypertensives and the risk of childhood cancer.docx]

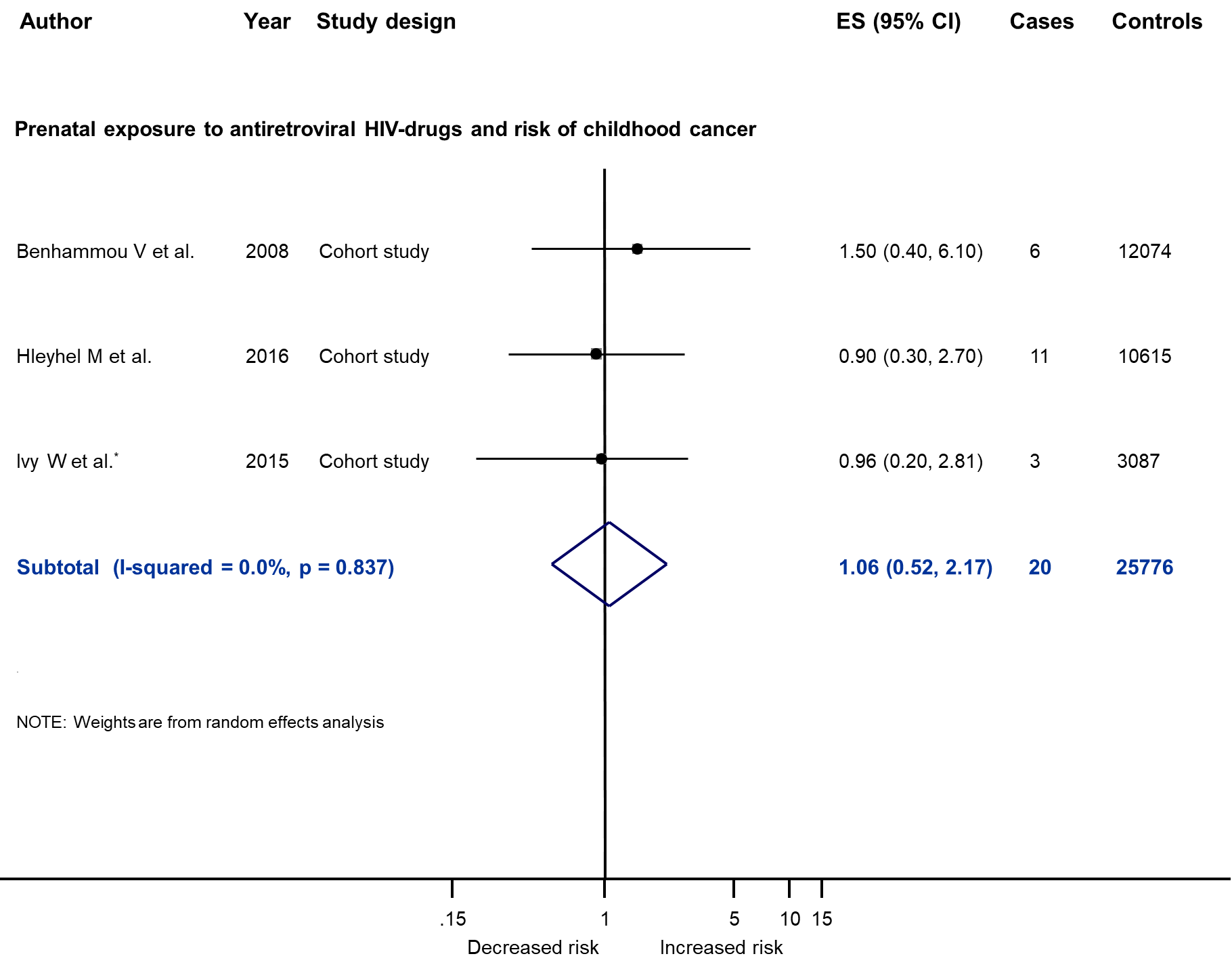

Supplement: Supplementary file 1 — Supplementary Material 1: Supplementary Figure 1. Prenatal exposure to analgesics and the risk of childhood cancer. Abbreviations: ES, estimate; n.a., not available. Supplementary Figure 2. Prenatal exposure to antibiotics and the risk of childhood cancer. Abbreviations: ES, estimate; 1estimates were calculated with four-square table; * calculation of crude estimates. Supplementary Figure 3. Prenatal exposure to antiemetics and the risk of childhood cancer. Abbreviations: ES, estimate; n.a., not available; 1estimates were calculated with four-square table; * calculation of crude estimates. Supplementary Figure 4. Prenatal exposure to antihistamines and the risk of childhood cancer. Abbreviations: ES, estimate; n.a., not available; 1estimates were calculated with four-square table; * calculation of crude estimates. Supplementary Figure 5. Prenatal exposure to antihypertensives and the risk of childhood cancer. Abbreviations: ES, estimate; n.a., not available. Supplementary Figure 6. Prenatal exposure to antiretroviral HIV-drugs and the risk of childhood cancer. Abbreviations: ES, estimate; n.a., not available; HIV, human immunodeficiency virus; * calculation of crude estimates. Supplementary Figure 7. Prenatal exposure to cold or cough remedies and the risk of childhood cancer. Abbreviations: ES, estimate; n.a., not available; 1estimates were calculated with four-square table; * calculation of crude estimates. Supplementary Figure 8. Prenatal exposure to diuretics and the risk of childhood cancer. Abbreviations: ES, estimate; n.a., not available; 1estimates were calculated with four-square table; *calculation of crude estimates. Supplementary Figure 9. Prenatal exposure to folic acid supplements and the risk of childhood cancer. Abbreviations: ES, estimate; n.a., not available. Supplementary Figure 10. Prenatal exposure to hormones and the risk of childhood cancer. Abbreviations: ES, estimate; n.a., not available; 1estimates were calculated with four-square table; *c [file 12885_2025_15316_MOESM1_ESM.zip › Supplementary Figure 6 Prenatal exposure to antiretroviral HIV-drugs and the risk of childhood cancer_revised.docx]

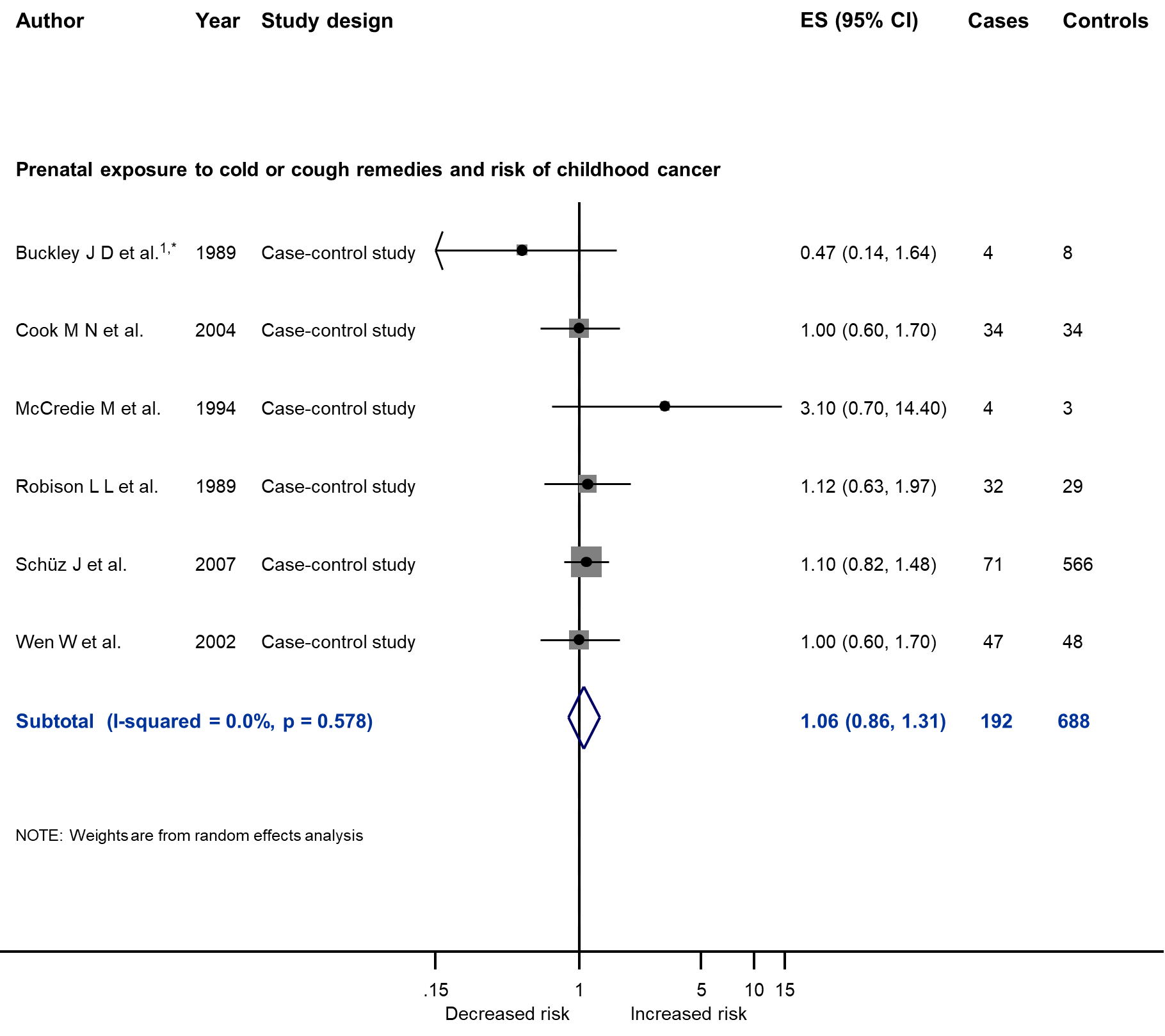

Supplement: Supplementary file 1 — Supplementary Material 1: Supplementary Figure 1. Prenatal exposure to analgesics and the risk of childhood cancer. Abbreviations: ES, estimate; n.a., not available. Supplementary Figure 2. Prenatal exposure to antibiotics and the risk of childhood cancer. Abbreviations: ES, estimate; 1estimates were calculated with four-square table; * calculation of crude estimates. Supplementary Figure 3. Prenatal exposure to antiemetics and the risk of childhood cancer. Abbreviations: ES, estimate; n.a., not available; 1estimates were calculated with four-square table; * calculation of crude estimates. Supplementary Figure 4. Prenatal exposure to antihistamines and the risk of childhood cancer. Abbreviations: ES, estimate; n.a., not available; 1estimates were calculated with four-square table; * calculation of crude estimates. Supplementary Figure 5. Prenatal exposure to antihypertensives and the risk of childhood cancer. Abbreviations: ES, estimate; n.a., not available. Supplementary Figure 6. Prenatal exposure to antiretroviral HIV-drugs and the risk of childhood cancer. Abbreviations: ES, estimate; n.a., not available; HIV, human immunodeficiency virus; * calculation of crude estimates. Supplementary Figure 7. Prenatal exposure to cold or cough remedies and the risk of childhood cancer. Abbreviations: ES, estimate; n.a., not available; 1estimates were calculated with four-square table; * calculation of crude estimates. Supplementary Figure 8. Prenatal exposure to diuretics and the risk of childhood cancer. Abbreviations: ES, estimate; n.a., not available; 1estimates were calculated with four-square table; *calculation of crude estimates. Supplementary Figure 9. Prenatal exposure to folic acid supplements and the risk of childhood cancer. Abbreviations: ES, estimate; n.a., not available. Supplementary Figure 10. Prenatal exposure to hormones and the risk of childhood cancer. Abbreviations: ES, estimate; n.a., not available; 1estimates were calculated with four-square table; *c [file 12885_2025_15316_MOESM1_ESM.zip › Supplementary Figure 7 Prenatal exposure to cold or cough remedies and the risk of childhood cancer_revised.docx]

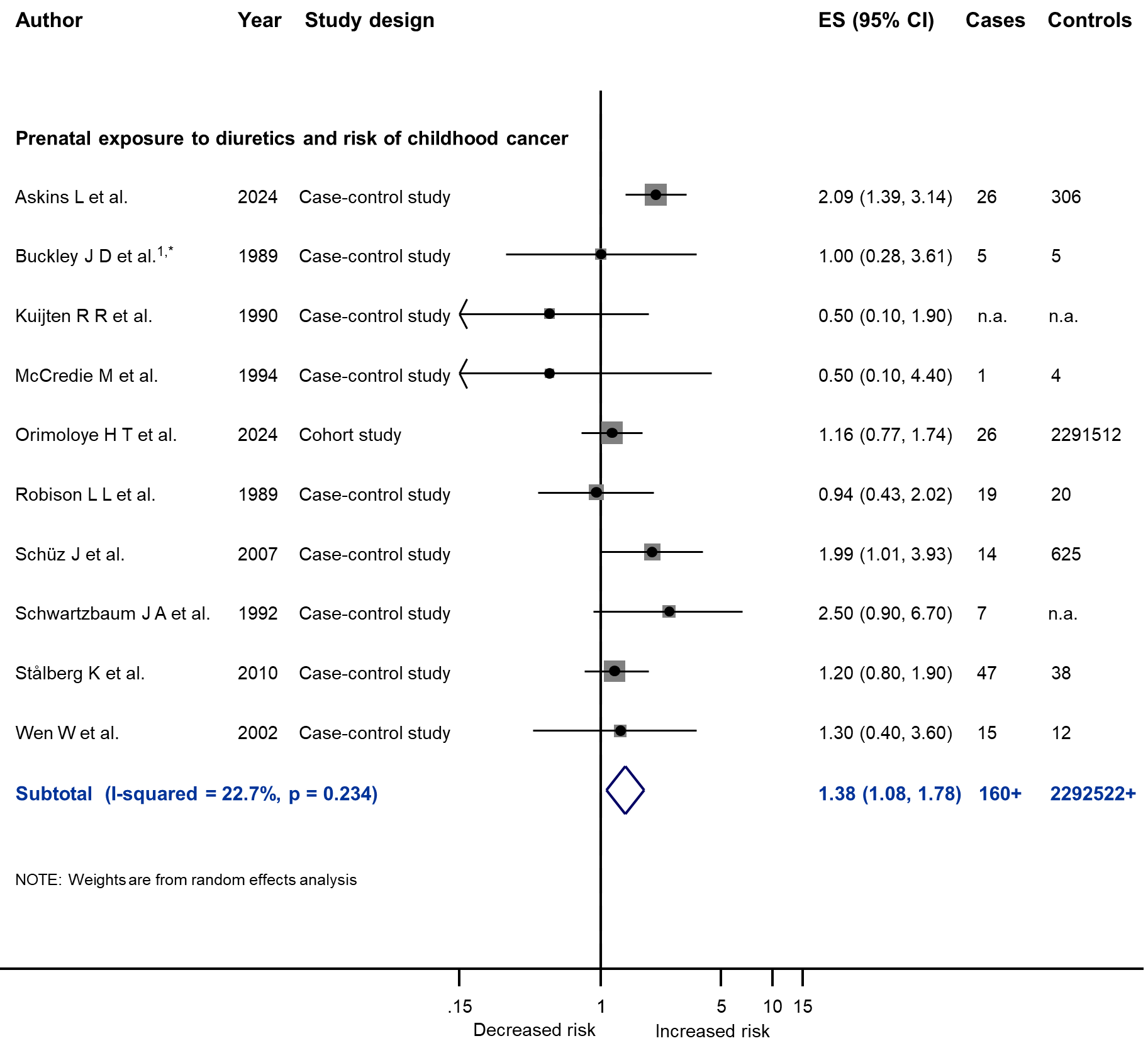

Supplement: Supplementary file 1 — Supplementary Material 1: Supplementary Figure 1. Prenatal exposure to analgesics and the risk of childhood cancer. Abbreviations: ES, estimate; n.a., not available. Supplementary Figure 2. Prenatal exposure to antibiotics and the risk of childhood cancer. Abbreviations: ES, estimate; 1estimates were calculated with four-square table; * calculation of crude estimates. Supplementary Figure 3. Prenatal exposure to antiemetics and the risk of childhood cancer. Abbreviations: ES, estimate; n.a., not available; 1estimates were calculated with four-square table; * calculation of crude estimates. Supplementary Figure 4. Prenatal exposure to antihistamines and the risk of childhood cancer. Abbreviations: ES, estimate; n.a., not available; 1estimates were calculated with four-square table; * calculation of crude estimates. Supplementary Figure 5. Prenatal exposure to antihypertensives and the risk of childhood cancer. Abbreviations: ES, estimate; n.a., not available. Supplementary Figure 6. Prenatal exposure to antiretroviral HIV-drugs and the risk of childhood cancer. Abbreviations: ES, estimate; n.a., not available; HIV, human immunodeficiency virus; * calculation of crude estimates. Supplementary Figure 7. Prenatal exposure to cold or cough remedies and the risk of childhood cancer. Abbreviations: ES, estimate; n.a., not available; 1estimates were calculated with four-square table; * calculation of crude estimates. Supplementary Figure 8. Prenatal exposure to diuretics and the risk of childhood cancer. Abbreviations: ES, estimate; n.a., not available; 1estimates were calculated with four-square table; *calculation of crude estimates. Supplementary Figure 9. Prenatal exposure to folic acid supplements and the risk of childhood cancer. Abbreviations: ES, estimate; n.a., not available. Supplementary Figure 10. Prenatal exposure to hormones and the risk of childhood cancer. Abbreviations: ES, estimate; n.a., not available; 1estimates were calculated with four-square table; *c [file 12885_2025_15316_MOESM1_ESM.zip › Supplementary Figure 8 Prenatal exposure to diuretics and the risk of childhood cancer_revised.docx]

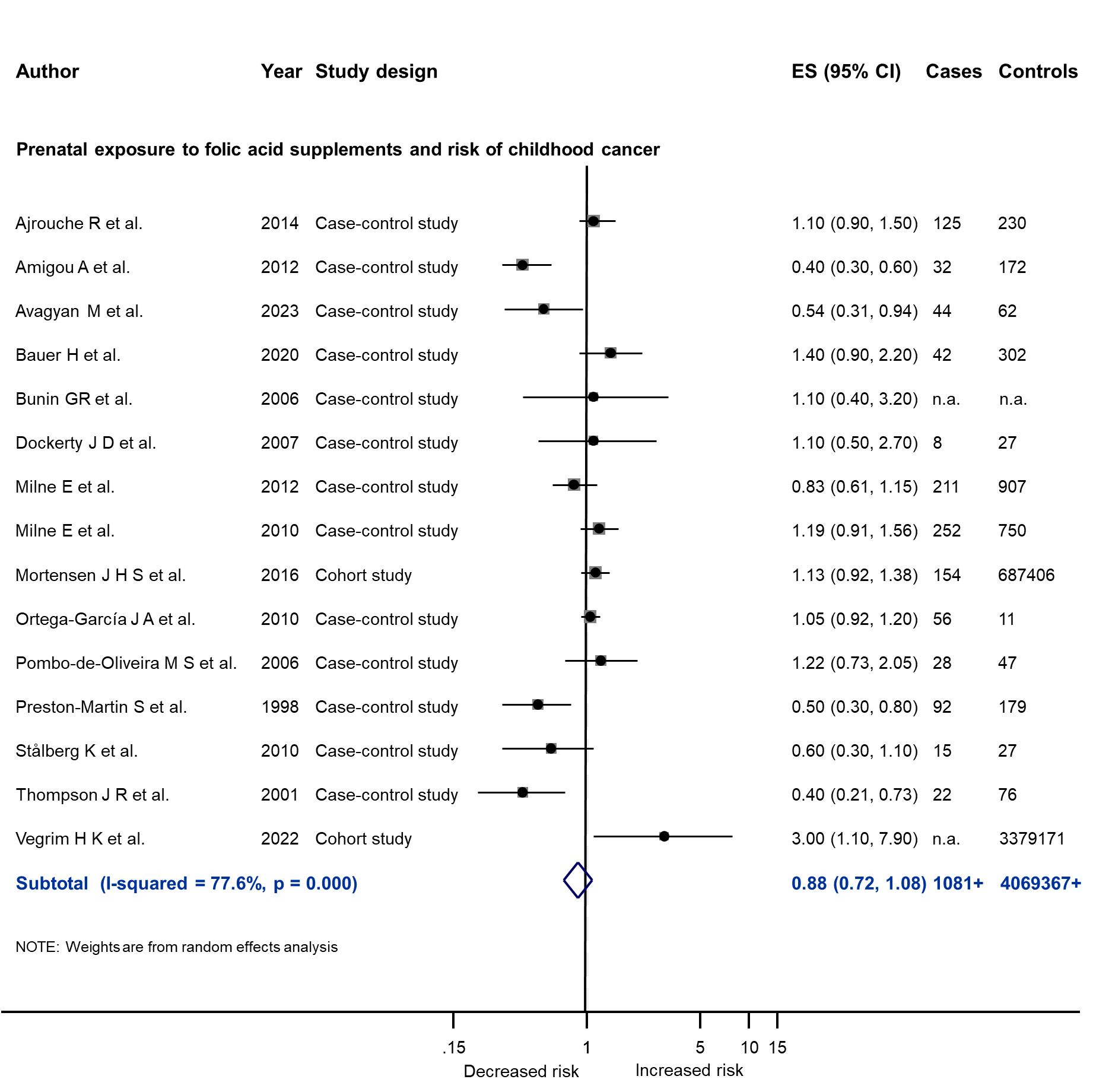

Supplement: Supplementary file 1 — Supplementary Material 1: Supplementary Figure 1. Prenatal exposure to analgesics and the risk of childhood cancer. Abbreviations: ES, estimate; n.a., not available. Supplementary Figure 2. Prenatal exposure to antibiotics and the risk of childhood cancer. Abbreviations: ES, estimate; 1estimates were calculated with four-square table; * calculation of crude estimates. Supplementary Figure 3. Prenatal exposure to antiemetics and the risk of childhood cancer. Abbreviations: ES, estimate; n.a., not available; 1estimates were calculated with four-square table; * calculation of crude estimates. Supplementary Figure 4. Prenatal exposure to antihistamines and the risk of childhood cancer. Abbreviations: ES, estimate; n.a., not available; 1estimates were calculated with four-square table; * calculation of crude estimates. Supplementary Figure 5. Prenatal exposure to antihypertensives and the risk of childhood cancer. Abbreviations: ES, estimate; n.a., not available. Supplementary Figure 6. Prenatal exposure to antiretroviral HIV-drugs and the risk of childhood cancer. Abbreviations: ES, estimate; n.a., not available; HIV, human immunodeficiency virus; * calculation of crude estimates. Supplementary Figure 7. Prenatal exposure to cold or cough remedies and the risk of childhood cancer. Abbreviations: ES, estimate; n.a., not available; 1estimates were calculated with four-square table; * calculation of crude estimates. Supplementary Figure 8. Prenatal exposure to diuretics and the risk of childhood cancer. Abbreviations: ES, estimate; n.a., not available; 1estimates were calculated with four-square table; *calculation of crude estimates. Supplementary Figure 9. Prenatal exposure to folic acid supplements and the risk of childhood cancer. Abbreviations: ES, estimate; n.a., not available. Supplementary Figure 10. Prenatal exposure to hormones and the risk of childhood cancer. Abbreviations: ES, estimate; n.a., not available; 1estimates were calculated with four-square table; *c [file 12885_2025_15316_MOESM1_ESM.zip › Supplementary Figure 9 Prenatal exposure to folic acid supplements and the risk of childhood cancer.docx]
